# Supplementary material for: Investigating the relationship between thought interference, somatic passivity and outcomes in patients with psychosis: a natural language processing approach using a clinical records search platform in south London
Source: BMJ Open. 2022 Aug 2;12(8):e057433. doi: 10.1136/bmjopen-2021-057433 (PMC9351333; doi:10.1136/bmjopen-2021-057433)
Supplement: Supplementary data [file bmjopen-2021-057433supp001.pdf]

Supplementary Material

Supplementary Table 1: Natural language processing algorithm development and performance. A minimum P and R of 80% is required for an algorithm to be considered reliable. In the context of passivity, the lower IRR led to the inclusion of extra annotations that enabled us to reach this threshold[1].

Symptom	**Keyword**	**Number of annotations**	**IRR**	**P and R**	**Extra steps required**	**Manual testing**	**Keyword search P and R**
Thought insertion	"thought insert*"	592 (96 double-annoted)	97 %	P=81%; R=94%	N/A	P=84%	P=81%; R=96%
Thought broadcasting	"thought broadcast*"	398 (95 double-annoted)	94 %	P=81%; R=94%	N/A	P=84%	P=81%; R=96%
Thought withdrawal	"thought withdraw*"	500 (76 double-annotated)	95 %	P=74%; R=91%	159 extra annotations	P=84%	P= 90%; R=88%
Somatic passivity	"passivity"	500 (116 double-annotated)	71 %	P=72%; R=92%	597 extra annotations, form excluded	P=82%	P=89%; R=100%

[1] P: Precision (proportion of positive entities identified by the NLP algorithm that are genuine entities on manual annotation); R: Recall (proportion of true positive entities on manual annotation that are identified as such by the NLP algorithm).

Magrangeas TT, *et al. BMJ Open* 2022; 12:e057433. doi: 10.1136/bmjopen-2021-057433

Supplementary Table 2: Overlap between thought broadcasting, thought insertion and thought withdrawal. The overlap observed before further analyses suggested that there may exist interactions linking these factors and directed our analyses towards the development of a model to understand these better.

Symptom	**Number of patients experiencing the symptom**
Thought insertion	997
Thought broadcasting	956
Thought withdrawal	459
Thought insertion and broadcasting	436
Thought insertion and withdrawal	243
Thought broadcasting and withdrawal	170
Thought insertion, broadcasting and withdrawal	123

Magrangeas TT, *et al. BMJ Open* 2022; 12:e057433. doi: 10.1136/bmjopen-2021-057433

Supplementary table 3: overlap between somatic passivity, thought interference and any other positive symptoms (auditory hallucinations, persecutory delusions, paranoia). When carrying out analyses, other positive symptoms were not combined but each included as individual covariates. The overlap observed here suggested that there may exist interactions linking these factors and directed our analyses towards the development of a model to understand these better. This table also reflects the prevalence of other positive symptoms and the need for investigations of the possible correlation between these and outcomes in the future.

Symptom	**Number of patients experiencing the symptom**
Somatic passivity	944
Thought interference	1686
Other positive symptoms	8181
Somatic passivity and thought interference	488
Somatic passivity and other positive symptoms	935
Interference and other positive symptoms	1664
Passivity, intereference and other positive symptoms	487

Magrangeas TT, *et al. BMJ Open* 2022; 12:e057433. doi: 10.1136/bmjopen-2021-057433

Supplementary Table 4: Unadjusted odds ratios for the co-occurrence of psychotic symptoms across the patient sample

Symptom	Broadcast	Insertion	Withdrawal	Passivity	Paranoia	Auditory Hallucinations	Persecutory Delusions
Broadcast	1						
Insertion	11.67	1					
Withdrawal	6.05	12.10	1				
Passivity	5.13	6.66	3.86	1			
Paranoia	5.29	3.97	3.32	4.67	1		
Auditory Hallucinations	5.33	5.06	3.45	4.99	3.50	1	
Persecutory Delusions	3.13	3.30	2.41	3.40	5.30	2.80	1

Magrangeas TT, *et al. BMJ Open* 2022; 12:e057433. doi: 10.1136/bmjopen-2021-057433

Supplementary table 5: overlap between outcomes investigated. This highlights the degree of overlap between negative outcomes, suggesting a possible correlation in their occurrence.

Outcome	**Number of patients with this outcome**
Presence of a mental health act section	3334
More than 22 days spent in crisis care	2331
More than 2 antipsychotics prescribed	2323
Mental health act section and more than 22 days spent in crisis care	1888
Mental health act section and more than 2 antipsychotics prescribed	1477
More than 22 days spent in crisis care and more than 2 antipsychotics prescribed	1245
Presence of all 3 outcomes	1085

Magrangeas TT, *et al. BMJ Open* 2022; 12:e057433. doi: 10.1136/bmjopen-2021-057433

Supplementary Table 6: Prevalence of negative outcomes in patients with somatic passivity or thought interference at different ages

Age (years)	Occurrence on negative outcomes in patients with somatic passivity (%) (95% CI)	Occurrence on negative outcomes in patients with thought interference (%) (95% CI)
20	59.6 (58.0-61.1)	59.1 (57.6-60.7)
30	53.7 (52.5-54.9)	53.4 (52.2-54.6)
40	47.7 (46.6-48.7)	47.7 (46.6-48.7)
50	41.7 (40.5-43.0)	41.9 (40.7-43.2)
60	36.1 (34.5-37.6)	36.4 (34.8-38.0)
70	30.7 (28.8-32.6)	31.2 (29.3-33.2)
80	25.9 (23.7-28.0)	26.5 (24.3-28.7)
90	21.5 (19.2-23.8)	22.2 (19.8-24.6)

Supplementary Table 7: Linear regression describing the influence of somatic passivity, thought interference and its subcomponents on the number of antipsychotics prescribed

Symptom	Standardized Beta Coefficients (95% CI)	t	Significance
Somatic Passivity	0.35 (0.24-0.45)	6.43	0.00
Thought Interference	0.40 (-0.14-0.22)	0.43	0.67
Thought Insertion	0.14 (0-0.01-0.30)	1.81	0.07
Thought Withdrawal	-0.00 (-0.17-0.16)	-0.05	0.96
Thought Broadcasting	-0.02 (-0.18-0.13)	-0.29	0.77

Adjusted for age at diagnosis, gender, ethnicity, diagnosis, IMD quartile and presence of paranoia, auditory hallucinations, persecutory delusion.

Magrangeas TT, *et al. BMJ Open* 2022; 12:e057433. doi: 10.1136/bmjopen-2021-057433
