# Supplementary material for: Investigating the relationship between thought interference, somatic passivity and outcomes in patients with psychosis: a natural language processing approach using a clinical records search platform in south London
Source: BMJ Open. 2022 Aug 2;12(8):e057433. doi: 10.1136/bmjopen-2021-057433 (PMC9351333; doi:10.1136/bmjopen-2021-057433)
Supplement: Author's manuscript [file bmjopen-2021-057433.draft_revisions.pdf]

BMJ Open is committed to open peer review. As part of this commitment we make the peer review history of every article we publish publicly available.

When an article is published we post the peer reviewers' comments and the authors' responses online. We also post the versions of the paper that were used during peer review. These are the versions that the peer review comments apply to.

The versions of the paper that follow are the versions that were submitted during the peer review process. They are not the versions of record or the final published versions. They should not be cited or distributed as the published version of this manuscript.

BMJ Open is an open access journal and the full, final, typeset and author-corrected version of record of the manuscript is available on our site with no access controls, subscription charges or pay-per-view fees (http://bmjopen.bmj.com).

If you have any questions on BMJ Open's open peer review process please email
info.bmjopen@bmj.com

BMJ Open

Investigating the influence of Thought Interference and Somatic Passivity on outcomes in patients with Psychosis: a Natural-Language Processing (NLP) approach using the Clinical Record Interactive Search (CRIS) platform

Journal:	*BMJ Open*
Manuscript ID	bmjopen-2021-057433
Article Type:	Original research
Date Submitted by the Author:	17-Sep-2021

Complete List of Authors:	Magrangeas, Thibault; King's College London Institute of Psychiatry Psychology and Neuroscience<br>Kolliakou, Anna; King's College London Institute of Psychiatry Psychology and Neuroscience, Institute of Psychiatry<br>Sanyal, Jyoti; South London and Maudsley NHS Foundation Trust<br>Patel, Rashmi; King's College London Institute of Psychiatry Psychology and Neuroscience; South London and Maudsley NHS Foundation Trust<br>Stewart, Robert; King's College London Institute of Psychiatry Psychology and Neuroscience; South London and Maudsley NHS Foundation Trust

Keywords:	Schizophrenia & psychotic disorders < PSYCHIATRY, Adult psychiatry < PSYCHIATRY, Health informatics < BIOTECHNOLOGY & BIOINFORMATICS

SCHOLARONE™
Manuscripts

I, the Submitting Author has the right to grant and does grant on behalf of all authors of the Work (as defined in the below author licence), an exclusive licence and/or a non-exclusive licence for contributions from authors who are: i) UK Crown employees; ii) where BMJ has agreed a CC-BY licence shall apply, and/or iii) in accordance with the terms applicable for US Federal Government officers or employees acting as part of their official duties; on a worldwide, perpetual, irrevocable, royalty-free basis to BMJ Publishing Group Ltd ("BMJ") its licensees and where the relevant Journal is co-owned by BMJ to the co-owners of the Journal, to publish the Work in this journal and any other BMJ products and to exploit all rights, as set out in our licence.

The Submitting Author accepts and understands that any supply made under these terms is made by BMJ to the Submitting Author unless you are acting as an employee on behalf of your employer or a postgraduate student of an affiliated institution which is paying any applicable article publishing charge ("APC") for Open Access articles. Where the Submitting Author wishes to make the Work available on an Open Access basis (and intends to pay the relevant APC), the terms of reuse of such Open Access shall be governed by a Creative Commons licence – details of these licences and which Creative Commons licence will apply to this Work are set out in our licence referred to above.

Other than as permitted in any relevant BMJ Author's Self Archiving Policies, I confirm this Work has not been accepted for publication elsewhere, is not being considered for publication elsewhere and does not duplicate material already published. I confirm all authors consent to publication of this Work and authorise the granting of this licence.

For peer review only

Title: Investigating the influence of Thought Interference and Somatic Passivity on outcomes in patients with Psychosis: a Natural-Language Processing (NLP) approach using the Clinical Record Interactive Search (CRIS) platform

Authors: Thibault Magrangeas[1], Anna Kolliakou[1], Jyoti Sanyal[2], Rashmi Patel[1&2], Robert Stewart[1&2]

Affiliations: 1: King's College London (Institute of Psychiatry, Psychology and Neuroscience), London, UK; 2: South London and Maudsley NHS Foundation Trust, London, UK

Corresponding Author: Thibault Magrangeas

Department of Psychological Medicine

Main Building E3.08

Institute of Psychiatry, Psychology and Neuroscience

King's College London

De Crespigny Park

London SE5 8AF

United Kingdom

Telephone: 00447761280858. **Fax:** N/A. **E-mail:** thibault.magrangeas@kcl.ac.uk c/o R Stewart's research group.

Abstract Word Count: 217

Body Word Count: 3963

Abstract

Objectives: Of the many studies describing psychotic symptoms in schizophrenia, few investigate their direct influence on prognosis. We aimed to apply natural language-processing (NLP) algorithms in routine healthcare records to identify reported somatic passivity and thought interference symptoms (thought broadcasting, insertion, withdrawal), and determine associations with prognosis by analysing routine outcomes.

Methods: Four algorithms were developed on de-identified mental healthcare data from a large south London provider and applied to ascertain recorded symptoms over the three months following first presentation to that service in a cohort of patients with a primary schizophreniform disorder (ICD-10 F20-F29) diagnosis. The primary binary dependent variable for logistic regression analyses was any negative outcome (Mental Health Act section, >2 antipsychotics prescribed, >22 days spent in crisis care) over the subsequent 2 years, adjusted for age, gender, ethnic group, neighbourhood deprivation, diagnostic group, and recorded paranoia, persecutory delusions or auditory hallucinations.

Outcomes: In 9,323 patients, final models indicated significant associations of this composite outcome with baseline somatic passivity (prevalence 4.9%; adjusted odds ratio 1.61, 95% CI 1.37-1.88), thought insertion (10.7%; 1.24, 1.15-1.55) and thought withdrawal (4.9%; 1.36, 1.10-1.69), but not independently with thought broadcast (10.3%; 1.05, 0.91-1.22).

Conclusions: Symptoms traditionally central to the diagnosis of schizophrenia, but under-represented in current diagnostic frameworks, were thus identified as important predictors of short- to medium-term prognosis.

Keywords: Schizophrenia; Delusions; Prognosis; Psychosis; Patient Outcomes; Natural-Language Processing (NLP)

Strengths and limitations

- This study was carried out on a very large patient sample and identified symptoms from routine care instead of specifically recruited and therefore less representative cohorts, which alongside the high-performance metrics of our application ensured better reliability.
- The patient sample was drawn from an inner urban area in the South of London, so results may not be entirely generalisable to the rest of the population.
- The recording of exposure variables relied on symptoms being elicited and noted by clinicians in routine practice and therefore cannot be assumed to be equivalent to those ascertained in a more research-focused interview, just as covariates such as diagnosis also were derived from routine clinical records and should not be assumed to equate research diagnostic criteria; however, we feel that inaccuracies in exposure ascertainment were more likely to obscure findings of interest through non-differential measurement error rather than give rise to spurious associations.
- Although multiple confounding factors were accounted for, our model-fit measures demonstrate model reliability but highlight residual unexplained variance possibly due to the influence of other psychotic symptoms as well as additional external factors which may influence outcomes which should be further investigated.
- Outcomes analysed were restricted to those most readily available from the source data and most likely to be clinically informative; however, they cannot be viewed as exhaustive and there may be elements of prognosis that were not captured in this study, including interactions between outcomes and previous adverse events that may influence symptom occurrence and recording.

For peer review only - http://bmjopen.bmj.com/site/about/guidelines.xhtml

INTRODUCTION

Since Schneider's definition of schizophrenia, diagnostic guidelines have mostly revolved around first-rank symptoms (FRS), including auditory hallucinations, and delusions of perception or thought interference, considered to carry strong diagnostic value. Recently, a more holistic approach accounting for a wider variety of symptom profiles has been promoted, culminating in the removal of FRS from DSM-5 diagnostic criteria[1-2]. There are likely to be complex mechanisms underpinning psychosis manifestations and phenomenology. First, different symptoms have been observed to occur in clusters, e.g. somatic passivity and either persecutory delusions[3] or auditory verbal hallucinations[4]. Second, environmental factors seem to influence symptom occurrence, and the cross-cultural relevance of FRS has been questioned[5]. Third, schizophrenia symptoms can directly influence both behaviour and consequently patient outcomes; for example, indirectly leading to violent behaviour through the occurrence of delusions[6]. FRS absence has been found to correlate with better outcomes, leading to a proposed redefinition of schizophrenia as a continuum, exhibiting a positive linear relationship between psychotic symptoms and outcomes[7-9]. Furthermore, the prevalence of individual symptoms appears to vary between early- and late-onset cases, reflecting complex interactions between symptoms and disease development[10-12]. These findings suggest that patient outcomes are better in schizophrenia in the absence of FRS[8] but offer no insight into prognosis once present or into relationships between symptoms.

In agreement with these findings, recent DSM-5 changes have been welcomed as a valuable push towards a better understanding of individual symptoms; however, the same authors explain that limitations remain as this new focus does not satisfyingly address the relationship between symptoms, conflicts with most existing guidelines for early symptom detection in

For peer review only - http://bmjopen.bmj.com/site/about/guidelines.xhtml

schizophrenia that heavily rely on FRS and diverts attention away from core psychopathological processes at the disorder's root. [13]. Recent studies have consistently found thought interference (the specific delusion that thoughts are being inserted in, withdrawn or broadcast from one's head) and somatic passivity (the broader delusion that one's actions and sensations are controlled by an external force) to be core symptoms of psychotic disorders in general and schizophrenia specifically[14-16]. A comparative study of thirteen different diagnostic systems identified a triad of positive symptoms strongly correlated with a schizophrenia diagnosis across all scales, the presence of two of which was sufficient to diagnose 78% of patients; these were auditory hallucinations, disturbances of affect and "passivity feelings" (defined as "any delusion of being influenced or interfered with by imaginary forces from outside, whether somatically or mentally" and therefore assumed to include thought interference)[17]; these symptoms were all already described in Bleuler's 1911 definition of schizophrenia[18]. Somatic passivity and thought interference were also reported to be positively correlated and predict the occurrence of schizophrenia or schizoaffective disorder[19-20]. While some authors have identified differing effects on outcomes, with passivity predicting a worsened prognosis in contrast to interference[8] , others have highlighted a possible link, as both phenomenologically reflect a belief in external control, suggesting they may have a synergistic effect on outcomes[20-22] or constitute components of the same delusion[4&23].

Considering this evidence, clarifying the broader role of FRS in prognosis is crucial to understand which aspects of schizophrenia should be at the core of its diagnosis. With this in mind, this study more specifically aimed to develop four natural language processing (NLP) algorithms to detect the recording of thought interference subcomponents (broadcasting, insertion, withdrawal) and somatic passivity in text from a large corpus of de-identified mental health records to investigate recorded prevalence, associations with other important positive

symptoms of psychosis (paranoia, auditory hallucinations, persecutory delusions), and outcome associations in patients with schizophreniform disorders. Our focus on thought interference and somatic passivity as particular symptoms aimed to clarify the interactions described previously; in addition, they were chosen as potentially tractable to extraction at scale from routine healthcare data via NLP, being described in relatively consistent language in clinical records and thus a pragmatic choice for further investigation.

For peer review only - http://bmjopen.bmj.com/site/about/guidelines.xhtml

METHODS

Context

We used data extracted from the electronic mental health records of patients at the South London and Maudsley NHS Foundation Trust (SLaM), one of the largest mental health service providers in Europe, serving a geographic catchment of four south London boroughs (Southwark, Lambeth, Lewisham, Croydon) with around 1.3 million residents. SLaM's Clinical Record Interactive Search (CRIS) platform was developed in 2008 to enable research on a de-identified copy of the Trust's electronic health records (used across all services since 2006) within a robust governance and data security framework[23]. CRIS has been described in detail previously[25-26] and supported over 200 research publications to date. Ethical approval as a data resource for secondary analysis in in place (Oxford Research Ethics Committee C, reference 18/SC/0372).

Patient sample

All patients aged 16-95 with a primary F20-F29 disorder diagnosis according to ICD-10 criteria recorded from 2007 to extraction date (25/02/2020) were included. 9,536 patients were identified. First diagnosis was used when several were recorded. Thought interference and somatic passivity occurrence in the three months following first presentation at SLaM (defined as first face-to-face contact) was sought. Participants who died within this period were excluded. Of the initial 9,536 patients, 9,323 (97.8%) were included, 57.9% of whom were male. Mean age at inclusion was 39.9 years (SD=17.3).

For peer review only - http://bmjopen.bmj.com/site/about/guidelines.xhtml

Exposure variables and software development

CRIS data have been enhanced over the last 10 years by a range of NLP algorithms allowing a hitherto unavailable level of detail to be generated at scale from routine healthcare data. The algorithms, over 90 to date, are described individually in an open-access online catalogue[27], including detailed information on definitions and performance. A particular objective has been to provide, at scale, broader phenotypic information on people presenting with mental disorders than supplied by clinician-assigned diagnostic codes through the extraction of recorded symptoms with 60 algorithms to date developed to ascertain individual symptoms across psychotic and affective disorders[28]. Contributing to this wider initiative, and with the specific rationale in mind for this study, we developed four new algorithms to identify instances of recorded somatic passivity and thought insertion, withdrawal and broadcasting in CRIS using the TextHunter platform[29]. TextHunter allows a training dataset of text strings containing wording of interest to be presented in an easy visual form for researcher annotation. The development process is identical for all algorithms relating to symptoms recorded in clinical text: 1) keyword search of the clinical record and annotation of retrieved documents based on whether the symptom was affirmed (patient is experiencing the symptom or is known to have experienced it), negated, or not relevant (e.g. the symptom described as being experienced by someone other than the patient, or a different use of the word/phrase in question); 2) division of annotated documents into gold standard and testing sets to generate the algorithm; 3) use of precision (P; positive predictive value) and recall (R; specificity) to assess the performance of the algorithm during development; 4) testing the performance of the algorithm over the entire CRIS database by checking 100 random annotated documents produced by the algorithm and 100 random unannotated documents using keyword searches. A second researcher double-

For peer review only - http://bmjopen.bmj.com/site/about/guidelines.xhtml

annotated a proportion of the retrieved documents to determine inter-rater reliability (IRR) using Cohen's kappa. The process was iterative and sought to achieve P and R values over 80%. High performance metrics were attained (supplementary table 1).

Outcomes

Occurrence of negative outcomes was recorded over the two years following first presentation at SLaM. Three outcomes were evaluated: i) highest-quartile time spent as an inpatient or receiving support from a home treatment team in the community since first presentation (defined as 22 days or more); ii) receiving more than two antipsychotics; iii) involuntary psychiatric hospitalisation defined as detention for assessment or treatment under Sections 2 or 3 of the UK Mental Health Act (MHA).

Covariates

Included covariates were: gender, ethnic group, age at first SLaM presentation, diagnosis, Index of Multiple Deprivation (IMD) score and presence of recorded paranoia, persecutory delusions or auditory hallucinations. Age was categorised by decades for descriptive analyses but applied as a continuous variable otherwise. Diagnoses were recorded as a binary variable, distinguishing schizophrenia (ICD-10 F20x) from other schizophreniform disorders (F21-F29)[30]. The IMD score is a measure of neighbourhood deprivation, applied at Lower Super Output Area level (a standard national address code containing an average 1500 residents), and derived from composite national Census measures. This was divided in quartiles ranging from low (1) to high (4) deprivation. The presence of paranoia, auditory hallucinations, and

For peer review only - http://bmjopen.bmj.com/site/about/guidelines.xhtml

persecutory delusions, three common psychotic symptoms in schizophrenia either previously suggested to strongly interact with passivity[19,31] or in the case of paranoia to constitute a syndrome distinct from that characterised by the presence of hallucinations or other delusions[23], was also ascertained for adjustment, using previously developed NLP algorithms[24].

Statistical analysis

All analyses were carried out using Stata 15.1 software. Initial descriptive analyses assessed overlap between symptoms, demographic/clinical factors and other psychotic symptoms. After describing the prevalence of outcomes by covariate, logistic regression models were assembled to investigate associations and their independence in relation to each binary outcome. Adjusted R-squared was used to assess model fit. Secondary analyses studied the impact of adjusting for covariates individually to clarify potential confounding effects. Finally, Poisson regression models investigated the relationship between number of negative outcomes and number of symptoms experienced as well as between number of negative outcomes and each individual symptom. Additional linear regression was carried out to study the relationship between symptoms and number of antipsychotics prescribed, assessing the reliability of the latter as a marker of negative outcome.

Patient and public involvement

The study proposal was reviewed and approved by the patient-led CRIS oversight committee prior to project commencement. No other consultations were made with patients or the public.

For peer review only - http://bmjopen.bmj.com/site/about/guidelines.xhtml

RESULTS

Table 1 describes symptom prevalences by demographic and clinical groups. Thought interference and somatic passivity were more common in younger patients and marginally higher in men compared to women but showed no consistent associations with ethnicity or neighbourhood deprivation. Both symptoms occurred more often in the presence of other psychotic symptoms but did not differ substantially between comparison diagnostic groups. Overlap between subcomponents of thought interference is summarised in Supplementary Figure 1. Supplementary Figure 2 describes overlap between passivity, thought interference and positive symptoms included as covariates.

Table 1: symptom prevalence by demographic characteristics and diagnoses, and overlap with other psychotic symptoms

		None (%)	Thought interference only (%)	Broadcast (%)	Insertion (%)	Withdrawal (%)	Somatic passivity only (%)	Interference and passivity (%)	Total
	Total participants	77.0	12.8	10.3	10.7	4.9	4.9	5.2	9,323
Age at diagnosis	16-25	65.8	19.3	16.1	17.4	7.8	6.3	8.5	2,131
	26-35	72.4	14.8	12.7	12.5	6.0	5.9	6.9	2,457
	36-45	80.2	11.3	8.6	8.7	4.4	4.3	4.2	1,799
	46-55	82.8	10.2	6.9	8.1	3.6	3.8	3.2	1,241
	56-65	87.6	6.9	4.1	4.8	2.0	3.8	1.7	651
	66-75	91.0	4.8	3.0	4.0	0.5	2.6	1.6	568
	76-85	93.5	3.4	2.6	1.6	1.0	2.9	0.3	383
	86-95	92.5	6.5	5.4	2.2	0.0	1.1	0.0	93
Gender	Female	80.3	11.2	8.5	10.2	3.8	4.4	4.2	3,920
	Male	74.7	14.1	11.5	11.1	5.7	5.3	6.0	5,399
Ethnicity	Black	74.9	14.1	11.2	11.7	5.5	5.4	5.7	3,065
	South Asian	76.1	16.3	12.0	11.6	7.3	2.7	5.0	301
	Chinese	72.4	9.2	10.5	7.9	2.6	10.5	7.9	76
	Other Asian	72.7	13.3	13.7	12.3	6.8	3.8	10.2	293
	White	78.3	12.4	10.1	10.3	4.4	4.6	4.8	3,631
	Other/unknown	79.0	11.4	8.3	9.6	4.3	5.0	4.6	1,957
IMD	IMD quartile 1	78.1	12.7	10.5	9.9	4.7	4.4	4.8	2,331
	IMD quartile 2	77.5	12.1	9.7	10.3	4.5	5.1	5.3	2,331
	IMD quartile 3	75.7	13.1	10.5	11.7	5.2	5.3	5.8	2,338
	IMD quartile 4	76.8	13.4	10.3	10.8	5.4	4.8	5.0	2,323
Diagnosis	Schizophrenia diagnosis (F20)	78.2	12.0	9.8	9.5	4.7	4.9	4.9	3,475
	Other diagnosis	75.3	13.4	10.5	11.4	5.0	4.9	6.5	5,848
Other psychotic symptom	Paranoia present	73.2	15.0	12.2	12.5	5.7	5.7	6.2	7,478
	Paranoia absent	92.7	4.5	2.5	3.5	1.8	1.7	1.1	1,845
	Auditory Hallucinations present	65.8	18.4	16.2	16.7	7.3	6.9	9.0	4,971
	Auditory Hallucinations absent	89.9	6.5	3.5	3.8	2.2	2.6	1.0	4,352
	Persecutory Delusions present	66.2	18.1	15.6	16.4	7.0	7.3	8.4	4,375
	Persecutory Delusions absent	86.0	8.2	5.6	5.6	3.1	2.8	2.4	4984

For peer review only - http://bmjopen.bmj.com/site/about/guidelines.xhtml

Using logistic regression, odds ratios (OR) were calculated to further study overlap between symptoms (Supplementary Table 2), reiterating increased prevalence of thought interference and somatic passivity when other symptoms are present. Thought insertion and broadcasting were strongly correlated (OR=11.67) as were insertion and withdrawal (OR=12.10), whereas there was a weaker association between broadcast and withdrawal (OR=6.05). Somatic passivity was relatively strongly associated with broadcast (OR=5.13) and insertion (OR=6.66) but was more strongly associated with other psychotic symptoms such as paranoia (OR=4.67) and auditory hallucinations (OR=4.99) than with thought withdrawal (OR=3.86).

Table 2 describes outcome distributions. Younger patients, patients from Black ethnic groups and patients from higher deprivation neighbourhoods were at greater risk of negative outcomes. Patients with schizophrenia were less likely to have an MHA section or higher time in crisis care than those with other diagnoses but more likely to be prescribed multiple antipsychotics. Negative outcomes were higher in patients with reported paranoia, auditory hallucinations or persecutory delusions, and in those with thought interference or somatic passivity. Supplementary Figure 3 describes overlap between outcomes.

For peer review only - http://bmjopen.bmj.com/site/about/guidelines.xhtml

Table 2: Two-year incidence of negative outcomes by symptom and covariate groups

	Covariate	Any negative Outcome (%)	MHA section (%)	>2 antipsychotics prescribed (%)	>22 days spent in crisis care (%)	Total participants
Interference/ Passivity	None	42.7	31.8	21.9	10.6	7,181
	Thought interference only	61.4	46.5	31.6	32.6	1,198
	Broadcast	*63.3*	*47.4*	*34.2*	*34.9*	*956*
	Insertion	*67.4*	*49.9*	*36.6*	*38.1*	*997*
	Withdrawal	*66.9*	*52.1*	*36.2*	*40.1*	*459*
	Somatic passivity only	68.2	49.8	36.4	35.7	456
	Interference and passivity	72.3	54.5	42.2	43.2	488
	Total participants	47.9	35.8	24.9	25.0	9,323
Age at diagnosis	16-25	61.0	48.6	32.7	36.8	2,131
	26-35	52.8	40.7	27.0	26.1	2,457
	36-45	55.3	32.9	22.5	21.2	1,799
	46-55	54.1	31.7	23.8	20.9	1,241
	56-65	33.5	21.8	17.8	16.9	651
	66-75	29.9	17.8	15.7	16.2	568
	76-85	24.3	15.4	11.7	13.3	383
	86-95	24.7	14.0	14.0	12.9	93
Gender	Female	46.9	35.2	25.1	24.2	3,920
	Male	48.6	36.2	24.8	25.6	5,399
Ethnicity	Black	57.5	47.6	30.0	31.3	3,065
	South Asian	43.5	30.2	24.9	19.3	301
	Chinese	50.0	44.7	15.8	21.1	76
	Other Asian	48.1	36.2	27.3	23.9	293
	White	43.9	31.1	23.3	22.1	3,631
	Other/unknown	40.7	26.4	20.0	21.7	1,957
IMD	IMD quartile 1	45.6	32.9	24.5	23.1	2,331
	IMD quartile 2	46.3	33.6	25.0	24.2	2,331
	IMD quartile 3	50.3	38.1	25.8	26.6	2,338
	IMD quartile 4	49.2	38.4	24.3	26.1	2,323
Diagnosis	Schizophrenia diagnosis (F20)	44.4	30.6	29.9	22.2	3,475
	Other diagnosis	49.9	38.8	22.0	26.7	5,848
Other symptoms	Paranoia present	53.9	41.0	27.7	28.9	7,478
	Paranoia absent	23.6	14.5	13.5	9.4	1,845
	Auditory Hallucinations present	58.2	43.8	31.3	31.7	4,971
	Auditory Hallucinations absent	36.0	26.6	17.6	17.3	4,352
	Persecutory Delusions present	62.2	49.2	31.9	34.4	4,375
	Persecutory Delusions absent	35.7	23.7	18.6	16.6	4984

For peer review only - http://bmjopen.bmj.com/site/about/guidelines.xhtml

Table 3 summarises logistic regression analyses for any negative outcome. Unadjusted models showed significant associations with somatic passivity and all components of thought interference, and these remained significant after adjustments except thought broadcast. Although confidence intervals overlapped, associations with somatic passivity were consistently strongest. Post-hoc investigations of those with non-overlapping somatic passivity and thought interference showed a significant association with somatic passivity (OR=1.57, 95% CI 1.27-1.95) but not with thought interference (OR=1.11, 95% CI 0.97-1.27).

For peer review only - http://bmjopen.bmj.com/site/about/guidelines.xhtml

Table 3: Unadjusted and adjusted odds ratio for the occurrence of any negative outcome in the presence of thought interference and somatic passivity

Outcome: any negative outcome	**Unadjusted OR (95% CI)**	**Adjusted* OR (95% CI)**	**Adjusted model pseudo R-squared**
Somatic passivity	2.86 (2.47-3.31)	1.61 (1.37-1.88)	0.12
Interference	2.30 (2.07-2.57)	1.25 (1.11-1.41)	0.12
Broadcast	2.01 (1.75-2.31)	1.05 (0.91-1.22)	0.12
Insertion	2.47 (2.15-2.84)	1.24 (1.15-1.55)	0.12
Withdrawal	2.29 (1.88-2.79)	1.36 (1.10-1.69)	0.12
Interference or passivity	2.54 (2.29-2.80)	1.30 (1.19-1.42)	0.12
Both interference and passivity	1.73 (1.57-1.92)	1.24 (1.11-1.38)	0.12
Post-hoc: passivity only	2.44 (1.99-2.98)	1.57 (1.27-1.95)	0.12
Post-hoc: interference only	1.88 (1.66-2.13)	1.11 (0.97-1.27)	0.12

*Adjusted for age at diagnosis, gender, ethnicity, diagnosis, IMD quartile and presence of paranoia, auditory hallucinations, persecutory delusion.
Abbreviations: OR = odds ratio; RR = risk ratio

For peer review only - http://bmjopen.bmj.com/site/about/guidelines.xhtml

Table 4 displays further analyses of individual outcomes. Associations with somatic passivity remained significant for all after adjustments. Thought interference and insertion specifically were independently associated with higher time spent in crisis care and greater numbers of antipsychotics used, but not with MHA sections. Thought broadcast specifically was not independently associated with any outcome, and thought withdrawal was only associated with higher time spent in crisis care and MHA sections. Aside from thought withdrawal, all other symptoms had the weakest association with MHA sections.

For peer review only - http://bmjopen.bmj.com/site/about/guidelines.xhtml

Table 4: Unadjusted and adjusted odds ratios for associations of each negative outcome with the presence of somatic passivity, thought interference, broadcasting, insertion and withdrawal

Exposure: somatic passivity	Unadjusted OR (95% CI)	Adjusted* OR (95% CI)	Adjusted model pseudo R-squared
Any negative outcome	2.86 (2.47-3.31)	1.61 (1.37-1.88)	0.12
>22 days spent in crisis care	2.15 (1.87-2.48)	1.33 (1.15-1.54)	0.08
MHA section	2.13 (1.86-2.44)	1.23 (1.06-1.42)	0.13
>2 antipsychotics prescribed	2.14 (1.86-2.46)	1.43 (1.23-1.66)	0.07
**Exposure: thought interference**			
Any negative outcome	2.30 (2.07-2.57)	1.25 (1.11-1.41)	0.12
>22 days spent in crisis care	1.89 (1.69-2.12)	1.13 (1.00-1.28)	0.08
MHA section	1.95 (1.75-2.17)	1.08 (0.96-1.21)	0.13
>2 antipsychotics prescribed	1.80 (1.60-2.01)	1.16 (1.02-1.31)	0.07
**Exposure: thought broadcasting**			
Any negative outcome	2.01 (1.75-2.31)	1.05 (0.91-1.22)	0.12
>22 days spent in crisis care	1.71 (1.49-1.97)	1.01 (0.87-1.18)	0.08
MHA section	1.71 (1.50-1.96)	0.92 (0.80-1.07)	0.13
>2 antipsychotics prescribed	1.66 (1.44-1.91)	1.05 (0.90-1.22)	0.07
**Exposure: thought insertion**			
Any negative outcome	2.47 (2.15-2.84)	1.34 (1.15-1.56)	0.12
>22 days spent in crisis care	2.01 (1.75-2.31)	1.21 (1.05-1.40)	0.08
MHA section	1.93 (1.69-2.20)	1.06 (0.92-1.23)	0.13
>2 antipsychotics prescribed	1.88 (1.64-2.16)	1.22 (1.05-1.41)	0.07
**Exposure: thought withdrawal**			
Any negative outcome	2.29 (1.88-2.79)	1.36 (1.10-1.69)	0.12
>22 days spent in crisis care	2.09 (1.73-2.54)	1.37 (1.12-1.68)	0.08
MHA section	2.02 (1.68-2.44)	1.23 (1.01-1.51)	0.13
>2 antipsychotics prescribed	1.76 (1.45-2.14)	1.21 (0.98-1.48)	0.07

*Adjusted for age at diagnosis, gender, ethnicity, diagnosis, IMD quartile and presence of paranoia, auditory hallucinations, persecutory delusion.
Abbreviations: OR = odds ratio; MHA = Mental Health act

Table 5 summarises analyses investigating adjustments by individual covariates. For both somatic passivity and thought interference, strongest reductions in OR occurred following adjustments for age and the other three psychotic symptoms; however, OR for somatic passivity remained stronger throughout. Estimated marginal means (EM) were calculated to determine the likelihood of negative outcomes at different ages (Supplementary Table 3); this showed a decrease in risk inversely proportional to age: for a patient with somatic passivity, at age 20 EM=59.6(58.0-61.1)%; at age 90, EM=21.5(19.2-23.8)%. For a patient with thought interference, at age 20 EM=59.1(57.6-60.7)%; at age 90, EM=22.2(19.8-24.6)%. Finally, exploratory testing investigating the cumulative influence of symptoms on outcomes, calculating the odds of experiencing multiple negative outcomes in relation to the sum of individual symptoms and to each individual symptom and illustrated using EM, described a 14.9% increase in the risk of multiple negative outcomes when experiencing no versus all four symptoms, from 73.1% to 88.0%. Consistently with identified trends, somatic passivity was the individual symptom with the greatest impact (adjusted OR=1.16(1.09-1.23)) and thought broadcasting had no significant effect after adjustment (OR=0.99(0.92-1.05)). Regression analysis identified a significant interaction between somatic passivity and number of antipsychotics ($p<0.05$; adjusted R-squared=0.1031). Thought interference and its subcomponents, also included in the model, had no significant effect (supplementary table 4).

For peer review only - http://bmjopen.bmj.com/site/about/guidelines.xhtml

Table 5: Logistic regression analysis of associations with any negative outcome adjusting separately for individual covariates

Outcome: Any negative outcome	**Somatic Passivity**	**Thought Interference**
Unadjusted	2.86 (2.47-3.31)	2.30 (2.07-2.57)
Adjusted for age at diagnosis	2.47 (2.13-2.86)	1.94 (1.73-2.17)
Adjusted for gender	2.85 (2.46-3.30)	2.30 (2.06-2.57)
Adjusted for ethnicity	2.85 (2.46-3.30)	2.28 (2.04-2.55)
Adjusted diagnosis	2.86 (2.47-3.31)	2.30 (2.06-2.56)
Adjusted for IMD quartile	2.86 (2.47-3.31)	2.30 (2.06-2.57)
Adjusted paranoia	2.43 (2.09-2.82)	1.94 (1.74-2.17)
Adjusted for auditory hallucinations	2.23 (1.92-2.59)	1.80 (1.61-2.02)
Adjusted for persecutory delusions	2.23 (1.91-2.59)	1.82 (1.63-2.04)

For peer review only - http://bmjopen.bmj.com/site/about/guidelines.xhtml

DISCUSSION

Having developed NLP algorithms to extract recorded thought interference and somatic passivity in routine electronic mental health records, we investigated their associations with negative outcomes over a 2-year period following first-recorded diagnosis of schizophrenia or a schizophreniform disorder. Both symptoms were associated with a significantly increased risk of experiencing negative outcomes, somatic passivity having the strongest associations after full adjustment. This is broadly consistent with previous reports that experiencing these symptoms is predictive of negative outcomes: Stephen et al found the absence of somatic passivity to correlate with better outcomes in 66% of patients[7]; Koehler found no significant difference in outcomes between patients experiencing any thought interference subcomponent or somatic passivity but concluded that their absence was predictive of a better prognosis[8]. In 2019, Malinowski et al found that thought broadcasting seemed predictive of disease continuation without remission, but that this effect was no longer significant after adjusting for external factor influence (specifically, untreated psychosis duration and drug abuse)[23]; accordingly, although different covariates were included, our results suggest that thought broadcast does not have a significant effect on outcome. Similar prevalence and overlap between thought insertion, withdrawal and broadcasting suggests that this cannot be due to broadcasting occurring more frequently in isolation. Secondary testing identified a unique significant effect of somatic passivity on number of antipsychotics prescribed, possibly reflecting poorer response leading to worsened outcomes; importantly, this outcome may reflect a degree of treatment resistance not investigated here but likely to influence patient prognosis. Our findings suggest that somatic passivity and thought interference subcomponents increase the risk of experiencing negative outcomes, some with a stronger effect than others. When considering the influence of individual symptoms on the odds of experiencing each

outcome, it is interesting to note that no particular outcome appeared to be disproportionately impacted: although the odds of having an MHA section tended to remain lower, as its use is reserved for the most unwell patients who refuse or lack capacity to accept voluntary community treatment, overall the odds of all three outcomes increased in a similar fashion in the presence of thought interference and somatic passivity, reflecting a generally worsened prognosis.

Nuevo et al described schizophrenia symptoms as a continuum whereby the experience of multiple symptoms predicts a worse prognosis[9]. Our finding that patients with either or both thought interference and somatic passivity were more likely to experience negative outcomes than those for whom these symptoms were absent supports this. Exploratory analyses suggested a significant relationship between number of symptoms experienced and negative outcome occurrence; however, our results also suggest that each symptom has a different impact on outcomes which was not always individually significant, and that the presence of multiple symptoms may even sometimes reduce the frequency of negative outcomes: for example, patients experiencing somatic passivity only may have poorer outcomes than those with both this and thought interference. One explanation may be that reporting thought interference to clinicians requires greater insight from the patient, contributing to better outcomes and facilitating voluntary treatment such as that offered by crisis care teams with which more patients experiencing thought insertion and withdrawal engaged. Overall, although there does seem to be a trend of worsening outcomes as symptoms accumulate, it would be overly simplistic to claim that this effect is purely additive. Interestingly, of the covariates investigated, only age had a substantial confounding effect, negatively correlated with symptoms and outcomes of interest. In the past, thought insertion and withdrawal have been reported as commoner in early-onset cases and somatic passivity as more frequent in late-onset

For peer review only - http://bmjopen.bmj.com/site/about/guidelines.xhtml

cases[10]; although age at diagnosis may not accurately reflect age at disease onset and doesn't account for possible changes in symptom profile as patients age, our results suggest that both symptoms are more frequent in younger patients, leading to a worsened prognosis. Other external factors such as level of education or spiritual beliefs may play a role in the personal interpretation of abnormal experiences and have consequently influenced outcomes. Our model-fit measures demonstrate model reliability but highlight that the variance remains in great part unexplained, likely due to the influence of other factors not included here. This should be explored in further studies to refine models assessing patient prognosis. Paranoia, persecutory delusions and auditory hallucinations were included due to past evidence suggesting a correlation with thought interference and somatic passivity, but other psychotic symptoms would be worth investigating in future research as well as interactions between outcomes; for example, it is not unreasonable to propose that the prescription of fewer antipsychotics may correlate with reduced hospitalisation time.

Although to be treated with caution as not tested for significance, our descriptive results provide potential directions for future research and support the notion that psychotic symptoms share more complex interactions than previously described. Somatic passivity has been suggested to cluster with different symptoms such as persecutory delusions[3] or auditory-verbal hallucinations[4], and thought interference has sometimes been described as a component of somatic passivity[17&33]. We found that subcomponents of thought interference traditionally assumed to closely correlate may actually more commonly occur alongside other symptoms; e.g., the odds of experiencing thought insertion alongside somatic passivity were greater than those of withdrawal and broadcasting co-occurring. In a study attempting to link delusions to putative underlying neurobiological mechanisms, Kimhy et al called for new symptom definitions that would focus on underlying mechanisms rather than phenomenological

descriptions[22]. Each delusion was described then grouped with others based on correlation, enabling the grouping of symptoms that share significant interactions while maintaining their individuality. This approach enables a less biased study of psychotic symptoms relying less on traditional groupings and may allow more accurate descriptions of symptom interactions. Additionally, thought interference and somatic passivity have previously been found to be closely related and predictive of schizophrenia[12&33-35]. Our results support reports that these symptoms share a positive correlation[19] but question their specificity to schizophrenia and usefulness in differentiating this from other psychotic disorders, particularly as both were absent in 77% of patients. Somatic passivity has been reported to be the most common symptom experienced in schizophrenia[5] and first-episode psychosis[23]; however, in our study thought interference was almost twice as commonly recorded.

CONCLUSION

This study developed NLP applications to detect the recorded presence of somatic passivity, thought interference and its subcomponents in patients within three months of their first presentation and analysed negative outcome occurrence over the two following years to investigate the influence of these specific FRS on patient prognosis. We observed a significant effect of somatic passivity, thought interference and its subcomponents thought insertion and withdrawal on the likelihood of experiencing any negative outcome. We also offer evidence that psychotic symptoms traditionally associated with schizophrenia may not be specific to this disorder and share complex interactions, putting in question current definitions and warranting further investigation to determine whether it is correct to consider thought insertion, withdrawal and broadcasting as parts of the same symptom. Overall, the presence of somatic

For peer review only - http://bmjopen.bmj.com/site/about/guidelines.xhtml

passivity and thought interference was found to significantly increase the risk of experiencing negative outcomes in psychosis; somatic passivity had the greatest effect. Future research should focus on acquiring a better understanding of these effects, including by investigating other psychotic symptoms to determine the more detailed pathways through which these influence prognosis in schizophrenia and other types of psychosis. Future studies should investigate these correlations and reliable markers of outcomes to develop standardised tools enabling a better evaluation of patient prognosis. In addition, it would be interesting to study which antipsychotics specifically were prescribed and whether patients may have had a better response to specific medication, reflective of a general lack of response to individual antipsychotics in schizophrenia rather than the presence of refractory disease.

For peer review only - http://bmjopen.bmj.com/site/about/guidelines.xhtml

Funding

This research received no specific grant from any funding agency in the public, commercial or not-for-profit sectors.

Conflict of Interest

The authors have no conflict of interest to declare. RS declares research support received in the last 36 months from Janssen, GSK and Takeda. RP has received grant funds from Janssen and consultancy fees from Induction Healthcare and Holmusk. RS, RP and AK are part-funded by the National Institute for Health Research (NIHR) Biomedical Research Centre at the South London and Maudsley NHS Foundation Trust and King's College London. RS is additionally part-funded by: i) a Medical Research Council (MRC) Mental Health Data Pathfinder Award to King's College London; ii) an NIHR Senior Investigator Award; iii) the National Institute for Health Research (NIHR) Applied Research Collaboration South London (NIHR ARC South London) at King's College Hospital NHS Foundation Trust. The views expressed are those of the authors and not necessarily those of the NIHR or the Department of Health and Social Care. RP has received support from a Medical Research Council (MRC) Health Data Research UK Fellowship (MR/S003118/1) and a Starter Grant for Clinical Lecturers (SGL015/1020) supported by the Academy of Medical Sciences, The Wellcome Trust, MRC, British Heart Foundation, Arthritis Research UK, the Royal College of Physicians and Diabetes UK.

Author Statement

TM, AK, JS, RP and RS planned the study together. JS developed the applications, which were doubly annotated by TM and AK. JS and AK retrieved the data, and TM analysed it with advice from AK, JS, RP and RS. TM wrote the largest part of the paper, produced the tables and figures and submitted it, working under supervision from AK and RS. AK, RP and RS commented on and directly contributed to the manuscript.

Ethics Statement

This study was approved by Oxford Research Ethics Committee C, reference 18/SC/0372.

Data Sharing

No additional data are available.

REFERENCES

1. American Psychiatric Association. *Diagnostic and Statistical Manual of Mental Disorders, Fifth Edition (DSM-5)*. Washington, DC: American Psychiatric Association; 2013.
2. Kendler KS. Phenomenology of Schizophrenia and the Representativeness of Modern Diagnostic Criteria. *Jama Psychiat*. 2016;73(10):1082 doi:10.1001/jamapsychiatry.2016.1976 [published Online First: October 2016]
3. Fierro M, Hernández AM, Malcolm J. The Psychopathology of Delusion of Control According to Subjective Experience. *Revista Colombiana De Psiquiatría*. 2018;47(4):221-228 doi:10.1016/j.rcpeng.2018.10.005 [published Online First: December 2018]
4. Rosen C, Jones N, Chase KA, et al. The intrasubjectivity of self, voices and delusions: A phenomenological analysis. *Psychos*. 2016;8(4):357-368 doi: 10.1080/17522439.2016.1162839 [published Online First: April 2016]
5. Malik SB, Ahmed M, Bashir A, et al. Schneider's First-Rank Symptoms of Schizophrenia: Prevalence and Diagnostic Use. *Brit J Psychiat*. 1990;156(1):109-111 doi: 10.1192/bjp.156.1.109 [published in print First: January 1990]
6. Ullrich S, Keers R, Coid JW. Delusions, anger, and serious violence: new findings from the MacArthur Violence Risk Assessment Study. *Schizophrenia Bull*. 2013;40(5):1174-1181 doi: doi.org/10.1093/schbul/sbt126 [published Online First: September 2013]
7. Stephens JH, Richard P, McHugh PR. Long-Term Follow-up of Patients Hospitalized for Schizophrenia, 1913 to 1940. *J Nerv Amp Ment Dis*. 1997;185(12):715-721.
8. Koehler K. Prognostic Prediction in RDC Schizo-Affective Disorder on the Basis of First-Rank Symptoms Weighted in Terms of Outcome. *Psychopathology*. 1983;16(2-4):186-197 doi: 10.1159/000283967 [published in print First: 1983]

For peer review only - http://bmjopen.bmj.com/site/about/guidelines.xhtml

9. Nuevo R, Chatterji S, Verdes E, et al. The continuum of psychotic symptoms in the general population: a cross-national study. *Schizophrenia Bull*. 2010;38(3):475-485 doi: 10.1093/schbul/sbq099 [published Online First: September 2010]

10. Howard R, Castle D, Wessely S, et al. A Comparative Study of 470 Cases of Early-Onset and Late-Onset Schizophrenia. *Brit J Psychiat*. 1993;163(3):352-357 doi: 10.1192/bjp.163.3.352 [published in print First: September 1983]

11. Alici-Evcimen Y, Ertan T, Eker E. Case Series With Late-Onset Psychosis Hospitalized in a Geriatric Psychiatry Unit in Turkey: Experience in 9 Years. *Int Psychogeriatr*. 2003;15(1):69-72 doi: 10.1017/S1041610203008767 [published Online First: March 2003]

12. Naqvi HA, Hussain S, Islam M, et al. Early psychosis symptoms. *J Coll Physicians Surg Jcpsp*. 2014;24(3):198-202.

13. Nemeroff CB, Weinberger D, Rutter M, et al. DSM-5: a collection of psychiatrist views on the changes, controversies, and future directions. *Bmc Med*. 2013;11(1):202 doi: 10.1186/1741-7015-11-202 [published Online First: September 2013]

14. Chapman LJ, Chapman JP. Scales for Rating Psychotic and Psychotic-like Experiences as Continua. *Schizophrenia Bull*. 1980;6(3):476-489 doi: 10.1093/schbul/6.3.476 [published in print First: January 1980]

15. Chapman LJ, Edell WS, Chapman JP. Physical Anhedonia, Perceptual Aberration, and Psychosis Proneness. *Schizophrenia Bull*. 1980;6(4):639-653 doi: 10.1093/schbul/6.3.476 [published in print First: January 1980]

16. Kwapil TR, Chapman LJ, Chapman J. Validity and Usefulness of the Wisconsin Manual for Assessing Psychotic-like Experiences. *Schizophrenia Bull*. 1999;25(2):363-375 doi: 10.1093/oxfordjournals.schbul.a033384 [published in print First: 1999]

For peer review only - http://bmjopen.bmj.com/site/about/guidelines.xhtml

17. Landmark J, Merskey H, Cernovsky Z, et al. The positive triad of schizophrenic symptoms. Its statistical properties and its relationship to 13 traditional diagnostic systems. *Br J Psychiatry J Ment Sci*. 1990;156(3):388-394. doi: 10.1192/bjp.156.3.388 [published Online First: July 2011]

18. Bleuler, E. *Dementia Praecox oder Gruppe der Schizophrenien*. Leipzig: Germany Deuticke; 1911.

19. Cloninger CR, Martin RL, Guze SB, et al. Diagnosis and Prognosis in Schizophrenia. *Arch Gen Psychiat*. 1985;42(1):15 doi: 10.1001/archpsyc.1985.01790240017002 [published in print First: January 1985]

20. Loftus J, Delisi LE, Crow TJ. Factor structure and familiality of first-rank symptoms in sibling pairs with schizophrenia and schizoaffective disorder. *Brit J Psychiat*. 2000;177(1):15-19 doi: 10.1192/bjp.177.1.15 [published Online First: July 2000]

21. Stanghellini G, Monti MR. Influencing and being influenced: the other side of "bizarre delusions". 2. Clinical investigation. *Psychopathology*. 1993;26(3-4):165-169 doi: 10.1159/000284817 [published in print First: February 1993]

22. Kimhy D, Goetz R, Yale S, et al. Delusions in Individuals with Schizophrenia: Factor Structure, Clinical Correlates, and Putative Neurobiology. *Psychopathology*. 2005;38(6):338-344 doi: 10.1159/000089455 [published Online First: December 2005]

23. Fernandes AC, Cloete D, Broadbent MTM, et al. Development and evaluation of a de-identification procedure for a case register sourced from mental health electronic records. *Bmc Med Inform Decis*. 2013;13(1):71 doi: 10.1186/1472-6947-13-71 [published Online First: July 2013]

24. Stuart GW, Malone V, Currie J, et al. Positive and negative symptoms in neuroleptic-free psychotic inpatients. *Schizophr Res*. 1995;16(3):175-188 doi: 10.1016/0920-9964(94)00083-K [published in print First: August 1995]

For peer review only - http://bmjopen.bmj.com/site/about/guidelines.xhtml

25. Stewart R, Soremekun M, Perera G, et al. The South London and Maudsley NHS Foundation Trust Biomedical Research Centre (SLAM BRC) case register: development and descriptive data. *Bmc Psychiatry*. 2009;9(1):51 doi: 10.1186/1471-244X-9-51 [published Online First: August 2009]

26. Perera G, Broadbent M, Callard F, et al. Cohort profile of the South London and Maudsley NHS Foundation Trust Biomedical Research Centre (SLaM BRC) Case Register: current status and recent enhancement of an Electronic Mental Health Record-derived data resource. *Bmj Open*. 2016;6(3):e008721 doi: 10.1136/bmjopen-2015-008721 [published Online First: March 2016]

27. CRIS NLP Service. CRIS Natural Language Processing Applications Library (2020), v1.2. Maudsley Biomedical Research Centre. https://www.maudsleybrc.nihr.ac.uk/facilities/clinical-record-interactive-search-cris/cris-natural-language-processing/. Updated 12/06/20. Accessed 03/08/20.

28. Jackson RG, Patel R, Jayatilleke N, et al. Natural language processing to extract symptoms of severe mental illness from clinical text: the Clinical Record Interactive Search Comprehensive Data Extraction (CRIS-CODE) project. *Bmj Open*. 2017;7(1):e012012 doi: 10.1136/bmjopen-2016-012012 [published Online First: January 2017]

29. Jackson RG, Ball M, Patel R, et al. TextHunter--A User Friendly Tool for Extracting Generic Concepts from Free Text in Clinical Research. *Amia Annu Symposium Proc Amia Symposium Amia Symposium*. 2014;2014:729-738 doi: 10.13140/2.1.3722.9121 [published Online First: November 2014]

30. World Health Organisation. *International Statistical Classification of Diseases and Related Health Problems, 10th Revision (ICD-10)*. Geneva: WHO; 1992.

For peer review only - http://bmjopen.bmj.com/site/about/guidelines.xhtml

31. Galdos P, Os J van. Gender, psychopathology, and development: from puberty to early adulthood. *Schizophr Res*. 1995;14(2):105-112 doi: 10.1016/0920-9964(94)00020-9 [published in print First: January 1995]

32. Malinowski FR, Tasso B de C, Ortiz BB, et al. Schneider's first-rank symptoms as predictors of remission in antipsychotic-naive first-episode psychosis. *Revista Brasileira De Psiquiatria Sao Paulo Braz 1999*. 2019;42(AHEAD):22-26 doi: 10.1590/1516-4446-2018-0237 [published Online First: February 2020]

33. Klosterkötter J, Hellmich M, Steinmeyer EM, et al. Diagnosing Schizophrenia in the Initial Prodromal Phase. *Arch Gen Psychiat*. 2001;58(2):158 doi: 10.1001/archpsyc.58.2.158 [published Online First: February 2001]

34. Moller P, Husby R. The Initial Prodrome in Schizophrenia: Searching for Naturalistic Core Dimensions of Experience and Behavior. *Schizophrenia Bull*. 2000;26(1):217-232 doi: 10.1093/oxfordjournals.schbul.a033442 [published Online First: January 2000]

35. Galdos P, Os J van. Gender, psychopathology, and development: from puberty to early adulthood. *Schizophr Res*. 1995;14(2):105-112 doi: 10.1016/0920-9964(94)00020-9 [published in print First: January 1995]

For peer review only - http://bmjopen.bmj.com/site/about/guidelines.xhtml

Supplementary Material

Supplementary Table 1: Natural language processing algorithm development and performance. A minimum P and R of 80% is required for an algorithm to be considered reliable. In the context of passivity, the lower IRR led to the inclusion of extra annotations that enabled us to reach this threshold.

Symptom	**Keyword**	**Number of annotations**	**IRR**	**P and R**	**Extra steps required**	**Manual testing**	**Keyword search P and R**
Thought insertion	"thought insert*"	592 (96 double-annoted)	97 %	P=81%; R=94%	N/A	P=84%	P=81%; R=96%
Thought broadcasting	"thought broadcast*"	398 (95 double-annoted)	94 %	P=81%; R=94%	N/A	P=84%	P=81%; R=96%
Thought withdrawal	"thought withdraw*"	500 (76 double-annotated)	95 %	P=74%; R=91%	159 extra annotations	P=84%	P= 90%; R=88%
Somatic passivity	"passivity"	500 (116 double-annotated)	71 %	P=72%; R=92%	597 extra annotations, form excluded	P=82%	P=89%; R=100%

Supplementary Table 2: Unadjusted odds ratios for the co-occurrence of psychotic symptoms across the patient sample

Symptom	Broadcast	Insertion	Withdrawal	Passivity	Paranoia	Auditory Hallucin ations	Persecutory Delusions
Broadcast	1						
Insertion	11.67	1					
Withdrawal	6.05	12.10	1				
Passivity	5.13	6.66	3.86	1			
Paranoia	5.29	3.97	3.32	4.67	1		
Auditory Hallucinations	5.33	5.06	3.45	4.99	3.50	1	
Persecutory Delusions	3.13	3.30	2.41	3.40	5.30	2.80	1

For peer review only - http://bmjopen.bmj.com/site/about/guidelines.xhtml

Supplementary Table 3: Prevalence of negative outcomes in patients with somatic passivity or thought interference at different ages

Age (years)	Occurrence on negative outcomes in patients with somatic passivity (%) (95% CI)	Occurrence on negative outcomes in patients with thought interference (%) (95% CI)
20	59.6 (58.0-61.1)	59.1 (57.6-60.7)
30	53.7 (52.5-54.9)	53.4 (52.2-54.6)
40	47.7 (46.6-48.7)	47.7 (46.6-48.7)
50	41.7 (40.5-43.0)	41.9 (40.7-43.2)
60	36.1 (34.5-37.6)	36.4 (34.8-38.0)
70	30.7 (28.8-32.6)	31.2 (29.3-33.2)
80	25.9 (23.7-28.0)	26.5 (24.3-28.7)
90	21.5 (19.2-23.8)	22.2 (19.8-24.6)

For peer review only - http://bmjopen.bmj.com/site/about/guidelines.xhtml

Supplementary Table 4: Linear regression describing the influence of somatic passivity, thought interference and its subcomponents on the number of antipsychotics prescribed

Symptom	Standardized Beta Coefficients (95% CI)	t	Significance
Somatic Passivity	0.35 (0.24-0.45)	6.43	0.00
Thought Interference	0.40 (-0.14-0.22)	0.43	0.67
Thought Insertion	0.14 (0-0.01-0.30)	1.81	0.07
Thought Withdrawal	-0.00 (-0.17-0.16)	-0.05	0.96
Thought Broadcasting	-0.02 (-0.18-0.13)	-0.29	0.77

Adjusted for age at diagnosis, gender, ethnicity, diagnosis, IMD quartile and presence of paranoia, auditory hallucinations, persecutory delusion.

For peer review only - http://bmjopen.bmj.com/site/about/guidelines.xhtml

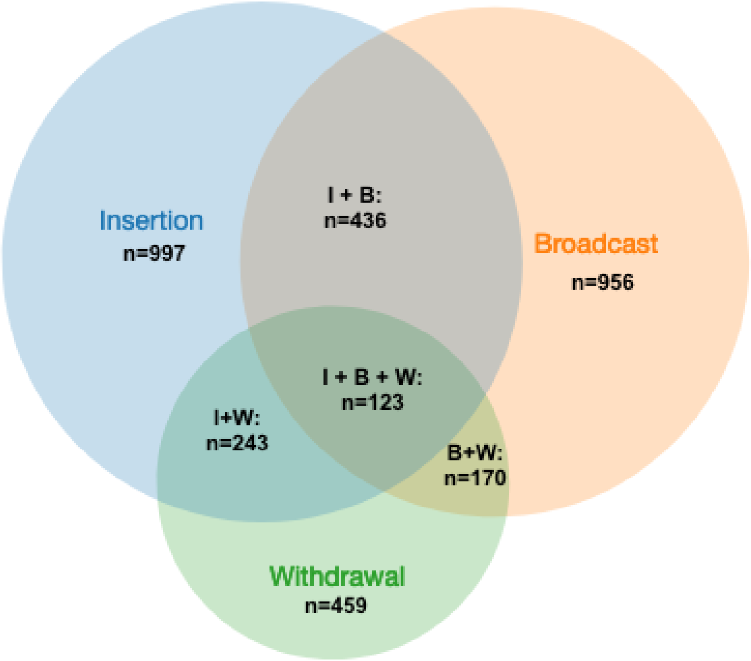

Supplementary Figure 1: Venn diagram describing the overlap between thought broadcasting, thought insertion and thought withdrawal

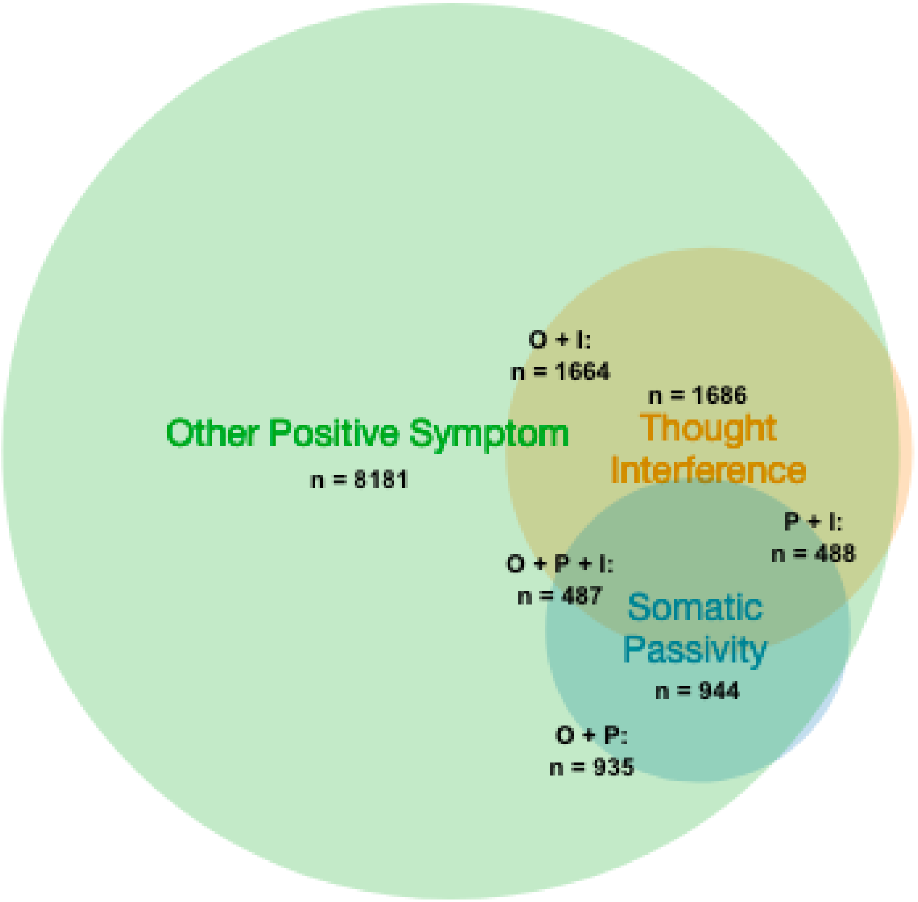

Supplementary figure 2: Venn diagram describing the overlap between somatic passivity, thought interference and any other positive symptoms (auditory hallucinations, persecutory delusions, paranoia) . I: Thought Interference; P: Passivity; O: Other Positive Symptom. When carrying out analyses, other positive symptoms were not combined but each included as individual covariates.

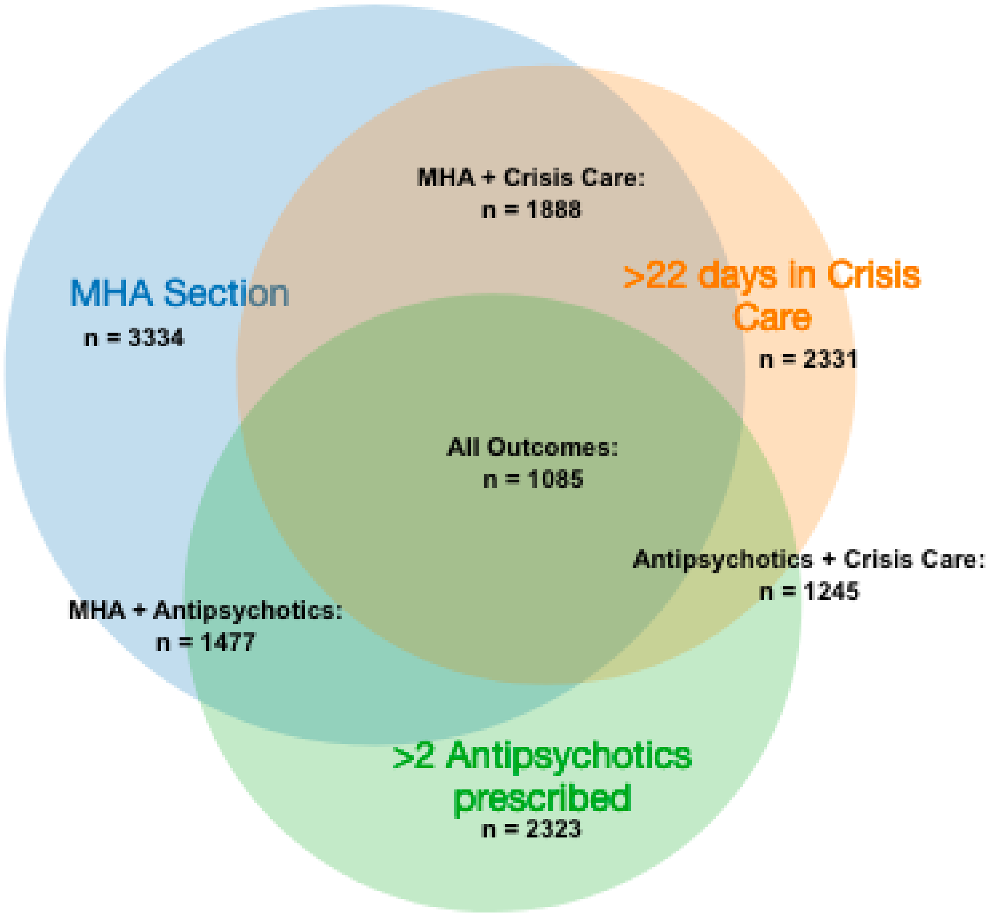

Supplementary Figure 3: Venn diagram describing the overlap between Outcomes investigated. MHA: presence of a Mental Health act section.

For peer review only - http://bmjopen.bmj.com/site/about/guidelines.xhtml

The RECORD statement – checklist of items, extended from the STROBE statement, that should be reported in observational studies using routinely collected health data.

	Item No.	STROBE items	Location in manuscript where items are reported	RECORD items	Location in manuscript where items are reported
**Title and abstract**					
	1	(a) Indicate the study's design with a commonly used term in the title or the abstract (b) Provide in the abstract an informative and balanced summary of what was done and what was found	Title page and abstract (p1&2)	RECORD 1.1: The type of data used should be specified in the title or abstract. When possible, the name of the databases used should be included.<br><br>RECORD 1.2: If applicable, the geographic region and timeframe within which the study took place should be reported in the title or abstract.<br><br>RECORD 1.3: If linkage between databases was conducted for the study, this should be clearly stated in the title or abstract.	Title page (p1) and abstract (p2)<br><br>Abstract (p2)<br><br>N/A
**Introduction**					
Background rationale	2	Explain the scientific background and rationale for the investigation being reported	Introduction (p3-5)		
Objectives	3	State specific objectives, including any prespecified hypotheses	Abstract (p2) and Introduction (p4&5, final paragraph)		
**Methods**					
Study Design	4	Present key elements of study design early in the paper	Methods (p6-9)		
Setting	5	Describe the setting, locations, and relevant dates, including periods of recruitment, exposure, follow-up, and data collection	Methods, (p6-9)		

For peer review only

Participants	6	*(a) Cohort study* - Give the eligibility criteria, and the sources and methods of selection of participants. Describe methods of follow-up<br>*Case-control study* - Give the eligibility criteria, and the sources and methods of case ascertainment and control selection. Give the rationale for the choice of cases and controls<br>*Cross-sectional study* - Give the eligibility criteria, and the sources and methods of selection of participants<br><br>*(b) Cohort study* - For matched studies, give matching criteria and number of exposed and unexposed<br>*Case-control study* - For matched studies, give matching criteria and the number of controls per case		RECORD 6.1: The methods of study population selection (such as codes or algorithms used to identify subjects) should be listed in detail. If this is not possible, an explanation should be provided.<br><br>RECORD 6.2: Any validation studies of the codes or algorithms used to select the population should be referenced. If validation was conducted for this study and not published elsewhere, detailed methods and results should be provided.<br><br>RECORD 6.3: If the study involved linkage of databases, consider use of a flow diagram or other graphical display to demonstrate the data linkage process, including the number of individuals with linked data at each stage.	Methods, subsections "context" and "patient sample" (p6)<br><br>Methods, subsection "exposure variables and software development" (p7&8)<br><br>N/A
Variables	7	Clearly define all outcomes, exposures, predictors, potential confounders, and effect modifiers. Give diagnostic criteria, if applicable.	Methods (p6-9)	RECORD 7.1: A complete list of codes and algorithms used to classify exposures, outcomes, confounders, and effect modifiers should be provided. If these cannot be reported, an explanation should be provided.	Methods (p6-9)
Data sources/ measurement	8	For each variable of interest, give sources of data and details of methods of assessment (measurement).<br>Describe comparability of assessment methods if there is more than one group	Methods (p6-9)		

For peer review only

Bias	9	Describe any efforts to address potential sources of bias	Methods, subsections "covariates" and "statistical analysis" (p8&9)		
Study size	10	Explain how the study size was arrived at	Methods, subsection "patient sample" (p6)		
Quantitative variables	11	Explain how quantitative variables were handled in the analyses. If applicable, describe which groupings were chosen, and why	Methods (p6-9)		
Statistical methods	12	(a) Describe all statistical methods, including those used to control for confounding<br>(b) Describe any methods used to examine subgroups and interactions<br>(c) Explain how missing data were addressed<br>(d) *Cohort study* - If applicable, explain how loss to follow-up was addressed<br>*Case-control study* - If applicable, explain how matching of cases and controls was addressed<br>*Cross-sectional study* - If applicable, describe analytical methods taking account of sampling strategy<br>(e) Describe any sensitivity analyses	Methods (p6-9)		
Data access and cleaning methods		..		RECORD 12.1: Authors should describe the extent to which the investigators had access to the database	Methods (p6-9)

For peer review only

				population used to create the study population.<br><br>RECORD 12.2: Authors should provide information on the data cleaning methods used in the study.	Methods (p6-9)
Linkage		..		RECORD 12.3: State whether the study included person-level, institutional-level, or other data linkage across two or more databases. The methods of linkage and methods of linkage quality evaluation should be provided.	N/A
**Results**					
Participants	13	(a) Report the numbers of individuals at each stage of the study (*e.g.*, numbers potentially eligible, examined for eligibility, confirmed eligible, included in the study, completing follow-up, and analysed)<br>(b) Give reasons for non-participation at each stage.<br>(c) Consider use of a flow diagram	Methods, subsection "patient sample" (p6)	RECORD 13.1: Describe in detail the selection of the persons included in the study (*i.e.,* study population selection) including filtering based on data quality, data availability and linkage. The selection of included persons can be described in the text and/or by means of the study flow diagram.	Methods, subsection "patient sample" (p6)
Descriptive data	14	(a) Give characteristics of study participants (*e.g.*, demographic, clinical, social) and information on exposures and potential confounders<br>(b) Indicate the number of participants with missing data for each variable of interest<br>(c) *Cohort study* - summarise follow-up time (*e.g.*, average and total amount)	Results (p10&11, table 1)		

For peer review only

Outcome data	15	*Cohort study* - Report numbers of outcome events or summary measures over time<br>*Case-control study* - Report numbers in each exposure category, or summary measures of exposure<br>*Cross-sectional study* - Report numbers of outcome events or summary measures	Results (p12&13, table 2)		
Main results	16	(a) Give unadjusted estimates and, if applicable, confounder-adjusted estimates and their precision (e.g., 95% confidence interval). Make clear which confounders were adjusted for and why they were included<br>(b) Report category boundaries when continuous variables were categorized<br>(c) If relevant, consider translating estimates of relative risk into absolute risk for a meaningful time period	Results (p14-19, tables 3-5)		
Other analyses	17	Report other analyses done—e.g., analyses of subgroups and interactions, and sensitivity analyses	Methods and Results (p6-19 - particularly subsection statistical analysis, p9; other tests also mentioned in Results where relevant)		
**Discussion**					
Key results	18	Summarise key results with reference to study objectives	Discussion (p20&21)		
Limitations	19	Discuss limitations of the study, taking into account sources of potential bias or imprecision.	Strengths and Limitations (p23-24)	RECORD 19.1: Discuss the implications of using data that were not created or collected to answer the specific research question(s). Include	Strengths and Limitations (p23-24)

For peer review only

		Discuss both direction and magnitude of any potential bias		discussion of misclassification bias, unmeasured confounding, missing data, and changing eligibility over time, as they pertain to the study being reported.	
Interpretation	20	Give a cautious overall interpretation of results considering objectives, limitations, multiplicity of analyses, results from similar studies, and other relevant evidence	Discussion (p20-23)		
Generalisability	21	Discuss the generalisability (external validity) of the study results	Strengths and Limitations (p23-24)		
**Other Information**					
Funding	22	Give the source of funding and the role of the funders for the present study and, if applicable, for the original study on which the present article is based	Funding and Conflict of Interest (p26)		
Accessibility of protocol, raw data, and programming code		..		RECORD 22.1: Authors should provide information on how to access any supplemental information such as the study protocol, raw data, or programming code.	Methods subsection "exposure variables and software Development" (p7): CRIS open-access online catalogue reference

*Reference: Benchimol EI, Smeeth L, Guttmann A, Harron K, Moher D, Petersen I, Sørensen HT, von Elm E, Langan SM, the RECORD Working Committee. The REporting of studies Conducted using Observational Routinely-collected health Data (RECORD) Statement. *PLoS Medicine* 2015; in press.

*Checklist is protected under Creative Commons Attribution (CC BY) license.

BMJ Open

Investigating the relationship between thought interference, somatic passivity and outcomes in patients with psychosis: a natural-language processing approach using the Clinical Record Interactive Search platform

Journal:	*BMJ Open*
Manuscript ID	bmjopen-2021-057433.R1
Article Type:	Original research
Date Submitted by the Author:	08-Mar-2022
Complete List of Authors:	Magrangeas, Thibault; King's College London Institute of Psychiatry Psychology and Neuroscience<br>Kolliakou, Anna; King's College London Institute of Psychiatry Psychology and Neuroscience, Institute of Psychiatry<br>Sanyal, Jyoti; South London and Maudsley NHS Foundation Trust<br>Patel, Rashmi; King's College London Institute of Psychiatry Psychology and Neuroscience; South London and Maudsley NHS Foundation Trust<br>Stewart, Robert; King's College London Institute of Psychiatry Psychology and Neuroscience; South London and Maudsley NHS Foundation Trust
**Primary Subject Heading**:	Mental health
Secondary Subject Heading:	Health informatics
Keywords:	Schizophrenia & psychotic disorders < PSYCHIATRY, Adult psychiatry < PSYCHIATRY, Health informatics < BIOTECHNOLOGY & BIOINFORMATICS

SCHOLARONE™
Manuscripts

I, the Submitting Author has the right to grant and does grant on behalf of all authors of the Work (as defined in the below author licence), an exclusive licence and/or a non-exclusive licence for contributions from authors who are: i) UK Crown employees; ii) where BMJ has agreed a CC-BY licence shall apply, and/or iii) in accordance with the terms applicable for US Federal Government officers or employees acting as part of their official duties; on a worldwide, perpetual, irrevocable, royalty-free basis to BMJ Publishing Group Ltd ("BMJ") its licensees and where the relevant Journal is co-owned by BMJ to the co-owners of the Journal, to publish the Work in this journal and any other BMJ products and to exploit all rights, as set out in our licence.

The Submitting Author accepts and understands that any supply made under these terms is made by BMJ to the Submitting Author unless you are acting as an employee on behalf of your employer or a postgraduate student of an affiliated institution which is paying any applicable article publishing charge ("APC") for Open Access articles. Where the Submitting Author wishes to make the Work available on an Open Access basis (and intends to pay the relevant APC), the terms of reuse of such Open Access shall be governed by a Creative Commons licence – details of these licences and which Creative Commons licence will apply to this Work are set out in our licence referred to above.

Other than as permitted in any relevant BMJ Author's Self Archiving Policies, I confirm this Work has not been accepted for publication elsewhere, is not being considered for publication elsewhere and does not duplicate material already published. I confirm all authors consent to publication of this Work and authorise the granting of this licence.

For peer review only

Title: Investigating the relationship between thought interference, somatic passivity and outcomes in patients with psychosis: a natural-language processing approach using the Clinical Record Interactive Search platform

Authors: Thibault Magrangeas[1], Anna Kolliakou[1], Jyoti Sanyal[2], Rashmi Patel[1&2], Robert Stewart[1&2]

Affiliations: 1: King's College London (Institute of Psychiatry, Psychology and Neuroscience), London, UK; 2: South London and Maudsley NHS Foundation Trust, London, UK

Corresponding Author: Thibault Magrangeas

Department of Psychological Medicine

Main Building E3.08

Institute of Psychiatry, Psychology and Neuroscience

King's College London

De Crespigny Park

London SE5 8AF

United Kingdom

Telephone: 00447761280858. **Fax:** N/A. **E-mail:** thibault.magrangeas@kcl.ac.uk c/o R Stewart's research group.

Abstract Word Count: 217

Body Word Count: 3963

Abstract

Objectives: We aimed to apply natural language-processing (NLP) algorithms in routine healthcare records to identify reported somatic passivity (external control of sensations, actions and impulses) and thought interference symptoms (thought broadcasting, insertion, withdrawal), first-Rank symptoms traditionally central to diagnosing schizophrenia, and determine associations with prognosis by analysing routine outcomes.

Design: Four algorithms were developed on de-identified mental healthcare data and applied to ascertain recorded symptoms over the three months following first presentation to a mental healthcare provider in a cohort of patients with a primary schizophreniform disorder (ICD-10 F20-F29) diagnosis.

Setting and participants: From the electronic health records of a large secondary mental healthcare provider in south London, 9,323 patients were ascertained from 2007 to the data extraction date (25/02/2020).

Outcomes: The primary binary dependent variable for logistic regression analyses was any negative outcome (Mental Health Act section, >2 antipsychotics prescribed, >22 days spent in crisis care) over the subsequent 2 years.

Results: Final adjusted models indicated significant associations of this composite outcome with baseline somatic passivity (prevalence 4.9%; adjusted odds ratio 1.61, 95% CI 1.37-1.88), thought insertion (10.7%; 1.24, 1.15-1.55) and thought withdrawal (4.9%; 1.36, 1.10-1.69), but not independently with thought broadcast (10.3%; 1.05, 0.91-1.22).

Conclusions: Symptoms traditionally central to the diagnosis of schizophrenia, but under-represented in current diagnostic frameworks, were thus identified as important predictors of short- to medium-term prognosis in schizophreniform disorders.

Keywords: Schizophrenia; Delusions; Prognosis; Psychosis; Patient Outcomes; Natural-Language Processing (NLP)

Strengths and limitations of this study

- This study was carried out on a very large patient sample and identified symptoms from routine care instead of specifically recruited and therefore less representative cohorts, and our applications achieved high-performance metrics.
- The patient sample was drawn from an inner urban area in South London, so results may not be entirely generalisable to the rest of the population.
- The recording of exposure variables relied on symptoms being elicited and noted by clinicians in routine practice and therefore cannot be assumed to be equivalent to those ascertained in a more research-focused interview, just as covariates such as diagnosis also were derived from routine clinical records and should not be assumed to equate research diagnostic criteria; however, we feel that inaccuracies in exposure ascertainment were more likely to obscure findings of interest through non-differential measurement error rather than give rise to spurious associations.
- Although multiple confounding factors were accounted for, our model-fit measures demonstrate model reliability but highlight residual unexplained variance possibly due to the influence of other psychotic symptoms as well as additional external factors which may affect outcomes and should be further investigated. Our model is useful in correlating patient outcomes with symptoms at first presentation but it is not possible to infer stability of the clinical construct over time as the patient is only assessed once.
- Outcomes analysed were restricted to those most readily available from the source data and most likely to be clinically informative; however, they cannot be viewed as exhaustive and there may be elements of prognosis that were not captured in this study, including interactions between outcomes and previous adverse events that may influence symptom occurrence and recording.

For peer review only - http://bmjopen.bmj.com/site/about/guidelines.xhtml

INTRODUCTION

Since Schneider's definition of schizophrenia, diagnostic guidelines have mostly revolved around first-rank symptoms (FRS)[1], including auditory hallucinations, and delusions of perception or thought interference, considered to carry strong diagnostic value. The latter was subdivided into three types of thought interference depending on whether the patient perceived their thoughts to be removed by an external entity, inserted externally or broadcast to the world. Recently, a more holistic approach accounting for a wider variety of symptom profiles has been promoted, culminating in the removal of FRS from DSM-5 diagnostic criteria[2-3]. There are likely to be complex mechanisms underpinning psychosis manifestations and phenomenology. First, different symptoms have been observed to occur in clusters, e.g. somatic passivity and either persecutory delusions[4] or auditory verbal hallucinations[5]. Second, environmental factors seem to influence symptom occurrence, and the cross-cultural relevance of FRS has been questioned[6]. Third, schizophrenia symptoms can directly influence both behaviour and consequently patient outcomes; for example, indirectly leading to violent behaviour through the occurrence of delusions[7]. FRS absence has been found to correlate with better outcomes, leading to a proposed redefinition of schizophrenia as a continuum, exhibiting a positive linear relationship between psychotic symptoms and outcomes[8-10]. Furthermore, the prevalence of individual symptoms appears to vary between early- and late-onset cases, reflecting complex interactions between symptoms and disease development[11-13]. These findings suggest that patient outcomes are better in schizophrenia in the absence of FRS[9] but offer no insight into prognosis once present or into relationships between symptoms.

In agreement with these findings, recent DSM-5 changes have been welcomed as a valuable push towards a better understanding of individual symptoms[14]; however, the same authors

For peer review only - http://bmjopen.bmj.com/site/about/guidelines.xhtml

explain that limitations remain as this new focus does not satisfyingly address the relationship between symptoms, conflicts with most existing guidelines for early symptom detection in schizophrenia that heavily rely on FRS, and diverts attention away from core psychopathological processes at the disorder's root. Recent studies have consistently found thought interference (the specific delusion that thoughts are being inserted in, withdrawn or broadcast from one's head) and somatic passivity (the broader delusion that one's actions and sensations are controlled by an external force) to be core symptoms of psychotic disorders in general and schizophrenia specifically[15-17]. A comparative study of thirteen different diagnostic systems identified a triad of positive symptoms strongly correlated with a schizophrenia diagnosis across all scales, the presence of two of which was sufficient to diagnose 78% of patients; these were auditory hallucinations, disturbances of affect and "passivity feelings" (defined as "any delusion of being influenced or interfered with by imaginary forces from outside, whether somatically or mentally" and therefore assumed to include thought interference)[18]; these symptoms were all already described in Bleuler's 1911 definition of schizophrenia[19]. Somatic passivity and thought interference were also reported to be positively correlated and predict the occurrence of schizophrenia or schizoaffective disorder[20-21]. While some authors have identified differing effects on outcomes, with passivity predicting a worsened prognosis in contrast to interference[9] , others have highlighted a possible link, as both phenomenologically reflect a belief in external control, suggesting they may have a synergistic effect on outcomes[21-23] or even constitute components of the same delusion[5&24] or syndrome[25].

Considering this evidence, clarifying the broader role of FRS in prognosis is crucial to understand which aspects of schizophrenia should be at the core of its diagnosis. With this in mind, this study more specifically aimed to develop four natural language processing (NLP)

algorithms to detect the recording of thought interference subcomponents (broadcasting, insertion, withdrawal) and somatic passivity in text from a large corpus of de-identified mental health records consisting of clinical notes to investigate recorded prevalence, associations with other important positive symptoms of psychosis (paranoia, auditory hallucinations, persecutory delusions), and outcome associations in patients with schizophreniform disorders. Our focus on thought interference and somatic passivity as particular symptoms aimed to clarify the interactions described previously which suggested a particularly strong relationship between these symptoms; in addition, they were chosen as potentially tractable to extraction at scale from routine healthcare data via NLP, being described in relatively consistent language in clinical records and thus a pragmatic choice for further investigation in contrast to other symptoms such as running commentary hallucinations which are typically described with greater linguistic heterogeneity based on the experience rather than the phenomenology and therefore harder to study with this approach.

METHODS

Context

We used data extracted from the electronic mental health clinical notes records of patients at the South London and Maudsley NHS Foundation Trust (SLaM), one of the largest mental health service providers in Europe, serving a geographic catchment of four south London boroughs (Southwark, Lambeth, Lewisham, Croydon) with around 1.3 million residents. The trust provides care to 37,500 active patients, with 12,000 to 14,000 clinical events per week covering a diverse range of interventions in the community and in the inpatient setting[26&27]. SLaM's Clinical Record Interactive Search (CRIS) platform was developed in 2008 to enable research on a de-identified copy of the Trust's electronic health records (used across all services since 2006) within a robust governance and data security framework[24]. CRIS has been described in detail previously[28&29] and supported over 250 research publications to date. Ethical approval as a data resource for secondary analysis in in place (Oxford Research Ethics Committee C, reference 18/SC/0372).

Patient sample

All patients aged 16-95 with a primary F20-F29 disorder diagnosis according to ICD-10 criteria recorded from 2007 to extraction date (25/02/2020) were included. This age-range was chosen to cover patients transitioning to adult mental health care, commonly occurring from age 16; patients with no recorded date of birth appear on the system as aged 100+ and represented all the individuals above age 95; they were therefore excluded to avoid skew when correcting for

For peer review only - http://bmjopen.bmj.com/site/about/guidelines.xhtml

age. According to these criteria, 9,536 patients were identified. Thought interference and somatic passivity occurrence in the three months following first presentation at SLaM (defined as first face-to-face contact) was sought. If multiple diagnoses were then recorded for the same patient, first diagnosis was used. This cut-off was chosen to allow time for clinicians to assess all patients and record their symptoms accurately and focus on patients presenting with these symptoms as first presentation rather than later development. Participants who died within this period were excluded. Of the initial 9,536 patients, 9,323 (97.8%) were included, 57.9% of whom were male. Mean age at inclusion was 39.9 years (SD=17.3).

Exposure variables and software development

CRIS data have been enhanced over the last 10 years by a range of NLP algorithms allowing a hitherto unavailable level of detail to be generated at scale from routine healthcare data. The algorithms, over 90 to date, are described individually in an open-access online catalogue[30], including detailed information on definitions and performance. A particular objective has been to provide, at scale, broader phenotypic information on people presenting with mental disorders than that supplied by clinician-assigned diagnostic codes, through the extraction of recorded symptoms with 60 algorithms to date developed to ascertain individual symptoms across psychotic and affective disorders[31]. Contributing to this wider initiative, and with the specific rationale in mind for this study, we developed four new algorithms to identify instances of recorded somatic passivity and thought insertion, withdrawal and broadcasting in CRIS using the TextHunter platform[32] (figure 1). High performance metrics were attained (supplementary table 1).

For peer review only - http://bmjopen.bmj.com/site/about/guidelines.xhtml

Outcomes

Occurrence of negative outcomes was recorded over the two years following first presentation at SLaM. Three outcomes were evaluated: i) highest-quartile time spent as an inpatient or receiving support from a home treatment team in the community since first presentation (defined as 22 days or more); ii) receiving more than two antipsychotics; iii) involuntary psychiatric hospitalisation defined as detention for assessment or treatment under Sections 2 or 3 of the UK Mental Health Act (MHA).

Covariates

Included covariates were: gender, ethnic group, age at first SLaM presentation, diagnosis, Index of Multiple Deprivation (IMD) score and presence of recorded paranoia, persecutory delusions or auditory hallucinations. Age was categorised by decades for descriptive analyses but applied as a continuous variable otherwise. Diagnoses were recorded as a binary variable, distinguishing schizophrenia (ICD-10 F20x) from other schizophreniform disorders (F21-F29)[33]. The IMD score is a measure of neighbourhood deprivation, applied at Lower Super Output Area level (a standard national address code containing an average 1500 residents), and derived from composite national Census measures. This was divided in quartiles ranging from low (1) to high (4) deprivation. The presence of paranoia, auditory hallucinations, and persecutory delusions, three common psychotic symptoms in schizophrenia either previously suggested to strongly interact with passivity[20,34] or in the case of paranoia to constitute a syndrome distinct from that characterised by the presence of hallucinations or other delusions[24], was also ascertained for adjustment, using previously developed NLP algorithms[31].

For peer review only - http://bmjopen.bmj.com/site/about/guidelines.xhtml

Statistical analysis

All analyses were carried out using Stata 15.1 software. Initial descriptive analyses assessed overlap between symptoms, demographic/clinical factors and other psychotic symptoms. After describing the prevalence of outcomes by covariate, logistic regression models were assembled to investigate associations and their independence in relation to each binary outcome. Adjusted R-squared was used to assess model fit. Secondary analyses studied the impact of adjusting for covariates individually to clarify potential confounding effects. Finally, Poisson regression models investigated the relationship between number of negative outcomes and number of symptoms experienced as well as between number of negative outcomes and each individual symptom. Additional linear regression was carried out to study the relationship between symptoms and number of antipsychotics prescribed, assessing the reliability of the latter as a marker of negative outcome.

Patient and public involvement

The study proposal was reviewed and approved by the patient-led CRIS oversight committee prior to project commencement. No other consultations were made with patients or the public.

For peer review only - http://bmjopen.bmj.com/site/about/guidelines.xhtml

RESULTS

Table 1 describes symptom prevalences by demographic and clinical groups. Thought interference and somatic passivity were more common in younger patients and marginally higher in men compared to women but showed no consistent associations with ethnicity or neighbourhood deprivation. Both symptoms occurred more often in the presence of other psychotic symptoms but did not differ substantially between comparison diagnostic groups. Overlap between subcomponents of thought interference is summarised in Supplementary Figure 1. Supplementary Figure 2 describes overlap between passivity, thought interference and positive symptoms included as covariates.

For peer review only - http://bmjopen.bmj.com/site/about/guidelines.xhtml

Table 1: symptom prevalence by demographic characteristics and diagnoses, and overlap with other psychotic symptoms

		None (%)	Interference without passivity (%)	*Broadcast (%)*	*Insertion (%)*	*Withdrawal (%)*	Passivity without interference (%)	Interference and passivity (%)	Total
	Total participants	77.0	12.8	10.3	10.7	4.9	4.9	5.2	9,323
Age at diagnosis	16-25	65.8	19.3	*16.1*	*17.4*	*7.8*	6.3	8.5	2,131
	26-35	72.4	14.8	*12.7*	*12.5*	*6.0*	5.9	6.9	2,457
	36-45	80.2	11.3	*8.6*	*8.7*	*4.4*	4.3	4.2	1,799
	46-55	82.8	10.2	*6.9*	*8.1*	*3.6*	3.8	3.2	1,241
	56-65	87.6	6.9	*4.1*	*4.8*	*2.0*	3.8	1.7	651
	66-75	91.0	4.8	*3.0*	*4.0*	*0.5*	2.6	1.6	568
	76-85	93.5	3.4	*2.6*	*1.6*	*1.0*	2.9	0.3	383
	86-95	92.5	6.5	*5.4*	*2.2*	*0.0*	1.1	0.0	93
Gender	Female	80.3	11.2	*8.5*	*10.2*	*3.8*	4.4	4.2	3,920
	Male	74.7	14.1	*11.5*	*11.1*	*5.7*	5.3	6.0	5,399
Ethnicity	Black	74.9	14.1	*11.2*	*11.7*	*5.5*	5.4	5.7	3,065
	South Asian	76.1	16.3	*12.0*	*11.6*	*7.3*	2.7	5.0	301
	Chinese	72.4	9.2	*10.5*	*7.9*	*2.6*	10.5	7.9	76
	Other Asian	72.7	13.3	*13.7*	*12.3*	*6.8*	3.8	10.2	293
	White	78.3	12.4	*10.1*	*10.3*	*4.4*	4.6	4.8	3,631
	Other/unknown	79.0	11.4	*8.3*	*9.6*	*4.3*	5.0	4.6	1,957
Index of Multiple Deprivation (IMD)	IMD quartile 1	78.1	12.7	*10.5*	*9.9*	*4.7*	4.4	4.8	2,331
	IMD quartile 2	77.5	12.1	*9.7*	*10.3*	*4.5*	5.1	5.3	2,331
	IMD quartile 3	75.7	13.1	*10.5*	*11.7*	*5.2*	5.3	5.8	2,338
	IMD quartile 4	76.8	13.4	*10.3*	*10.8*	*5.4*	4.8	5.0	2,323
Diagnosis	Schizophrenia diagnosis (F20)	78.2	12.0	*9.8*	*9.5*	*4.7*	4.9	4.9	3,475
	schizotypal/non-mood psychotic disorder (F21-29)	75.3	13.4	*10.5*	*11.4*	*5.0*	4.9	6.5	5,848
Other psychotic symptom	Paranoia present	73.2	15.0	*12.2*	*12.5*	*5.7*	5.7	6.2	7,478
	Paranoia absent	92.7	4.5	*2.5*	*3.5*	*1.8*	1.7	1.1	1,845
	Auditory Hallucinations present	65.8	18.4	*16.2*	*16.7*	*7.3*	6.9	9.0	4,971
	Auditory Hallucinations absent	89.9	6.5	*3.5*	*3.8*	*2.2*	2.6	1.0	4,352
	Persecutory Delusions present	66.2	18.1	*15.6*	*16.4*	*7.0*	7.3	8.4	4,375
	Persecutory Delusions absent	86.0	8.2	*5.6*	*5.6*	*3.1*	2.8	2.4	4984

For peer review only - http://bmjopen.bmj.com/site/about/guidelines.xhtml

Using logistic regression, odds ratios (OR) were calculated to further study overlap between symptoms (Supplementary Table 2), reiterating increased prevalence of thought interference and somatic passivity when other symptoms are present. Thought insertion and broadcasting were strongly correlated (OR=11.67) as were insertion and withdrawal (OR=12.10), whereas there was a weaker association between broadcast and withdrawal (OR=6.05). Somatic passivity was relatively strongly associated with broadcast (OR=5.13) and insertion (OR=6.66) but was more strongly associated with other psychotic symptoms such as paranoia (OR=4.67) and auditory hallucinations (OR=4.99) than with thought withdrawal (OR=3.86).

Table 2 describes outcome distributions. Younger patients, patients from Black ethnic groups and patients from higher deprivation neighbourhoods were at greater risk of negative outcomes. Patients with schizophrenia were less likely to have an MHA section or higher time in crisis care than those with other diagnoses but more likely to be prescribed multiple antipsychotics. Negative outcomes were higher in patients with reported paranoia, auditory hallucinations or persecutory delusions, and in those with thought interference or somatic passivity. Supplementary Figure 3 describes overlap between outcomes.

For peer review only - http://bmjopen.bmj.com/site/about/guidelines.xhtml

Table 2: Two-year incidence of negative outcomes by symptom and covariate groups

	Covariate	Any negative Outcome (%)	MHA section (%)	>2 antipsychotics prescribed (%)	>22 days spent in crisis care (%)	Total participants
Interference/ Passivity	None	42.7	31.8	21.9	10.6	7,181
	Interference without passivity	61.4	46.5	31.6	32.6	1,198
	Broadcast	*63.3*	*47.4*	*34.2*	*34.9*	*956*
	Insertion	*67.4*	*49.9*	*36.6*	*38.1*	*997*
	Withdrawal	*66.9*	*52.1*	*36.2*	*40.1*	*459*
	Passivity without interference	68.2	49.8	36.4	35.7	456
	Interference and passivity	72.3	54.5	42.2	43.2	488
	Total participants	47.9	35.8	24.9	25.0	9,323
Age at diagnosis	16-25	61.0	48.6	32.7	36.8	2,131
	26-35	52.8	40.7	27.0	26.1	2,457
	36-45	55.3	32.9	22.5	21.2	1,799
	46-55	54.1	31.7	23.8	20.9	1,241
	56-65	33.5	21.8	17.8	16.9	651
	66-75	29.9	17.8	15.7	16.2	568
	76-85	24.3	15.4	11.7	13.3	383
	86-95	24.7	14.0	14.0	12.9	93
Gender	Female	46.9	35.2	25.1	24.2	3,920
	Male	48.6	36.2	24.8	25.6	5,399
Ethnicity	Black	57.5	47.6	30.0	31.3	3,065
	South Asian	43.5	30.2	24.9	19.3	301
	Chinese	50.0	44.7	15.8	21.1	76
	Other Asian	48.1	36.2	27.3	23.9	293
	White	43.9	31.1	23.3	22.1	3,631
	Other/unknown	40.7	26.4	20.0	21.7	1,957
Index of Multiple Deprivation (IMD)	IMD quartile 1	45.6	32.9	24.5	23.1	2,331
	IMD quartile 2	46.3	33.6	25.0	24.2	2,331
	IMD quartile 3	50.3	38.1	25.8	26.6	2,338
	IMD quartile 4	49.2	38.4	24.3	26.1	2,323
Diagnosis	Schizophrenia diagnosis (F20)	44.4	30.6	29.9	22.2	3,475
	schizotypal/non-mood psychotic disorder (F21-29)	49.9	38.8	22.0	26.7	5,848
Other symptoms	Paranoia present	53.9	41.0	27.7	28.9	7,478
	Paranoia absent	23.6	14.5	13.5	9.4	1,845
	Auditory Hallucinations present	58.2	43.8	31.3	31.7	4,971
	Auditory Hallucinations absent	36.0	26.6	17.6	17.3	4,352
	Persecutory Delusions present	62.2	49.2	31.9	34.4	4,375
	Persecutory Delusions absent	35.7	23.7	18.6	16.6	4984

For peer review only - http://bmjopen.bmj.com/site/about/guidelines.xhtml

Table 3 summarises logistic regression analyses for any negative outcome. Unadjusted models showed significant associations with somatic passivity and all components of thought interference, and these remained significant after adjustments except thought broadcast. Although confidence intervals overlapped, associations with somatic passivity were consistently strongest. Post-hoc investigations of those with non-overlapping somatic passivity and thought interference showed a significant association with somatic passivity (OR=1.57, 95% CI 1.27-1.95) but not with thought interference (OR=1.11, 95% CI 0.97-1.27).

For peer review only - http://bmjopen.bmj.com/site/about/guidelines.xhtml

Table 3: Unadjusted and adjusted odds ratio for the occurrence of any negative outcome in the presence of thought interference and somatic passivity

Outcome: any negative outcome	**Unadjusted OR (95% CI)**	**Adjusted* OR (95% CI)**	**Adjusted model pseudo R-squared**
Somatic passivity	2.86 (2.47-3.31)	1.61 (1.37-1.88)	0.12
Interference	2.30 (2.07-2.57)	1.25 (1.11-1.41)	0.12
Broadcast	2.01 (1.75-2.31)	1.05 (0.91-1.22)	0.12
Insertion	2.47 (2.15-2.84)	1.24 (1.15-1.55)	0.12
Withdrawal	2.29 (1.88-2.79)	1.36 (1.10-1.69)	0.12
Interference or passivity	2.54 (2.29-2.80)	1.30 (1.19-1.42)	0.12
Both interference and passivity	1.73 (1.57-1.92)	1.24 (1.11-1.38)	0.12
Post-hoc: passivity without interference	2.44 (1.99-2.98)	1.57 (1.27-1.95)	0.12
Post-hoc: interference without passivity	1.88 (1.66-2.13)	1.11 (0.97-1.27)	0.12

*Adjusted for age at diagnosis, gender, ethnicity, diagnosis, IMD quartile and presence of paranoia, auditory hallucinations, persecutory delusion.
Abbreviations: OR = odds ratio; RR = risk ratio

Table 4 displays further analyses of individual outcomes. Associations with somatic passivity remained significant for all after adjustments. Thought interference and insertion specifically were independently associated with higher time spent in crisis care and greater numbers of antipsychotics used, but not with MHA sections. Thought broadcast specifically was not independently associated with any outcome, and thought withdrawal was only associated with higher time spent in crisis care and MHA sections. Aside from thought withdrawal, all other symptoms had the weakest association with MHA sections.

For peer review only - http://bmjopen.bmj.com/site/about/guidelines.xhtml

Table 4: Unadjusted and adjusted odds ratios for associations of each negative outcome with the presence of somatic passivity, thought interference, broadcasting, insertion and withdrawal

Exposure: somatic passivity	Unadjusted OR (95% CI)	Adjusted* OR (95% CI)	Adjusted model pseudo R-squared
Any negative outcome	2.86 (2.47-3.31)	1.61 (1.37-1.88)	0.12
>22 days spent in crisis care	2.15 (1.87-2.48)	1.33 (1.15-1.54)	0.08
MHA section	2.13 (1.86-2.44)	1.23 (1.06-1.42)	0.13
>2 antipsychotics prescribed	2.14 (1.86-2.46)	1.43 (1.23-1.66)	0.07
**Exposure: thought interference**			
Any negative outcome	2.30 (2.07-2.57)	1.25 (1.11-1.41)	0.12
>22 days spent in crisis care	1.89 (1.69-2.12)	1.13 (1.00-1.28)	0.08
MHA section	1.95 (1.75-2.17)	1.08 (0.96-1.21)	0.13
>2 antipsychotics prescribed	1.80 (1.60-2.01)	1.16 (1.02-1.31)	0.07
**Exposure: thought broadcasting**			
Any negative outcome	2.01 (1.75-2.31)	1.05 (0.91-1.22)	0.12
>22 days spent in crisis care	1.71 (1.49-1.97)	1.01 (0.87-1.18)	0.08
MHA section	1.71 (1.50-1.96)	0.92 (0.80-1.07)	0.13
>2 antipsychotics prescribed	1.66 (1.44-1.91)	1.05 (0.90-1.22)	0.07
**Exposure: thought insertion**			
Any negative outcome	2.47 (2.15-2.84)	1.34 (1.15-1.56)	0.12
>22 days spent in crisis care	2.01 (1.75-2.31)	1.21 (1.05-1.40)	0.08
MHA section	1.93 (1.69-2.20)	1.06 (0.92-1.23)	0.13
>2 antipsychotics prescribed	1.88 (1.64-2.16)	1.22 (1.05-1.41)	0.07
**Exposure: thought withdrawal**			
Any negative outcome	2.29 (1.88-2.79)	1.36 (1.10-1.69)	0.12
>22 days spent in crisis care	2.09 (1.73-2.54)	1.37 (1.12-1.68)	0.08
MHA section	2.02 (1.68-2.44)	1.23 (1.01-1.51)	0.13
>2 antipsychotics prescribed	1.76 (1.45-2.14)	1.21 (0.98-1.48)	0.07

*Adjusted for age at diagnosis, gender, ethnicity, diagnosis, IMD quartile and presence of paranoia, auditory hallucinations, persecutory delusion.
Abbreviations: OR = odds ratio; MHA = Mental Health act

For peer review only - http://bmjopen.bmj.com/site/about/guidelines.xhtml

Table 5 summarises analyses investigating adjustments by individual covariates. For both somatic passivity and thought interference, strongest reductions in OR occurred following adjustments for age and the other three psychotic symptoms; however, OR for somatic passivity remained stronger throughout. Estimated marginal means (EM) were calculated to determine the likelihood of negative outcomes at different ages (Supplementary Table 3); this showed a decrease in risk inversely proportional to age: for a patient with somatic passivity, at age 20 EM=59.6(58.0-61.1)%; at age 90, EM=21.5(19.2-23.8)%. For a patient with thought interference, at age 20 EM=59.1(57.6-60.7)%; at age 90, EM=22.2(19.8-24.6)%. Finally, exploratory testing investigating the cumulative relationship between symptoms and outcomes, calculating the odds of experiencing multiple negative outcomes in relation to the sum of individual symptoms and to each individual symptom and illustrated using EM, described a 14.9% increase in the risk of multiple negative outcomes when experiencing no versus all four symptoms, from 73.1% to 88.0%. Consistently with identified trends, somatic passivity was the individual symptom with the greatest impact (adjusted OR=1.16(1.09-1.23)) and thought broadcasting had no significant effect after adjustment (OR=0.99(0.92-1.05)). Regression analysis identified a significant interaction between somatic passivity and number of antipsychotics ($p<0.05$; adjusted R-squared=0.1031). Thought interference and its subcomponents, also included in the model, had no significant effect (supplementary table 4).

For peer review only - http://bmjopen.bmj.com/site/about/guidelines.xhtml

Table 5: Logistic regression analysis of associations with any negative outcome adjusting separately for individual covariates

Outcome: Any negative outcome	**Somatic Passivity**	**Thought Interference**
Unadjusted	2.86 (2.47-3.31)	2.30 (2.07-2.57)
Adjusted for age at diagnosis	2.47 (2.13-2.86)	1.94 (1.73-2.17)
Adjusted for gender	2.85 (2.46-3.30)	2.30 (2.06-2.57)
Adjusted for ethnicity	2.85 (2.46-3.30)	2.28 (2.04-2.55)
Adjusted diagnosis	2.86 (2.47-3.31)	2.30 (2.06-2.56)
Adjusted for IMD quartile	2.86 (2.47-3.31)	2.30 (2.06-2.57)
Adjusted paranoia	2.43 (2.09-2.82)	1.94 (1.74-2.17)
Adjusted for auditory hallucinations	2.23 (1.92-2.59)	1.80 (1.61-2.02)
Adjusted for persecutory delusions	2.23 (1.91-2.59)	1.82 (1.63-2.04)

DISCUSSION

Having developed NLP algorithms to extract recorded thought interference and somatic passivity in routine electronic mental health records, we investigated their associations with negative outcomes over a 2-year period following first-recorded diagnosis of schizophrenia or a schizophreniform disorder. Both symptoms were associated with a significantly increased risk of experiencing negative outcomes, somatic passivity having the strongest associations after full adjustment. This is broadly consistent with previous reports that experiencing these symptoms is predictive of negative outcomes: Stephen et al found the absence of somatic passivity to correlate with better outcomes in 66% of patients[8]; Koehler found no significant difference in outcomes between patients experiencing any thought interference subcomponent or somatic passivity but concluded that their absence was predictive of a better prognosis[9]. In 2019, Malinowski et al found that thought broadcasting seemed predictive of disease continuation without remission, but that this effect was no longer significant after adjusting for external factor influence (specifically, untreated psychosis duration and drug abuse)[35]; accordingly, although different covariates were included, our results suggest that thought broadcast does not have a significant effect on outcome. Similar prevalence and overlap between thought insertion, withdrawal and broadcasting suggests that this cannot be due to broadcasting occurring more frequently in isolation. It is interesting to note that aside from broadcasting, these symptoms remained significantly associated with negative outcomes after including common non first-rank symptoms of schizophrenia in our covariates, namely paranoia, auditory hallucinations and persecutory delusions, suggesting the importance of their link with negative outcomes. Secondary testing identified a unique significant effect of somatic passivity on number of antipsychotics prescribed, possibly reflecting poorer response leading to worsened outcomes; importantly, this outcome may reflect a degree of treatment resistance

For peer review only - http://bmjopen.bmj.com/site/about/guidelines.xhtml

not investigated here but likely to influence patient prognosis. Our findings suggest that somatic passivity and thought interference subcomponents correlate with an increased risk of experiencing negative outcomes, some more strongly than others. In contrast, it is interesting to note that no particular outcome appeared to be disproportionately correlated with symptoms. Although the odds ratio of an MHA section being in place were less strong than other outcomes, this reflects that their use is reserved for the most unwell patients who refuse or lack capacity to accept voluntary community treatment. Overall, the odds of all three outcomes increased in a similar fashion in the presence of thought interference and somatic passivity, reflecting a generally worsened prognosis.

Nuevo et al described schizophrenia symptoms as a continuum whereby the experience of multiple symptoms predicts a worse prognosis[10]. Our finding that patients with either or both thought interference and somatic passivity were more likely to experience negative outcomes than those for whom these symptoms were absent supports this. Exploratory analyses suggested a significant relationship between number of symptoms experienced and negative outcome occurrence; however, our results also suggest that each symptom has a different impact on outcomes which was not always individually significant, and that the presence of multiple symptoms may even sometimes reduce the frequency of negative outcomes: for example, patients experiencing somatic passivity only may have poorer outcomes than those with both this and thought interference. One explanation may be that reporting thought interference to clinicians requires greater insight from the patient, contributing to better outcomes and facilitating voluntary treatment such as that offered by crisis care teams with which more patients experiencing thought insertion and withdrawal engaged. The recording of exposure variables relied on symptoms being elicited and noted by clinicians in routine practice and therefore cannot be assumed to be equivalent to those ascertained in a more research-

For peer review only - http://bmjopen.bmj.com/site/about/guidelines.xhtml

focused interview, just as covariates such as diagnosis also were derived from routine clinical records and should not be assumed to equate research diagnostic criteria; however, we feel that inaccuracies in exposure ascertainment were more likely to obscure findings of interest through non-differential measurement error rather than give rise to spurious associations. Unfortunately, although mandatory hospitalisation under the MHA reflects a need for crisis care, it is not possible with these algorithms to determine what interventions patients received and responded to in that context specifically. However, guideline consistency since 2007 suggests that patients represented in this analysis should have received similar standards of care across the time period sampled. Overall, although there does seem to be a trend of worsening outcomes as symptoms accumulate, it would be overly simplistic to claim that this effect is purely additive.

Interestingly, of the covariates investigated, only age had a substantial confounding effect, negatively correlated with symptoms and outcomes of interest. In the past, thought insertion and withdrawal have been reported as more common in early-onset cases and somatic passivity as more frequent in late-onset cases[11]. Because of challenges in establishing whether patients were previously diagnosed in another service before presenting at SLaM, it is not possible to equate first presentation here with first-episode psychosis, particularly in older individuals who may have longer standing diagnoses and recently in-migrated to the catchment. This first presentation sample is therefore better viewed as one which is enriched with first-episode cases rather than directly equivalent. Furthermore, as our database consists of real-life data patients do not always have consistent follow-up or repeat assessments, including due to external factors not related to the patient's care. Our model is therefore useful in correlating patient outcomes with symptoms at first presentation but it is not possible to infer stability of the clinical construct over time as the patient is only assessed once. Although age at diagnosis may

For peer review only - http://bmjopen.bmj.com/site/about/guidelines.xhtml

not accurately reflect age at disease onset and doesn't account for possible changes in symptom profile as patients age, our results suggest that both symptoms are more frequent in younger patients, leading to a worsened prognosis. In this context, it would be interesting, in future research, to investigate symptom stability over time and how prognosis might also change with this.

Other external factors such as level of education or spiritual beliefs may play a role in the personal interpretation of abnormal experiences and have consequently affected outcomes. We developed a model that suggested the existence of significant interactions between thought interference, somatic passivity and the occurrence of negative outcomes. As shown in supplementary figures 1 and 2, these symptoms overlapped in many patients as well as with other psychotic symptoms, and so did the negative outcomes under scrutiny. The algorithms we developed to create this model achieved high performance metrics and our model-fit measures demonstrate model reliability but highlight that the variance remains in great part unexplained, likely due to the role of other factors not included here. This could be usefully explored in further studies to refine models assessing patient prognosis. Furthermore, our patient sample was drawn from an inner-urban South-London population and results may be different in other patient groups, although the large sample size should mitigate this effect.

Paranoia, persecutory delusions and auditory hallucinations were included as covariates due to past evidence suggesting a correlation with thought interference and somatic passivity. The primary purpose of this study was to characterise symptoms of thought interference and passivity, and other symptoms, such as negative and disorganised symptoms, were not included. Broader symptom profiles should be investigated in future research. Interactions between outcomes should also be studied; for example, it is not unreasonable to propose that

For peer review only - http://bmjopen.bmj.com/site/about/guidelines.xhtml

the prescription of fewer antipsychotics may correlate with reduced hospitalisation time. To this effect, a harmonisation of definitions would be useful to enable the development of algorithms studying symptoms described more heterogeneously. Outcomes analysed were restricted to those most readily available from the source data and most likely to be clinically informative; however, they cannot be viewed as exhaustive and there may be elements of prognosis that were not captured in this study, including interactions between outcomes and previous adverse events that may influence symptom occurrence and recording.

Although to be treated with caution, as not tested for significance, our descriptive results provide potential directions for future research and support the notion that psychotic symptoms share more complex interactions than previously described. Somatic passivity has been suggested to cluster with different symptoms such as persecutory delusions[4] or auditory-verbal hallucinations[5], and thought interference has sometimes been described as a component of somatic passivity[18&36]. We found that subcomponents of thought interference traditionally assumed to closely correlate may actually more commonly occur alongside other symptoms; e.g., the odds of experiencing thought insertion alongside somatic passivity were greater than those of withdrawal and broadcasting co-occurring. In a study attempting to link delusions to putative underlying neurobiological mechanisms, Kimhy et al called for new symptom definitions that would focus on underlying mechanisms rather than phenomenological descriptions[23]. Each delusion was described then grouped with others based on correlation, enabling the grouping of symptoms that share significant interactions while maintaining their individuality. This approach enables a less biased study of psychotic symptoms relying less on traditional groupings and may allow more accurate descriptions of symptom interactions. Additionally, thought interference and somatic passivity have previously been found to be closely related and predictive of schizophrenia[13&36-38]. Our results support reports that these

For peer review only - http://bmjopen.bmj.com/site/about/guidelines.xhtml

symptoms share a positive correlation[20] but question their specificity to schizophrenia and usefulness in differentiating this from other psychotic disorders, particularly as both were absent in 77% of patients. Somatic passivity has been reported to be the most common symptom experienced in schizophrenia[6] and first-episode psychosis[24]; however, in our study thought interference was almost twice as commonly recorded. Overall, it would be interesting to repeat these analyses in the future with a broader range of diagnoses including affective psychoses in which they also occur to determine whether these symptoms could be more specific to other conditions or used to assess patient prognosis.

For peer review only - http://bmjopen.bmj.com/site/about/guidelines.xhtml

CONCLUSION

This study developed NLP applications to detect the recorded presence of somatic passivity, thought interference and its subcomponents in patients within three months of their first presentation and analysed negative outcome occurrence over the two following years to investigate the relationship between these specific FRS and patient prognosis. We observed a significant effect of somatic passivity, thought interference and its subcomponents thought insertion and withdrawal on the likelihood of experiencing any negative outcome. We also offer evidence that psychotic symptoms traditionally associated with schizophrenia may not be specific to this disorder and share complex interactions, putting in question current definitions and warranting further investigation to determine whether it is correct to consider thought insertion, withdrawal and broadcasting as parts of the same symptom. Overall, the presence of somatic passivity and thought interference was found to significantly increase the risk of experiencing negative outcomes in psychosis; somatic passivity had the greatest effect. Future research should focus on acquiring a better understanding of these effects, including by investigating other psychotic symptoms to determine the more detailed pathways through which these interact with prognosis in schizophrenia and other types of psychosis. Future studies should investigate these correlations and reliable markers of outcomes to develop standardised tools enabling a better evaluation of patient prognosis. In addition, it would be interesting to study which antipsychotics specifically were prescribed and whether patients may have had a better response to specific medication, reflective of a general lack of response to individual antipsychotics in schizophrenia rather than the presence of refractory disease.

For peer review only - http://bmjopen.bmj.com/site/about/guidelines.xhtml

Funding

RS, RP and AK are part-funded by the National Institute for Health Research (NIHR) Biomedical Research Centre at the South London and Maudsley NHS Foundation Trust and King's College London. RS is additionally part-funded by: i) an NIHR Senior Investigator Award; ii) the National Institute for Health Research (NIHR) Applied Research Collaboration South London (NIHR ARC South London) at King's College Hospital NHS Foundation Trust; iv) the DATAMIND HDR UK Mental Health Data Hub (MRC grant MR/W014386). The views expressed are those of the authors and not necessarily those of the NIHR or the Department of Health and Social Care. RP has received funding from an NIHR Advanced Fellowship (NIHR301690), a Medical Research Council (MRC) Health Data Research UK Fellowship (MR/S003118/1) and a Starter Grant for Clinical Lecturers (SGL015/1020) supported by the Academy of Medical Sciences, The Wellcome Trust, MRC, British Heart Foundation, Arthritis Research UK, the Royal College of Physicians and Diabetes UK.

Conflict of Interest

RS declares research support received in the last 36 months from Janssen, GSK and Takeda. RP has received grant funds from Janssen and consultancy fees from Induction Healthcare and Holmusk.

Author Statement

TM, AK, JS, RP and RS planned the study together. JS developed the applications, which were doubly annotated by TM and AK. JS and AK retrieved the data, and TM analysed it with advice from AK, JS, RP and RS. TM wrote the largest part of the paper, produced the tables and figures and submitted it, working under supervision from AK and RS. AK, RP and RS commented on and directly contributed to the manuscript.

Ethics Statement

The source de-identified data are made available for secondary analyses as approved by Oxford Research Ethics Committee C, reference 18/SC/0372.

Data availability statement

No additional data are available.

Figure Legends

Figure 1: Flowchart illustrating the development of algorithms via the TextHunter platform. TextHunter allows a training dataset of text strings containing wording of interest to be presented in an easy visual form for researcher annotation. The development process is identical for all algorithms relating to symptoms recorded in clinical text. In the final step, testing the performance of the algorithm over the entire CRIS database was performed by checking 100 random annotated documents produced by the algorithm and 100 random unannotated documents using keyword searches. A second researcher double-annotated a proportion of the retrieved documents to determine inter-rater reliability (IRR) using Cohen's kappa. The process was iterative and sought to achieve P and R values over 80%.

For peer review only - http://bmjopen.bmj.com/site/about/guidelines.xhtml

REFERENCES

1. Schneider, K. *Klinische Psychopathologie* (5th edn) (Trans. Hamilton). New York: Grune & Stratton; 1959.
2. American Psychiatric Association. *Diagnostic and Statistical Manual of Mental Disorders, Fifth Edition (DSM-5)*. Washington, DC: American Psychiatric Association; 2013.
3. Kendler KS. Phenomenology of Schizophrenia and the Representativeness of Modern Diagnostic Criteria. *Jama Psychiat*. 2016;73(10):1082 doi:10.1001/jamapsychiatry.2016.1976 [published Online First: October 2016]
4. Fierro M, Hernández AM, Malcolm J. The Psychopathology of Delusion of Control According to Subjective Experience. *Revista Colombiana De Psiquiatría*. 2018;47(4):221-228 doi:10.1016/j.rcpeng.2018.10.005 [published Online First: December 2018]
5. Rosen C, Jones N, Chase KA, et al. The intrasubjectivity of self, voices and delusions: A phenomenological analysis. *Psychos*. 2016;8(4):357-368 doi: 10.1080/17522439.2016.1162839 [published Online First: April 2016]
6. Malik SB, Ahmed M, Bashir A, et al. Schneider's First-Rank Symptoms of Schizophrenia: Prevalence and Diagnostic Use. *Brit J Psychiat*. 1990;156(1):109-111 doi: 10.1192/bjp.156.1.109 [published in print First: January 1990]
7. Ullrich S, Keers R, Coid JW. Delusions, anger, and serious violence: new findings from the MacArthur Violence Risk Assessment Study. *Schizophrenia Bull*. 2013;40(5):1174-1181 doi: doi.org/10.1093/schbul/sbt126 [published Online First: September 2013]
8. Stephens JH, Richard P, McHugh PR. Long-Term Follow-up of Patients Hospitalized for Schizophrenia, 1913 to 1940. *J Nerv Amp Ment Dis*. 1997;185(12):715-721.
9. Koehler K. Prognostic Prediction in RDC Schizo-Affective Disorder on the Basis of First-Rank Symptoms Weighted in Terms of Outcome. *Psychopathology*. 1983;16(2-4):186-197 doi: 10.1159/000283967 [published in print First: 1983]

For peer review only - http://bmjopen.bmj.com/site/about/guidelines.xhtml

10. Nuevo R, Chatterji S, Verdes E, et al. The continuum of psychotic symptoms in the general population: a cross-national study. *Schizophrenia Bull*. 2010;38(3):475-485 doi: 10.1093/schbul/sbq099 [published Online First: September 2010]

11. Howard R, Castle D, Wessely S, et al. A Comparative Study of 470 Cases of Early-Onset and Late-Onset Schizophrenia. *Brit J Psychiat*. 1993;163(3):352-357 doi: 10.1192/bjp.163.3.352 [published in print First: September 1983]

12. Alici-Evcimen Y, Ertan T, Eker E. Case Series With Late-Onset Psychosis Hospitalized in a Geriatric Psychiatry Unit in Turkey: Experience in 9 Years. *Int Psychogeriatr*. 2003;15(1):69-72 doi: 10.1017/S1041610203008767 [published Online First: March 2003]

13. Naqvi HA, Hussain S, Islam M, et al. Early psychosis symptoms. *J Coll Physicians Surg Jcpsp*. 2014;24(3):198-202.

14. Nemeroff CB, Weinberger D, Rutter M, et al. DSM-5: a collection of psychiatrist views on the changes, controversies, and future directions. *Bmc Med*. 2013;11(1):202 doi: 10.1186/1741-7015-11-202 [published Online First: September 2013]

15. Chapman LJ, Chapman JP. Scales for Rating Psychotic and Psychotic-like Experiences as Continua. *Schizophrenia Bull*. 1980;6(3):476-489 doi: 10.1093/schbul/6.3.476 [published in print First: January 1980]

16. Chapman LJ, Edell WS, Chapman JP. Physical Anhedonia, Perceptual Aberration, and Psychosis Proneness. *Schizophrenia Bull*. 1980;6(4):639-653 doi: 10.1093/schbul/6.3.476 [published in print First: January 1980]

17. Kwapil TR, Chapman LJ, Chapman J. Validity and Usefulness of the Wisconsin Manual for Assessing Psychotic-like Experiences. *Schizophrenia Bull*. 1999;25(2):363-375 doi: 10.1093/oxfordjournals.schbul.a033384 [published in print First: 1999]

For peer review only - http://bmjopen.bmj.com/site/about/guidelines.xhtml

18. Landmark J, Merskey H, Cernovsky Z, et al. The positive triad of schizophrenic symptoms. Its statistical properties and its relationship to 13 traditional diagnostic systems. *Br J Psychiatry J Ment Sci*. 1990;156(3):388-394. doi: 10.1192/bjp.156.3.388 [published Online First: July 2011]

19. Bleuler, E. *Dementia Praecox oder Gruppe der Schizophrenien*. Leipzig: Germany Deuticke; 1911.

20. Cloninger CR, Martin RL, Guze SB, et al. Diagnosis and Prognosis in Schizophrenia. *Arch Gen Psychiat*. 1985;42(1):15 doi: 10.1001/archpsyc.1985.01790240017002 [published in print First: January 1985]

21. Loftus J, Delisi LE, Crow TJ. Factor structure and familiality of first-rank symptoms in sibling pairs with schizophrenia and schizoaffective disorder. *Brit J Psychiat*. 2000;177(1):15-19 doi: 10.1192/bjp.177.1.15 [published Online First: July 2000]

22. Stanghellini G, Monti MR. Influencing and being influenced: the other side of "bizarre delusions". 2. Clinical investigation. *Psychopathology*. 1993;26(3-4):165-169 doi: 10.1159/000284817 [published in print First: February 1993]

23. Kimhy D, Goetz R, Yale S, et al. Delusions in Individuals with Schizophrenia: Factor Structure, Clinical Correlates, and Putative Neurobiology. *Psychopathology*. 2005;38(6):338-344 doi: 10.1159/000089455 [published Online First: December 2005]

24. Fernandes AC, Cloete D, Broadbent MTM, et al. Development and evaluation of a de-identification procedure for a case register sourced from mental health electronic records. *Bmc Med Inform Decis*. 2013;13(1):71 doi: 10.1186/1472-6947-13-71 [published Online First: July 2013]

25. Stuart GW, Malone V, Currie J, et al. Positive and negative symptoms in neuroleptic-free psychotic inpatients. *Schizophr Res*. 1995;16(3):175-188 doi: 10.1016/0920-9964(94)00083-K [published in print First: August 1995]

For peer review only - http://bmjopen.bmj.com/site/about/guidelines.xhtml

26. Patel R, Irving J, Brinn A, *et al.* Impact of the COVID-19 pandemic on remote mental healthcare and prescribing in psychiatry: an electronic health record study. *Bmj Open* 2021;**11**:e046365. doi:10.1136/bmjopen-2020-046365.

27. South London and Maudsley NHS Foundation Trust. Our Services, Types of service. https://slam.nhs.uk/our-services/types-of-service/ (accessed 21 Feb. 2022).

28. Stewart R, Soremekun M, Perera G, et al. The South London and Maudsley NHS Foundation Trust Biomedical Research Centre (SLAM BRC) case register: development and descriptive data. *Bmc Psychiatry*. 2009;9(1):51 doi: 10.1186/1471-244X-9-51 [published Online First: August 2009]

29. Perera G, Broadbent M, Callard F, et al. Cohort profile of the South London and Maudsley NHS Foundation Trust Biomedical Research Centre (SLaM BRC) Case Register: current status and recent enhancement of an Electronic Mental Health Record-derived data resource. *Bmj Open*. 2016;6(3):e008721 doi: 10.1136/bmjopen-2015-008721 [published Online First: March 2016]

30. CRIS NLP Service. CRIS Natural Language Processing Applications Library (2020), v1.2. Maudsley Biomedical Research Centre. https://www.maudsleybrc.nihr.ac.uk/facilities/clinical-record-interactive-search-cris/cris-natural-language-processing/. Updated 12/06/20. Accessed 03/08/20.

31. Jackson RG, Patel R, Jayatilleke N, et al. Natural language processing to extract symptoms of severe mental illness from clinical text: the Clinical Record Interactive Search Comprehensive Data Extraction (CRIS-CODE) project. *Bmj Open*. 2017;7(1):e012012 doi: 10.1136/bmjopen-2016-012012 [published Online First: January 2017]

32. Jackson RG, Ball M, Patel R, et al. TextHunter--A User Friendly Tool for Extracting Generic Concepts from Free Text in Clinical Research. *Amia Annu Symposium Proc Amia*

For peer review only - http://bmjopen.bmj.com/site/about/guidelines.xhtml

Symposium Amia Symposium. 2014;2014:729-738 doi: 10.13140/2.1.3722.9121 [published Online First: November 2014]

33. World Health Organisation. *International Statistical Classification of Diseases and Related Health Problems, 10th Revision (ICD-10)*. Geneva: WHO; 1992.
34. Galdos P, Os J van. Gender, psychopathology, and development: from puberty to early adulthood. *Schizophr Res*. 1995;14(2):105-112 doi: 10.1016/0920-9964(94)00020-9 [published in print First: January 1995]
35. Malinowski FR, Tasso B de C, Ortiz BB, et al. Schneider's first-rank symptoms as predictors of remission in antipsychotic-naive first-episode psychosis. *Revista Brasileira De Psiquiatria Sao Paulo Braz 1999*. 2019;42(AHEAD):22-26 doi: 10.1590/1516-4446-2018-0237 [published Online First: February 2020]
36. Klosterkötter J, Hellmich M, Steinmeyer EM, et al. Diagnosing Schizophrenia in the Initial Prodromal Phase. *Arch Gen Psychiat*. 2001;58(2):158 doi: 10.1001/archpsyc.58.2.158 [published Online First: February 2001]
37. Moller P, Husby R. The Initial Prodrome in Schizophrenia: Searching for Naturalistic Core Dimensions of Experience and Behavior. *Schizophrenia Bull*. 2000;26(1):217-232 doi: 10.1093/oxfordjournals.schbul.a033442 [published Online First: January 2000]
38. Galdos P, Os J van. Gender, psychopathology, and development: from puberty to early adulthood. *Schizophr Res*. 1995;14(2):105-112 doi: 10.1016/0920-9964(94)00020-9 [published in print First: January 1995]

For peer review only - http://bmjopen.bmj.com/site/about/guidelines.xhtml

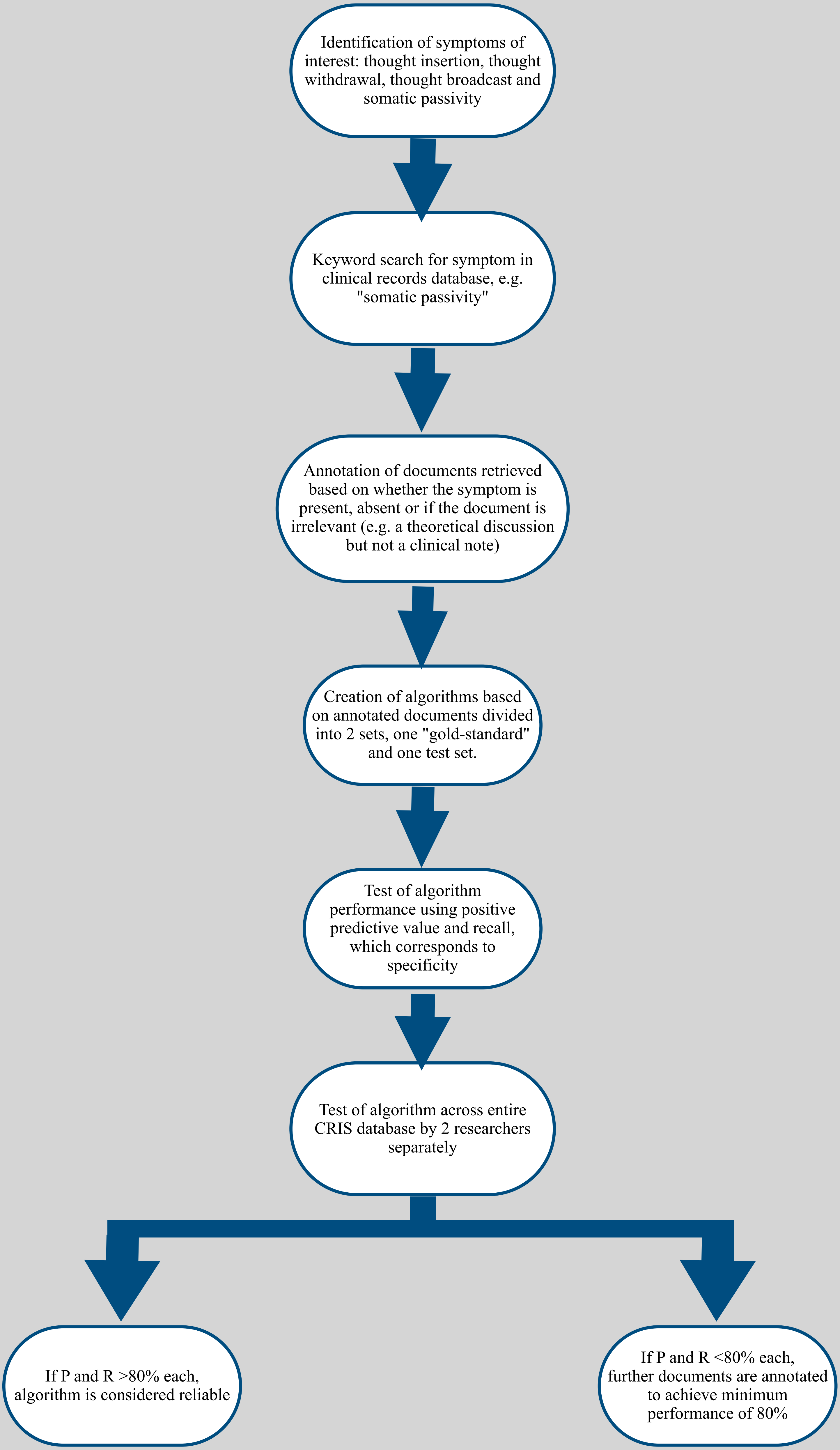
Identification of symptoms of interest: thought insertion, thought withdrawal, thought broadcast and somatic passivity
Keyword search for symptom in clinical records database, e.g. "somatic passivity"
Annotation of documents retrieved based on whether the symptom is present, absent or if the document is irrelevant (e.g. a theoretical discussion but not a clinical note)
Creation of algorithms based on annotated documents divided into 2 sets, one "gold-standard" and one test set.
Test of algorithm performance using positive predictive value and recall, which corresponds to specificity
Test of algorithm across entire CRIS database by 2 researchers separately
If P and R >80% each, algorithm is considered reliable
If P and R <80% each, further documents are annotated to achieve minimum performance of 80%

Supplementary Material

Supplementary Table 1: Natural language processing algorithm development and performance. A minimum P and R of 80% is required for an algorithm to be considered reliable. In the context of passivity, the lower IRR led to the inclusion of extra annotations that enabled us to reach this threshold.

Symptom	**Keyword**	**Number of annotations**	**IRR**	**P and R**	**Extra steps required**	**Manual testing**	**Keyword search P and R**
Thought insertion	"thought insert*"	592 (96 double-annoted)	97 %	P=81%; R=94%	N/A	P=84%	P=81%; R=96%
Thought broadcasting	"thought broadcast*"	398 (95 double-annoted)	94 %	P=81%; R=94%	N/A	P=84%	P=81%; R=96%
Thought withdrawal	"thought withdraw*"	500 (76 double-annotated)	95 %	P=74%; R=91%	159 extra annotations	P=84%	P= 90%; R=88%
Somatic passivity	"passivity"	500 (116 double-annotated)	71 %	P=72%; R=92%	597 extra annotations, form excluded	P=82%	P=89%; R=100%

Supplementary Table 2: Unadjusted odds ratios for the co-occurrence of psychotic symptoms across the patient sample

Symptom	Broadcast	Insertion	Withdrawal	Passivity	Paranoia	Auditory Hallucinations	Persecutory Delusions
Broadcast	1						
Insertion	11.67	1					
Withdrawal	6.05	12.10	1				
Passivity	5.13	6.66	3.86	1			
Paranoia	5.29	3.97	3.32	4.67	1		
Auditory Hallucinations	5.33	5.06	3.45	4.99	3.50	1	
Persecutory Delusions	3.13	3.30	2.41	3.40	5.30	2.80	1

Supplementary Table 3: Prevalence of negative outcomes in patients with somatic passivity or thought interference at different ages

Age (years)	Occurrence on negative outcomes in patients with somatic passivity (%) (95% CI)	Occurrence on negative outcomes in patients with thought interference (%) (95% CI)
20	59.6 (58.0-61.1)	59.1 (57.6-60.7)
30	53.7 (52.5-54.9)	53.4 (52.2-54.6)
40	47.7 (46.6-48.7)	47.7 (46.6-48.7)
50	41.7 (40.5-43.0)	41.9 (40.7-43.2)
60	36.1 (34.5-37.6)	36.4 (34.8-38.0)
70	30.7 (28.8-32.6)	31.2 (29.3-33.2)
80	25.9 (23.7-28.0)	26.5 (24.3-28.7)
90	21.5 (19.2-23.8)	22.2 (19.8-24.6)

For peer review only - http://bmjopen.bmj.com/site/about/guidelines.xhtml

Supplementary Table 4: Linear regression describing the influence of somatic passivity, thought interference and its subcomponents on the number of antipsychotics prescribed

Symptom	Standardized Beta Coefficients (95% CI)	t	Significance
Somatic Passivity	0.35 (0.24-0.45)	6.43	0.00
Thought Interference	0.40 (-0.14-0.22)	0.43	0.67
Thought Insertion	0.14 (0-0.01-0.30)	1.81	0.07
Thought Withdrawal	-0.00 (-0.17-0.16)	-0.05	0.96
Thought Broadcasting	-0.02 (-0.18-0.13)	-0.29	0.77

Adjusted for age at diagnosis, gender, ethnicity, diagnosis, IMD quartile and presence of paranoia, auditory hallucinations, persecutory delusion.

For peer review only - http://bmjopen.bmj.com/site/about/guidelines.xhtml

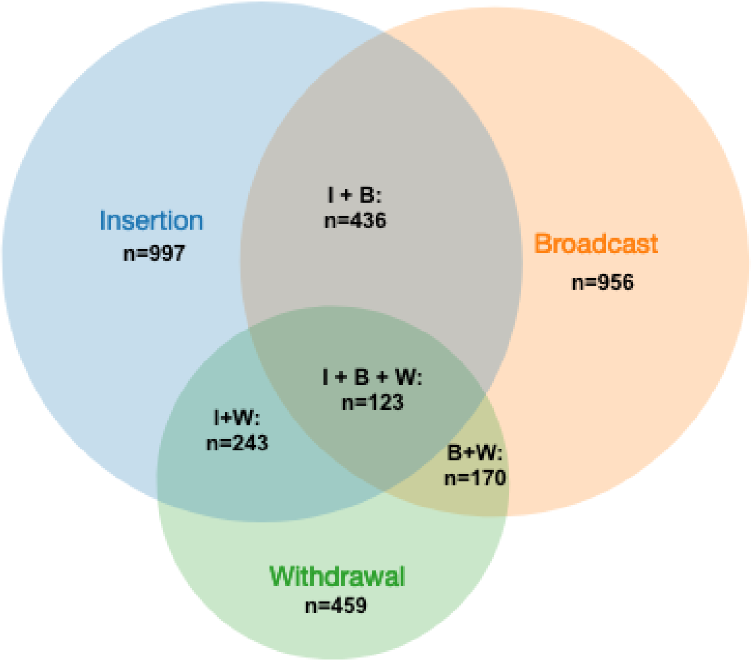

Supplementary Figure 1: Venn diagram describing the overlap between thought broadcasting, thought insertion and thought withdrawal

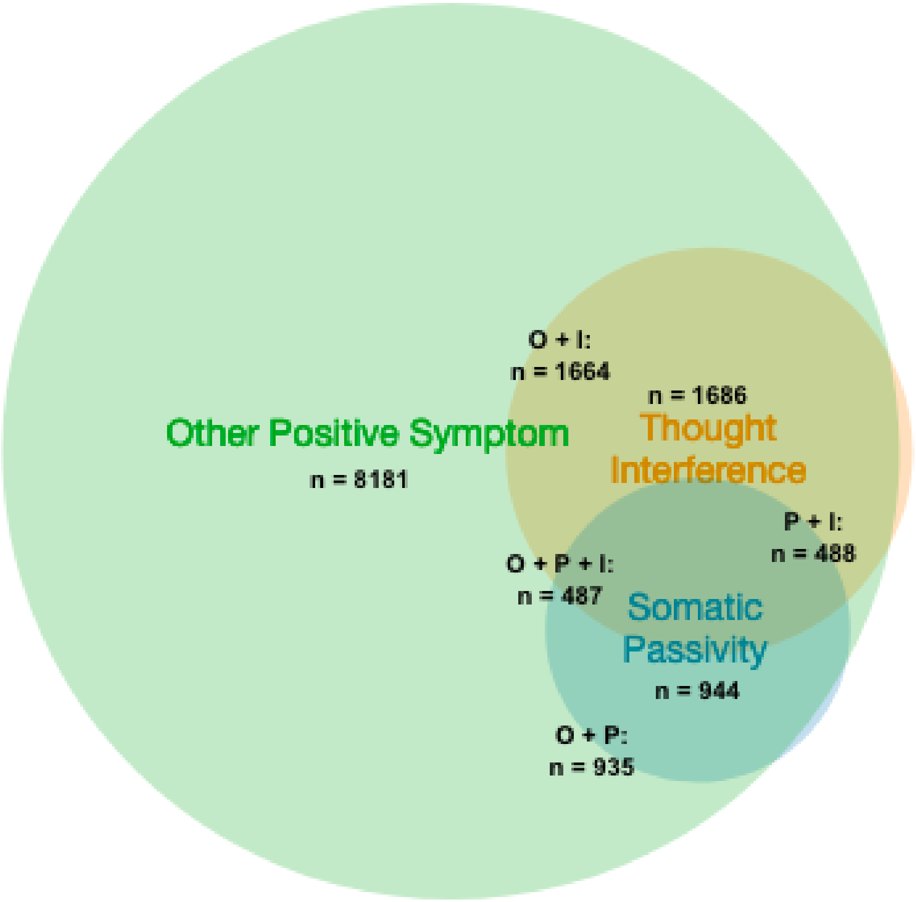

Supplementary figure 2: Venn diagram describing the overlap between somatic passivity, thought interference and any other positive symptoms (auditory hallucinations, persecutory delusions, paranoia) . I: Thought Interference; P: Passivity; O: Other Positive Symptom. When carrying out analyses, other positive symptoms were not combined but each included as individual covariates.

For peer review only - http://bmjopen.bmj.com/site/about/guidelines.xhtml

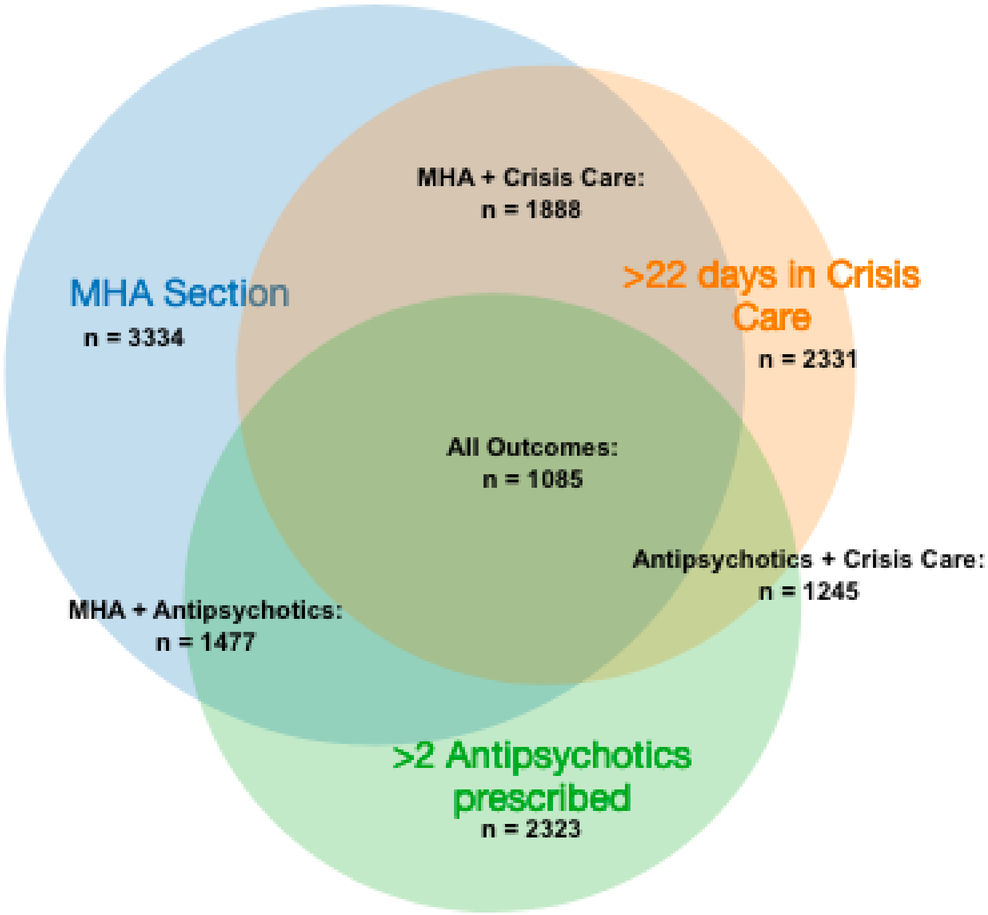

Supplementary Figure 3: Venn diagram describing the overlap between Outcomes investigated. MHA: presence of a Mental Health act section.

For peer review only - http://bmjopen.bmj.com/site/about/guidelines.xhtml

The RECORD statement – checklist of items, extended from the STROBE statement, that should be reported in observational studies using routinely collected health data.

	Item No.	STROBE items	Location in manuscript where items are reported	RECORD items	Location in manuscript where items are reported
**Title and abstract**					
	1	(a) Indicate the study's design with a commonly used term in the title or the abstract (b) Provide in the abstract an informative and balanced summary of what was done and what was found	Title page and abstract (p1&2)	RECORD 1.1: The type of data used should be specified in the title or abstract. When possible, the name of the databases used should be included.<br><br>RECORD 1.2: If applicable, the geographic region and timeframe within which the study took place should be reported in the title or abstract.<br><br>RECORD 1.3: If linkage between databases was conducted for the study, this should be clearly stated in the title or abstract.	Title page (p1) and abstract (p2)<br><br>Abstract (p2)<br><br>N/A
**Introduction**					
Background rationale	2	Explain the scientific background and rationale for the investigation being reported	Introduction (p3-5)		
Objectives	3	State specific objectives, including any prespecified hypotheses	Abstract (p2) and Introduction (p4&5, final paragraph)		
**Methods**					
Study Design	4	Present key elements of study design early in the paper	Methods (p6-9)		
Setting	5	Describe the setting, locations, and relevant dates, including periods of recruitment, exposure, follow-up, and data collection	Methods, (p6-9)		

For peer review only

Participants	6	*(a) Cohort study* - Give the eligibility criteria, and the sources and methods of selection of participants. Describe methods of follow-up<br>*Case-control study* - Give the eligibility criteria, and the sources and methods of case ascertainment and control selection. Give the rationale for the choice of cases and controls<br>*Cross-sectional study* - Give the eligibility criteria, and the sources and methods of selection of participants<br><br>*(b) Cohort study* - For matched studies, give matching criteria and number of exposed and unexposed<br>*Case-control study* - For matched studies, give matching criteria and the number of controls per case		RECORD 6.1: The methods of study population selection (such as codes or algorithms used to identify subjects) should be listed in detail. If this is not possible, an explanation should be provided.<br><br>RECORD 6.2: Any validation studies of the codes or algorithms used to select the population should be referenced. If validation was conducted for this study and not published elsewhere, detailed methods and results should be provided.<br><br>RECORD 6.3: If the study involved linkage of databases, consider use of a flow diagram or other graphical display to demonstrate the data linkage process, including the number of individuals with linked data at each stage.	Methods, subsections "context" and "patient sample" (p6)<br><br>Methods, subsection "exposure variables and software development" (p7&8)<br><br>N/A
Variables	7	Clearly define all outcomes, exposures, predictors, potential confounders, and effect modifiers. Give diagnostic criteria, if applicable.	Methods (p6-9)	RECORD 7.1: A complete list of codes and algorithms used to classify exposures, outcomes, confounders, and effect modifiers should be provided. If these cannot be reported, an explanation should be provided.	Methods (p6-9)
Data sources/ measurement	8	For each variable of interest, give sources of data and details of methods of assessment (measurement).<br>Describe comparability of assessment methods if there is more than one group	Methods (p6-9)		

For peer review only

Bias	9	Describe any efforts to address potential sources of bias	Methods, subsections "covariates" and "statistical analysis" (p8&9)		
Study size	10	Explain how the study size was arrived at	Methods, subsection "patient sample" (p6)		
Quantitative variables	11	Explain how quantitative variables were handled in the analyses. If applicable, describe which groupings were chosen, and why	Methods (p6-9)		
Statistical methods	12	(a) Describe all statistical methods, including those used to control for confounding<br>(b) Describe any methods used to examine subgroups and interactions<br>(c) Explain how missing data were addressed<br>(d) *Cohort study* - If applicable, explain how loss to follow-up was addressed<br>*Case-control study* - If applicable, explain how matching of cases and controls was addressed<br>*Cross-sectional study* - If applicable, describe analytical methods taking account of sampling strategy<br>(e) Describe any sensitivity analyses	Methods (p6-9)		
Data access and cleaning methods		..		RECORD 12.1: Authors should describe the extent to which the investigators had access to the database	Methods (p6-9)

For peer review only

				population used to create the study population.<br><br>RECORD 12.2: Authors should provide information on the data cleaning methods used in the study.	Methods (p6-9)
Linkage		..		RECORD 12.3: State whether the study included person-level, institutional-level, or other data linkage across two or more databases. The methods of linkage and methods of linkage quality evaluation should be provided.	N/A
**Results**					
Participants	13	(a) Report the numbers of individuals at each stage of the study (*e.g.*, numbers potentially eligible, examined for eligibility, confirmed eligible, included in the study, completing follow-up, and analysed)<br>(b) Give reasons for non-participation at each stage.<br>(c) Consider use of a flow diagram	Methods, subsection "patient sample" (p6)	RECORD 13.1: Describe in detail the selection of the persons included in the study (*i.e.,* study population selection) including filtering based on data quality, data availability and linkage. The selection of included persons can be described in the text and/or by means of the study flow diagram.	Methods, subsection "patient sample" (p6)
Descriptive data	14	(a) Give characteristics of study participants (*e.g.*, demographic, clinical, social) and information on exposures and potential confounders<br>(b) Indicate the number of participants with missing data for each variable of interest<br>(c) *Cohort study* - summarise follow-up time (*e.g.*, average and total amount)	Results (p10&11, table 1)		

For peer review only

Outcome data	15	*Cohort study* - Report numbers of outcome events or summary measures over time<br>*Case-control study* - Report numbers in each exposure category, or summary measures of exposure<br>*Cross-sectional study* - Report numbers of outcome events or summary measures	Results (p12&13, table 2)		
Main results	16	(a) Give unadjusted estimates and, if applicable, confounder-adjusted estimates and their precision (e.g., 95% confidence interval). Make clear which confounders were adjusted for and why they were included<br>(b) Report category boundaries when continuous variables were categorized<br>(c) If relevant, consider translating estimates of relative risk into absolute risk for a meaningful time period	Results (p14-19, tables 3-5)		
Other analyses	17	Report other analyses done—e.g., analyses of subgroups and interactions, and sensitivity analyses	Methods and Results (p6-19 - particularly subsection statistical analysis, p9; other tests also mentioned in Results where relevant)		
**Discussion**					
Key results	18	Summarise key results with reference to study objectives	Discussion (p20&21)		
Limitations	19	Discuss limitations of the study, taking into account sources of potential bias or imprecision.	Strengths and Limitations (p23-24)	RECORD 19.1: Discuss the implications of using data that were not created or collected to answer the specific research question(s). Include	Strengths and Limitations (p23-24)

For peer review only

		Discuss both direction and magnitude of any potential bias		discussion of misclassification bias, unmeasured confounding, missing data, and changing eligibility over time, as they pertain to the study being reported.	
Interpretation	20	Give a cautious overall interpretation of results considering objectives, limitations, multiplicity of analyses, results from similar studies, and other relevant evidence	Discussion (p20-23)		
Generalisability	21	Discuss the generalisability (external validity) of the study results	Strengths and Limitations (p23-24)		
**Other Information**					
Funding	22	Give the source of funding and the role of the funders for the present study and, if applicable, for the original study on which the present article is based	Funding and Conflict of Interest (p26)		
Accessibility of protocol, raw data, and programming code		..		RECORD 22.1: Authors should provide information on how to access any supplemental information such as the study protocol, raw data, or programming code.	Methods subsection "exposure variables and software Development" (p7): CRIS open-access online catalogue reference

*Reference: Benchimol EI, Smeeth L, Guttmann A, Harron K, Moher D, Petersen I, Sørensen HT, von Elm E, Langan SM, the RECORD Working Committee. The REporting of studies Conducted using Observational Routinely-collected health Data (RECORD) Statement. *PLoS Medicine* 2015; in press.

*Checklist is protected under Creative Commons Attribution (CC BY) license.

BMJ Open

Investigating the relationship between thought interference, somatic passivity and outcomes in patients with psychosis: a natural-language processing approach using the Clinical Record Interactive Search platform at the South London and Maudsley NHS Foundation Trust

Journal:	*BMJ Open*
Manuscript ID	bmjopen-2021-057433.R2
Article Type:	Original research
Date Submitted by the Author:	26-May-2022
Complete List of Authors:	Magrangeas, Thibault; King's College London Institute of Psychiatry Psychology and Neuroscience<br>Kolliakou, Anna; King's College London Institute of Psychiatry Psychology and Neuroscience, Institute of Psychiatry<br>Sanyal, Jyoti; South London and Maudsley NHS Foundation Trust<br>Patel, Rashmi; King's College London Institute of Psychiatry Psychology and Neuroscience; South London and Maudsley NHS Foundation Trust<br>Stewart, Robert; King's College London Institute of Psychiatry Psychology and Neuroscience; South London and Maudsley NHS Foundation Trust
**Primary Subject Heading**:	Mental health
Secondary Subject Heading:	Health informatics
Keywords:	Schizophrenia & psychotic disorders < PSYCHIATRY, Adult psychiatry < PSYCHIATRY, Health informatics < BIOTECHNOLOGY & BIOINFORMATICS

SCHOLARONE™
Manuscripts

I, the Submitting Author has the right to grant and does grant on behalf of all authors of the Work (as defined in the below author licence), an exclusive licence and/or a non-exclusive licence for contributions from authors who are: i) UK Crown employees; ii) where BMJ has agreed a CC-BY licence shall apply, and/or iii) in accordance with the terms applicable for US Federal Government officers or employees acting as part of their official duties; on a worldwide, perpetual, irrevocable, royalty-free basis to BMJ Publishing Group Ltd ("BMJ") its licensees and where the relevant Journal is co-owned by BMJ to the co-owners of the Journal, to publish the Work in this journal and any other BMJ products and to exploit all rights, as set out in our licence.

The Submitting Author accepts and understands that any supply made under these terms is made by BMJ to the Submitting Author unless you are acting as an employee on behalf of your employer or a postgraduate student of an affiliated institution which is paying any applicable article publishing charge ("APC") for Open Access articles. Where the Submitting Author wishes to make the Work available on an Open Access basis (and intends to pay the relevant APC), the terms of reuse of such Open Access shall be governed by a Creative Commons licence – details of these licences and which Creative Commons licence will apply to this Work are set out in our licence referred to above.

Other than as permitted in any relevant BMJ Author's Self Archiving Policies, I confirm this Work has not been accepted for publication elsewhere, is not being considered for publication elsewhere and does not duplicate material already published. I confirm all authors consent to publication of this Work and authorise the granting of this licence.

For peer review only

Title: Investigating the relationship between thought interference, somatic passivity and outcomes in patients with psychosis: a natural-language processing approach using the Clinical Record Interactive Search platform at the South London and Maudsley NHS Foundation Trust

Authors: Thibault Magrangeas[1], Anna Kolliakou[1], Jyoti Sanyal[2], Rashmi Patel[1&2], Robert Stewart[1&2]

Affiliations: 1: King's College London (Institute of Psychiatry, Psychology and Neuroscience), London, UK; 2: South London and Maudsley NHS Foundation Trust, London, UK

Corresponding Author: Thibault Magrangeas

Department of Psychological Medicine

Main Building E3.08

Institute of Psychiatry, Psychology and Neuroscience

King's College London

De Crespigny Park

London SE5 8AF

United Kingdom

Telephone: 00447761280858. **Fax:** N/A. **E-mail:** thibault.magrangeas@kcl.ac.uk c/o R Stewart's research group.

Abstract Word Count: 225

Body Word Count: 4933

Abstract

Objectives: We aimed to apply natural language-processing (NLP) algorithms in routine healthcare records to identify reported somatic passivity (external control of sensations, actions and impulses) and thought interference symptoms (thought broadcasting, insertion, withdrawal), first-Rank symptoms traditionally central to diagnosing schizophrenia, and determine associations with prognosis by analysing routine outcomes.

Design: Four algorithms were developed on de-identified mental healthcare data and applied to ascertain recorded symptoms over the three months following first presentation to a mental healthcare provider in a cohort of patients with a primary schizophreniform disorder (ICD-10 F20-F29) diagnosis.

Setting and participants: From the electronic health records of a large secondary mental healthcare provider in south London, 9,323 patients were ascertained from 2007 to the data extraction date (25/02/2020).

Outcomes: The primary binary dependent variable for logistic regression analyses was any negative outcome (Mental Health Act section, >2 antipsychotics prescribed, >22 days spent in crisis care) over the subsequent 2 years.

Results: Final adjusted models indicated significant associations of this composite outcome with baseline somatic passivity (prevalence 4.9%; adjusted odds ratio 1.61, 95% CI 1.37-1.88), thought insertion (10.7%; 1.24, 1.15-1.55) and thought withdrawal (4.9%; 1.36, 1.10-1.69), but not independently with thought broadcast (10.3%; 1.05, 0.91-1.22).

Conclusions: Symptoms traditionally central to the diagnosis of schizophrenia, but under-represented in current diagnostic frameworks, were thus identified as important predictors of short- to medium-term prognosis in schizophreniform disorders.

For peer review only - http://bmjopen.bmj.com/site/about/guidelines.xhtml

Keywords: Schizophrenia; Delusions; Prognosis; Psychosis; Patient Outcomes; Natural-Language Processing (NLP)

Strengths and limitations of this study

- This study was carried out on a very large patient sample and identified symptoms from routine care instead of specifically recruited and therefore less representative cohorts, and our applications achieved high-performance metrics.
- The patient sample was drawn from an inner urban area in South London, so results may not be entirely generalisable to the rest of the population.
- The recording of exposure variables relied on symptoms being elicited and noted by clinicians in routine practice and therefore cannot be assumed to be equivalent to those ascertained in a more research-focused interview, just as covariates such as diagnosis also were derived from routine clinical records and should not be assumed to equate research diagnostic criteria; however, we feel that inaccuracies in exposure ascertainment were more likely to obscure findings of interest through non-differential measurement error rather than give rise to spurious associations.
- Although multiple confounding factors were accounted for, our model-fit measures demonstrate model reliability but highlight residual unexplained variance possibly due to the influence of other psychotic symptoms as well as additional external factors which may affect outcomes and should be further investigated; our model is useful in correlating patient outcomes with symptoms at first presentation but it is not possible to infer stability of the clinical construct over time as the patient is only assessed once.
- Outcomes analysed were restricted to those most readily available from the source data and most likely to be clinically informative; however, they cannot be viewed as exhaustive and there may be elements of prognosis that were not captured in this study, including interactions between outcomes and previous adverse events that may influence symptom occurrence and recording.

For peer review only - http://bmjopen.bmj.com/site/about/guidelines.xhtml

INTRODUCTION

Schizophrenia was originally defined by Schneider around a core group of First-rank symptoms (FRS) which include auditory hallucinations (commonly characterised as but not limited to hearing one or multiple voices talking to or about the patient), delusions of perception (such as the sensation of being externally controlled) or thought interference (the sensation of having thoughts inserted, withdrawn or externally broadcasted against the patient's wishes) [1]. These symptoms were identified as central to diagnosing schizophrenia as they were observed in the majority of cases. Still today, recent studies have consistently found FRS in the form of thought interference and somatic passivity to be core symptoms of psychotic disorders in general and schizophrenia specifically[2-4]. A comparative study of thirteen different diagnostic systems identified a triad of positive symptoms strongly correlated with a schizophrenia diagnosis across all scales, the presence of two of which was sufficient to diagnose 78% of patients; these were auditory hallucinations, disturbances of affect and "passivity feelings" (defined as "any delusion of being influenced or interfered with by imaginary forces from outside, whether somatically or mentally" and therefore assumed to include thought interference)[5]; these symptoms were all already described in Bleuler's 1911 definition of schizophrenia[6], demonstrating historical consistency. Somatic passivity and thought interference have also been reported to be positively correlated and predict the occurrence of schizophrenia or schizoaffective disorder[7-8].

Despite this, ongoing research into the complex mechanisms underpinning psychosis manifestations has highlighted the wider range of symptoms that can occur and encouraged a new approach focused beyond these core FRS. For example, different symptoms have been observed to occur in clusters, such as somatic passivity and persecutory delusions[9] or auditory

verbal hallucinations[10]. Environmental factors also seem to influence symptom occurrence, leading to debate around the cross-cultural relevance of FRS[11]. In this context, recent changes to the DSM-5 including the removal of FRS from diagnostic criteria[12-13] have been welcomed as a valuable push towards a better understanding of individual symptoms; however, limitations remain as this new focus has been criticised for not fully addressing the relationship between symptoms; conflicting with most existing guidelines for early symptom detection in schizophrenia that still heavily rely on FRS; and diverting attention away from core psychopathological processes at the disorder's root[14].

Importantly, schizophrenia symptoms can directly influence both behaviour and consequently patient outcomes; for example, indirectly leading to violence through the occurrence of delusions[15]. FRS absence has been found to correlate with better outcomes, leading to a proposed redefinition of schizophrenia as a continuum, exhibiting a positive linear relationship between psychotic symptoms and outcomes[16-18]. Furthermore, the prevalences of individual symptoms appear to vary between early- and late-onset cases, reflecting complex interactions between symptoms and disease development[19-21]. This evidence suggests that patient outcomes are better in schizophrenia in the absence of FRS[17] but offers no insight into prognosis once present or into relationships between symptoms. While some authors have identified differing effects on outcomes, with passivity predicting a worsened prognosis in contrast to interference[17], others have highlighted a possible link, as both phenomenologically reflect a belief in external control, suggesting they may have a synergistic effect on outcomes[8,22-23] or even constitute components of the same delusion[10&24] or syndrome[25]. Overall, these studies highlight the significance of positive symptoms individually and as a group, suggesting possible correlations and reflecting the overlap in their occurrence, although the degree of overlap is rarely quantified.

For peer review only - http://bmjopen.bmj.com/site/about/guidelines.xhtml

The evidence highlighted here reflects the importance of clarifying the role of FRS in prognosis to understand which aspects of schizophrenia should be at the core of its diagnosis. Modern technologies have allowed the development of new tools allowing the analysis of large datasets using automated software. This is the goal of the South London and Maudsley (SLaM) NHS Foundation trust Clinical Record Interactive Search (CRIS) platform was developed in 2008 to enable research on a de-identified copy of the Trust's electronic health records (used across all services since 2006) within a robust governance and data security framework[24]. All the clinical notes of patients are recorded in a de-identified format on this database. Natural Language Processing (NLP) algorithms are then used to study these documents; these are algorithms that can be trained to recognise entities of interest and their context in free text, for example the mention of "thought interference" in a way that indicates that a patient is experiencing this symptom.

This study aimed to develop and evaluate four NLP algorithms to detect the recording of thought interference subcomponents (broadcasting, insertion, withdrawal) and somatic passivity across this database. Other algorithms had already been developed to capture the recorded occurrence of other important positive symptoms of psychosis (paranoia, auditory hallucinations, persecutory delusions) and were used to define potential covariates. We focused on individuals diagnosed with a schizophreniform disorder and analysed patient outcomes in this population. Our focus on thought interference and somatic passivity as particular symptoms aimed to clarify the interactions described previously which suggested a particularly strong relationship between these symptoms; in addition, they were chosen as potentially tractable to extraction at scale from routine mental healthcare data via NLP. Being described in relatively consistent language in clinical records, they made a pragmatic choice for further

investigation in contrast to other symptoms such as running commentary hallucinations which are typically described with greater linguistic heterogeneity (i.e., based on the individual experience of patients rather than a set vocabulary) and therefore harder to study with this approach.

For peer review only - http://bmjopen.bmj.com/site/about/guidelines.xhtml

METHODS

Context

We used data extracted from the electronic mental health clinical notes records of patients at the South London and Maudsley NHS Foundation Trust (SLaM), one of the largest mental health service providers in Europe, serving a geographic catchment of four South London boroughs (Southwark, Lambeth, Lewisham, Croydon) with around 1.3 million residents. The Trust provides care to 37,500 active patients, with 12,000 to 14,000 clinical events per week covering a diverse range of interventions in the community and in the inpatient setting[26&27]**.** SLaM's Clinical Record Interactive Search (CRIS) platform was developed in 2008 to enable research on a de-identified copy of the Trust's electronic health records (used across all services since 2006) within a robust governance and data security framework[24]. CRIS has been described in detail previously[28&29] and has supported over 250 research publications to date. Ethical approval as a data resource for secondary analysis in in place (Oxford Research Ethics Committee C, reference 18/SC/0372).

Patient sample

All patients aged 16-95 with a primary F20-F29 disorder diagnosis according to ICD-10 criteria recorded from 2007 to extraction date (25/02/2020) were included. This age-range was chosen to cover patients transitioning to adult mental health care, commonly occurring from age 16; patients with no recorded date of birth appear on the system as aged 100+ and represented all the individuals above age 95; they were therefore excluded to avoid skew when correcting for

age. According to these criteria, 9,536 patients were identified. Thought interference and somatic passivity occurrence in the three months following first presentation at SLaM (defined as first face-to-face contact) was sought. If multiple diagnoses were then recorded for the same patient, first diagnosis was used. This cut-off was chosen to allow time for clinicians to assess all patients and record their symptoms accurately and focus on patients presenting with these symptoms as first presentation rather than later development. Participants who died within this period were excluded. Of the initial 9,536 patients, 9,323 (97.8%) were included, 57.9% of whom were male. Mean age at inclusion was 39.9 years (SD=17.3).

Exposure variables and software development

CRIS data have been enhanced over the last 10 years by a range of NLP algorithms allowing a hitherto unavailable level of detail to be generated at scale from routine healthcare data, taking advantage of the level of detail recorded in extensive text fields. The algorithms, over 90 to date, are described individually in an open-access online catalogue[30], including detailed information on definitions and performance. A particular objective has been to provide, at scale, broader phenotypic information on people presenting with mental disorders than that supplied by clinician-assigned diagnostic codes, through the extraction of recorded symptoms with 60 algorithms to date developed to ascertain individual symptoms across psychotic and affective disorders[31]. Contributing to this wider initiative, and with the specific rationale in mind for this study, we developed four new algorithms to identify instances of recorded somatic passivity and thought insertion, withdrawal and broadcasting in CRIS using the TextHunter platform[32], a tool used to present short segments of text to an annotator in order to provide rapid large samples for the training of machine learning algorithms, as well as a platform for independent evaluation of algorithm output (figure 1).

The data analysed consisted of de-identified text from all inpatient and outpatient clinical notes and correspondence. The algorithms used were applied to text surrounding relevant keywords corresponding to each variable of interest; these keywords were defined by the authors to ensure detecting as wide a range of formulations as possible. For example, to detect free-text mentions of passivity delusions, the keyword "passiv*" was used, with the asterisk allowing for different formulations such as "passive" or "passivity". The documents identified were then manually annotated via the TextHunter platform to train the algorithm to automatically determine if this was relevant; for example, text stating that "the patient described passive external control of actions" would be marked as positive, text stating "the patient described a passive lifestyle" would be marked as irrelevant, and text stating “no passivity” would be marked as a negation statement. Once the algorithm had been optimized, independent evaluation took place on novel text to ascertain precision (proportion of algorithm-identified positive instances found to be correct on evaluation) and recall (proportion of positive entities identified as such by the algorithm) statistics. Testing the performance of the algorithm over the entire CRIS database was performed by checking 100 random annotated documents produced by the algorithm and 100 random unannotated documents using keyword searches. this constituted a gold-standard set from which a second researcher double-annotated a proportion of the retrieved documents to determine inter-rater reliability (IRR) using Cohen's kappa. The process was iterative and sought to achieve P and R values over 80%. High performance metrics were attained (supplementary table 1). For primary analyses, positive indications of the three symptoms representing thought interference (thought insertion, thought withdrawal, thought broadcast) were combined under that a single entity with secondary analyses considering them separately; all symptoms were defined as binary variables according to their recording or not in the first three months after first presentation.

Outcomes

Occurrence of negative outcomes was recorded over the two years following first presentation at SLaM. Three outcomes were evaluated: i) highest-quartile time spent as an inpatient or receiving support from a home treatment team in the community since first presentation (defined as 22 days or more); ii) receiving more than two antipsychotics; iii) involuntary psychiatric hospitalisation defined as detention for assessment or treatment under Sections 2 or 3 of the UK Mental Health Act (MHA).

Covariates

Included covariates were: gender, ethnic group, age at first SLaM presentation, diagnosis, Index of Multiple Deprivation (IMD) score and presence of recorded paranoia, persecutory delusions or auditory hallucinations. Age was categorised by decades for descriptive analyses but applied as a continuous variable otherwise. Diagnoses were recorded as a binary variable, distinguishing schizophrenia (ICD-10 F20x) from other schizophreniform disorders (F21-F29)[33]. The IMD score is a measure of neighbourhood deprivation, applied at Lower Super Output Area level (a standard national address code containing an average 1500 residents), and derived from composite national Census measures. This was divided in quartiles ranging from low (1) to high (4) deprivation. The presence of paranoia, auditory hallucinations, and persecutory delusions, three common psychotic symptoms in schizophrenia either previously suggested to strongly interact with passivity[20,34] or in the case of paranoia to constitute a syndrome distinct from that characterised by the presence of hallucinations or other delusions[24], was also ascertained for adjustment, using previously developed NLP algorithms[31].

For peer review only - http://bmjopen.bmj.com/site/about/guidelines.xhtml

Statistical analysis

All analyses were carried out using Stata 15.1 software. Initial descriptive analyses assessed overlap between symptoms, demographic/clinical factors and other psychotic symptoms. After describing the prevalence of outcomes by covariate, logistic regression models were assembled to investigate associations and their independence in relation to each binary outcome. Adjusted R-squared was used to assess model fit. Secondary analyses studied the impact of adjusting for covariates individually to clarify potential confounding effects. Finally, Poisson regression models investigated the relationship between number of negative outcomes and number of symptoms experienced as well as between number of negative outcomes and each individual symptom. Additional linear regression was carried out to study the relationship between symptoms and number of antipsychotics prescribed, assessing the reliability of the latter as a marker of negative outcome.

Patient and public involvement

The study proposal was reviewed and approved by the patient-led CRIS oversight committee prior to project commencement. No other consultations were made with patients or the public.

RESULTS

Table 1 describes symptom prevalences by demographic and clinical groups. Thought interference and somatic passivity were more common in younger patients and marginally higher in men compared to women but showed no consistent associations with ethnicity or neighbourhood deprivation. Both symptoms occurred more often in the presence of other psychotic symptoms but did not differ substantially between comparison diagnostic groups. Descriptive results indicated that the symptoms of interest overlapped. This was further analysed by describing the co-occurrence of symptoms and later analysing the correlation between this and negative outcomes. Overlaps between subcomponents of thought interference are summarised in Supplementary Figure 1, and Supplementary Figure 2 describes overlap between passivity, thought interference and the other positive symptoms included as covariates.

For peer review only - http://bmjopen.bmj.com/site/about/guidelines.xhtml

Table 1: symptom prevalence by demographic characteristics and diagnoses, and overlap with other psychotic symptoms

		None (%)	Interference without passivity (%)	*Broadcast (%)*	*Insertion (%)*	*Withdrawal (%)*	Passivity without interference (%)	Interference and passivity (%)	Total
	Total participants	77.0	12.8	10.3	10.7	4.9	4.9	5.2	9,323
Age at diagnosis	16-25	65.8	19.3	*16.1*	*17.4*	*7.8*	6.3	8.5	2,131
	26-35	72.4	14.8	*12.7*	*12.5*	*6.0*	5.9	6.9	2,457
	36-45	80.2	11.3	*8.6*	*8.7*	*4.4*	4.3	4.2	1,799
	46-55	82.8	10.2	*6.9*	*8.1*	*3.6*	3.8	3.2	1,241
	56-65	87.6	6.9	*4.1*	*4.8*	*2.0*	3.8	1.7	651
	66-75	91.0	4.8	*3.0*	*4.0*	*0.5*	2.6	1.6	568
	76-85	93.5	3.4	*2.6*	*1.6*	*1.0*	2.9	0.3	383
	86-95	92.5	6.5	*5.4*	*2.2*	*0.0*	1.1	0.0	93
Gender	Female	80.3	11.2	*8.5*	*10.2*	*3.8*	4.4	4.2	3,920
	Male	74.7	14.1	*11.5*	*11.1*	*5.7*	5.3	6.0	5,399
Ethnicity	Black	74.9	14.1	*11.2*	*11.7*	*5.5*	5.4	5.7	3,065
	South Asian	76.1	16.3	*12.0*	*11.6*	*7.3*	2.7	5.0	301
	Chinese	72.4	9.2	*10.5*	*7.9*	*2.6*	10.5	7.9	76
	Other Asian	72.7	13.3	*13.7*	*12.3*	*6.8*	3.8	10.2	293
	White	78.3	12.4	*10.1*	*10.3*	*4.4*	4.6	4.8	3,631
	Other/unknown	79.0	11.4	*8.3*	*9.6*	*4.3*	5.0	4.6	1,957
Index of Multiple Deprivation (IMD)	IMD quartile 1	78.1	12.7	*10.5*	*9.9*	*4.7*	4.4	4.8	2,331
	IMD quartile 2	77.5	12.1	*9.7*	*10.3*	*4.5*	5.1	5.3	2,331
	IMD quartile 3	75.7	13.1	*10.5*	*11.7*	*5.2*	5.3	5.8	2,338
	IMD quartile 4	76.8	13.4	*10.3*	*10.8*	*5.4*	4.8	5.0	2,323
Diagnosis	Schizophrenia diagnosis (F20)	78.2	12.0	*9.8*	*9.5*	*4.7*	4.9	4.9	3,475
	schizotypal/non-mood psychotic disorder (F21-29)	75.3	13.4	*10.5*	*11.4*	*5.0*	4.9	6.5	5,848
Other psychotic symptom	Paranoia present	73.2	15.0	*12.2*	*12.5*	*5.7*	5.7	6.2	7,478
	Paranoia absent	92.7	4.5	*2.5*	*3.5*	*1.8*	1.7	1.1	1,845
	Auditory Hallucinations present	65.8	18.4	*16.2*	*16.7*	*7.3*	6.9	9.0	4,971
	Auditory Hallucinations absent	89.9	6.5	*3.5*	*3.8*	*2.2*	2.6	1.0	4,352
	Persecutory Delusions present	66.2	18.1	*15.6*	*16.4*	*7.0*	7.3	8.4	4,375
	Persecutory Delusions absent	86.0	8.2	*5.6*	*5.6*	*3.1*	2.8	2.4	4984

Using logistic regression, odds ratios (OR) were calculated to further study overlap between symptoms (Supplementary Table 2), reiterating increased prevalence of thought interference and somatic passivity when other symptoms are present. Thought insertion and broadcasting were strongly correlated (OR=11.67) as were insertion and withdrawal (OR=12.10), whereas there was a weaker association between broadcast and withdrawal (OR=6.05). Somatic passivity was relatively strongly associated with broadcast (OR=5.13) and insertion (OR=6.66) but was more strongly associated with other psychotic symptoms such as paranoia (OR=4.67) and auditory hallucinations (OR=4.99) than with thought withdrawal (OR=3.86).

Table 2 describes outcome distributions. Younger patients, patients from Black ethnic groups and patients from higher deprivation neighbourhoods were at greater risk of negative outcomes. Patients with schizophrenia were less likely to have an MHA section or higher time in crisis care than those with other diagnoses but more likely to be prescribed multiple antipsychotics. Negative outcomes were higher in patients with reported paranoia, auditory hallucinations or persecutory delusions, and in those with thought interference or somatic passivity. As mentioned previously, based on past descriptions of symptoms as co-occurring we created Venn diagrams describing their overlap. Similarly, we investigated whether our outcomes of interest occurred independently or together. Supplementary Figure 3 describes overlap between outcomes.

For peer review only - http://bmjopen.bmj.com/site/about/guidelines.xhtml

Table 2: Two-year incidence of negative outcomes by symptom and covariate groups

	Covariate	Any negative Outcome (%)	MHA section (%)	>2 antipsychotics prescribed (%)	>22 days spent in crisis care (%)	Total participants
Interference/ Passivity	None	42.7	31.8	21.9	10.6	7,181
	Interference without passivity	61.4	46.5	31.6	32.6	1,198
	Broadcast	*63.3*	*47.4*	*34.2*	*34.9*	*956*
	Insertion	*67.4*	*49.9*	*36.6*	*38.1*	*997*
	Withdrawal	*66.9*	*52.1*	*36.2*	*40.1*	*459*
	Passivity without interference	68.2	49.8	36.4	35.7	456
	Interference and passivity	72.3	54.5	42.2	43.2	488
	Total participants	47.9	35.8	24.9	25.0	9,323
Age at diagnosis	16-25	61.0	48.6	32.7	36.8	2,131
	26-35	52.8	40.7	27.0	26.1	2,457
	36-45	55.3	32.9	22.5	21.2	1,799
	46-55	54.1	31.7	23.8	20.9	1,241
	56-65	33.5	21.8	17.8	16.9	651
	66-75	29.9	17.8	15.7	16.2	568
	76-85	24.3	15.4	11.7	13.3	383
	86-95	24.7	14.0	14.0	12.9	93
Gender	Female	46.9	35.2	25.1	24.2	3,920
	Male	48.6	36.2	24.8	25.6	5,399
Ethnicity	Black	57.5	47.6	30.0	31.3	3,065
	South Asian	43.5	30.2	24.9	19.3	301
	Chinese	50.0	44.7	15.8	21.1	76
	Other Asian	48.1	36.2	27.3	23.9	293
	White	43.9	31.1	23.3	22.1	3,631
	Other/unknown	40.7	26.4	20.0	21.7	1,957
Index of Multiple Deprivation (IMD)	IMD quartile 1	45.6	32.9	24.5	23.1	2,331
	IMD quartile 2	46.3	33.6	25.0	24.2	2,331
	IMD quartile 3	50.3	38.1	25.8	26.6	2,338
	IMD quartile 4	49.2	38.4	24.3	26.1	2,323
Diagnosis	Schizophrenia diagnosis (F20)	44.4	30.6	29.9	22.2	3,475
	schizotypal/non-mood psychotic disorder (F21-29)	49.9	38.8	22.0	26.7	5,848
Other symptoms	Paranoia present	53.9	41.0	27.7	28.9	7,478
	Paranoia absent	23.6	14.5	13.5	9.4	1,845
	Auditory Hallucinations present	58.2	43.8	31.3	31.7	4,971
	Auditory Hallucinations absent	36.0	26.6	17.6	17.3	4,352
	Persecutory Delusions present	62.2	49.2	31.9	34.4	4,375
	Persecutory Delusions absent	35.7	23.7	18.6	16.6	4984

Table 3 summarises logistic regression analyses for any negative outcome. Unadjusted models showed significant associations with somatic passivity and all components of thought interference, and these remained significant after adjustments except thought broadcast. Although confidence intervals overlapped, associations with somatic passivity were consistently strongest. Post-hoc investigations of those with non-overlapping somatic passivity and thought interference showed a significant association with somatic passivity (OR=1.57, 95% CI 1.27-1.95) but not with thought interference (OR=1.11, 95% CI 0.97-1.27).

For peer review only - http://bmjopen.bmj.com/site/about/guidelines.xhtml

Table 3: Unadjusted and adjusted odds ratio for the occurrence of any negative outcome in the presence of thought interference and somatic passivity

Outcome: any negative outcome	**Unadjusted OR (95% CI)**	**Adjusted* OR (95% CI)**	**Adjusted model pseudo R-squared**
Somatic passivity	2.86 (2.47-3.31)	1.61 (1.37-1.88)	0.12
Interference	2.30 (2.07-2.57)	1.25 (1.11-1.41)	0.12
Broadcast	2.01 (1.75-2.31)	1.05 (0.91-1.22)	0.12
Insertion	2.47 (2.15-2.84)	1.24 (1.15-1.55)	0.12
Withdrawal	2.29 (1.88-2.79)	1.36 (1.10-1.69)	0.12
Interference or passivity	2.54 (2.29-2.80)	1.30 (1.19-1.42)	0.12
Both interference and passivity	1.73 (1.57-1.92)	1.24 (1.11-1.38)	0.12
Post-hoc: passivity without interference	2.44 (1.99-2.98)	1.57 (1.27-1.95)	0.12
Post-hoc: interference without passivity	1.88 (1.66-2.13)	1.11 (0.97-1.27)	0.12

*Adjusted for age at diagnosis, gender, ethnicity, diagnosis, IMD quartile and presence of paranoia, auditory hallucinations, persecutory delusion.
Abbreviations: OR = odds ratio; RR = risk ratio

Table 4 displays further analyses of individual outcomes. Associations with somatic passivity remained significant for all after adjustments. Thought interference and insertion specifically were independently associated with higher time spent in crisis care and greater numbers of antipsychotics used, but not with MHA sections. Thought broadcast specifically was not independently associated with any outcome, and thought withdrawal was only associated with higher time spent in crisis care and MHA sections. Aside from thought withdrawal, all other symptoms had the weakest association with MHA sections.

For peer review only - http://bmjopen.bmj.com/site/about/guidelines.xhtml

Table 4: Unadjusted and adjusted odds ratios for associations of each negative outcome with the presence of somatic passivity, thought interference, broadcasting, insertion and withdrawal

**Exposure: somatic passivity**	**Unadjusted OR (95% CI)**	**Adjusted* OR (95% CI)**	**Adjusted model pseudo R-squared**
Any negative outcome	2.86 (2.47-3.31)	1.61 (1.37-1.88)	0.12
>22 days spent in crisis care	2.15 (1.87-2.48)	1.33 (1.15-1.54)	0.08
MHA section	2.13 (1.86-2.44)	1.23 (1.06-1.42)	0.13
>2 antipsychotics prescribed	2.14 (1.86-2.46)	1.43 (1.23-1.66)	0.07
**Exposure: thought interference**			
Any negative outcome	2.30 (2.07-2.57)	1.25 (1.11-1.41)	0.12
>22 days spent in crisis care	1.89 (1.69-2.12)	1.13 (1.00-1.28)	0.08
MHA section	1.95 (1.75-2.17)	1.08 (0.96-1.21)	0.13
>2 antipsychotics prescribed	1.80 (1.60-2.01)	1.16 (1.02-1.31)	0.07
**Exposure: thought broadcasting**			
Any negative outcome	2.01 (1.75-2.31)	1.05 (0.91-1.22)	0.12
>22 days spent in crisis care	1.71 (1.49-1.97)	1.01 (0.87-1.18)	0.08
MHA section	1.71 (1.50-1.96)	0.92 (0.80-1.07)	0.13
>2 antipsychotics prescribed	1.66 (1.44-1.91)	1.05 (0.90-1.22)	0.07
**Exposure: thought insertion**			
Any negative outcome	2.47 (2.15-2.84)	1.34 (1.15-1.56)	0.12
>22 days spent in crisis care	2.01 (1.75-2.31)	1.21 (1.05-1.40)	0.08
MHA section	1.93 (1.69-2.20)	1.06 (0.92-1.23)	0.13
>2 antipsychotics prescribed	1.88 (1.64-2.16)	1.22 (1.05-1.41)	0.07
**Exposure: thought withdrawal**			
Any negative outcome	2.29 (1.88-2.79)	1.36 (1.10-1.69)	0.12
>22 days spent in crisis care	2.09 (1.73-2.54)	1.37 (1.12-1.68)	0.08
MHA section	2.02 (1.68-2.44)	1.23 (1.01-1.51)	0.13
>2 antipsychotics prescribed	1.76 (1.45-2.14)	1.21 (0.98-1.48)	0.07

*Adjusted for age at diagnosis, gender, ethnicity, diagnosis, IMD quartile and presence of paranoia, auditory hallucinations, persecutory delusion.
Abbreviations: OR = odds ratio; MHA = Mental Health act

Table 5 summarises analyses investigating adjustments by individual covariates. For both somatic passivity and thought interference, strongest reductions in OR occurred following adjustments for age and the other three psychotic symptoms; however, OR for somatic passivity remained stronger throughout. Estimated marginal means (EM) were calculated to determine the likelihood of negative outcomes at different ages (Supplementary Table 3); this showed a decrease in risk inversely proportional to age: for a patient with somatic passivity, at age 20 EM=59.6(58.0-61.1)%; at age 90, EM=21.5(19.2-23.8)%. For a patient with thought interference, at age 20 EM=59.1(57.6-60.7)%; at age 90, EM=22.2(19.8-24.6)%. Finally, exploratory testing investigating the cumulative relationship between symptoms and outcomes, calculating the odds of experiencing multiple negative outcomes in relation to the sum of individual symptoms and to each individual symptom and illustrated using EM, described a 14.9% increase in the risk of multiple negative outcomes when experiencing no versus all four symptoms, from 73.1% to 88.0%. Consistently with identified trends, somatic passivity was the individual symptom with the greatest impact (adjusted OR=1.16(1.09-1.23)) and thought broadcasting had no significant effect after adjustment (OR=0.99(0.92-1.05)). Regression analysis identified a significant interaction between somatic passivity and number of antipsychotics ($p<0.05$; adjusted R-squared=0.1031). Thought interference and its subcomponents, also included in the model, had no significant effect (supplementary table 4).

For peer review only - http://bmjopen.bmj.com/site/about/guidelines.xhtml

Table 5: Logistic regression analysis of associations with any negative outcome adjusting separately for individual covariates

Outcome: Any negative outcome	**Somatic Passivity**	**Thought Interference**
Unadjusted	2.86 (2.47-3.31)	2.30 (2.07-2.57)
Adjusted for age at diagnosis	2.47 (2.13-2.86)	1.94 (1.73-2.17)
Adjusted for gender	2.85 (2.46-3.30)	2.30 (2.06-2.57)
Adjusted for ethnicity	2.85 (2.46-3.30)	2.28 (2.04-2.55)
Adjusted diagnosis	2.86 (2.47-3.31)	2.30 (2.06-2.56)
Adjusted for IMD quartile	2.86 (2.47-3.31)	2.30 (2.06-2.57)
Adjusted paranoia	2.43 (2.09-2.82)	1.94 (1.74-2.17)
Adjusted for auditory hallucinations	2.23 (1.92-2.59)	1.80 (1.61-2.02)
Adjusted for persecutory delusions	2.23 (1.91-2.59)	1.82 (1.63-2.04)

For peer review only - http://bmjopen.bmj.com/site/about/guidelines.xhtml

DISCUSSION

Having developed NLP algorithms to extract recorded thought interference and somatic passivity in routine electronic mental health records, we investigated their associations with negative outcomes over a 2-year period following first-recorded diagnosis of schizophrenia or a schizophreniform disorder. Both symptoms were associated with a significantly increased risk of experiencing negative outcomes, somatic passivity having the strongest associations after full adjustment. This is broadly consistent with previous reports that experiencing these symptoms is predictive of negative outcomes: Stephen et al found the absence of somatic passivity to correlate with better outcomes in 66% of patients[8]; Koehler found no significant difference in outcomes between patients experiencing any thought interference subcomponent or somatic passivity but concluded that their absence was predictive of a better prognosis[9]. In 2019, Malinowski et al found that thought broadcasting seemed predictive of disease continuation without remission, but that this effect was no longer significant after adjusting for external factor influence (specifically, untreated psychosis duration and drug abuse)[35]; accordingly, although different covariates were included, our results suggest that thought broadcast does not have a significant effect on outcome. Similar prevalence and overlap between thought insertion, withdrawal and broadcasting suggests that this cannot be due to broadcasting occurring more frequently in isolation. It is interesting to note that aside from broadcasting, these symptoms remained significantly associated with negative outcomes after including common non first-rank symptoms of schizophrenia in our covariates, namely paranoia, auditory hallucinations and persecutory delusions, suggesting the importance of their link with negative outcomes. Secondary testing identified a unique significant effect of somatic passivity on number of antipsychotics prescribed, possibly reflecting poorer response leading to worsened outcomes; importantly, this outcome may reflect a degree of treatment resistance

For peer review only - http://bmjopen.bmj.com/site/about/guidelines.xhtml

not investigated here but likely to influence patient prognosis. Our findings suggest that somatic passivity and thought interference subcomponents correlate with an increased risk of experiencing negative outcomes, some more strongly than others. In contrast, it is interesting to note that no particular outcome appeared to be disproportionately correlated with symptoms. Although the odds ratio of an MHA section being in place were less strong than other outcomes, this reflects that their use is reserved for the most unwell patients who refuse or lack capacity to accept voluntary community treatment. Overall, the odds of all three outcomes increased in a similar fashion in the presence of thought interference and somatic passivity, reflecting a generally worsened prognosis.

Nuevo et al described schizophrenia symptoms as a continuum whereby the experience of multiple symptoms predicts a worse prognosis[10]. Our finding that patients with either or both thought interference and somatic passivity were more likely to experience negative outcomes than those for whom these symptoms were absent supports this. Exploratory analyses suggested a significant relationship between number of symptoms experienced and negative outcome occurrence; however, our results also suggest that each symptom has a different impact on outcomes which was not always individually significant, and that the presence of multiple symptoms may even sometimes reduce the frequency of negative outcomes: for example, patients experiencing somatic passivity only may have poorer outcomes than those with both this and thought interference. One explanation may be that reporting thought interference to clinicians requires greater insight from the patient, contributing to better outcomes and facilitating voluntary treatment such as that offered by crisis care teams with which more patients experiencing thought insertion and withdrawal engaged. The recording of exposure variables relied on symptoms being elicited and noted by clinicians in routine practice and therefore cannot be assumed to be equivalent to those ascertained in a more research-

For peer review only - http://bmjopen.bmj.com/site/about/guidelines.xhtml

focused interview, just as covariates such as diagnosis also were derived from routine clinical records and should not be assumed to equate research diagnostic criteria; however, we feel that inaccuracies in exposure ascertainment were more likely to obscure findings of interest through non-differential measurement error rather than give rise to spurious associations. Unfortunately, although mandatory hospitalisation under the MHA reflects a need for crisis care, it is not possible with these algorithms to determine what interventions patients received and responded to in that context specifically. However, guideline consistency since 2007 suggests that patients represented in this analysis should have received similar standards of care across the time period sampled. Overall, although there does seem to be a trend of worsening outcomes as symptoms accumulate, it would be overly simplistic to claim that this effect is purely additive.

Interestingly, of the covariates investigated, only age had a substantial confounding effect, negatively correlated with symptoms and outcomes of interest. In the past, thought insertion and withdrawal have been reported as more common in early-onset cases and somatic passivity as more frequent in late-onset cases[11]. Because of challenges in establishing whether patients were previously diagnosed in another service before presenting at SLaM, it is not possible to equate first presentation here with first-episode psychosis, particularly in older individuals who may have longer standing diagnoses and recently in-migrated to the catchment. This first presentation sample is therefore better viewed as one which is enriched with first-episode cases rather than directly equivalent. Furthermore, as our database consists of real-life data patients do not always have consistent follow-up or repeat assessments, including due to external factors not related to the patient's care. Our model is therefore useful in correlating patient outcomes with symptoms at first presentation but it is not possible to infer stability of the clinical construct over time as the patient is only assessed once. Although age at diagnosis may

For peer review only - http://bmjopen.bmj.com/site/about/guidelines.xhtml

not accurately reflect age at disease onset and doesn't account for possible changes in symptom profile as patients age, our results suggest that both symptoms are more frequent in younger patients, leading to a worsened prognosis. In this context, it would be interesting, in future research, to investigate symptom stability over time and how prognosis might also change with this.

Other external factors such as level of education or spiritual beliefs may play a role in the personal interpretation of abnormal experiences and have consequently affected outcomes. We developed a model that suggested the existence of significant interactions between thought interference, somatic passivity and the occurrence of negative outcomes. As shown in supplementary figures 1 and 2, these symptoms overlapped in many patients as well as with other psychotic symptoms, and so did the negative outcomes under scrutiny. The overlap observed before further analyses suggested that there may exist interactions linking these factors and directed our analyses towards the development of a model to understand these better. The algorithms we developed to create this model achieved high performance metrics and our model-fit measures demonstrate model reliability but highlight that the variance remains in great part unexplained, likely due to the role of other factors not included here. This could be usefully explored in further studies to refine models assessing patient prognosis. Furthermore, our patient sample was drawn from an inner-urban South-London population and results may be different in other patient groups, although the large sample size should mitigate this effect.

Paranoia, persecutory delusions and auditory hallucinations were included as covariates due to past evidence suggesting a correlation with thought interference and somatic passivity. The primary purpose of this study was to characterise symptoms of thought interference and

For peer review only - http://bmjopen.bmj.com/site/about/guidelines.xhtml

passivity, and other symptoms, such as negative and disorganised symptoms, were not included. Broader symptom profiles should be investigated in future research. Interactions between outcomes should also be studied; for example, it is not unreasonable to propose that the prescription of fewer antipsychotics may correlate with reduced hospitalisation time. To this effect, a harmonisation of definitions would be useful to enable the development of algorithms studying symptoms described more heterogeneously. Outcomes analysed were restricted to those most readily available from the source data and most likely to be clinically informative; however, they cannot be viewed as exhaustive and there may be elements of prognosis that were not captured in this study, including interactions between outcomes and previous adverse events that may influence symptom occurrence and recording.

Although to be treated with caution, as not tested for significance, our descriptive results provide potential directions for future research and support the notion that psychotic symptoms share more complex interactions than previously described. Somatic passivity has been suggested to cluster with different symptoms such as persecutory delusions[4] or auditory-verbal hallucinations[5], and thought interference has sometimes been described as a component of somatic passivity[18&36]. We found that subcomponents of thought interference traditionally assumed to closely correlate may actually more commonly occur alongside other symptoms; e.g., the odds of experiencing thought insertion alongside somatic passivity were greater than those of withdrawal and broadcasting co-occurring. In a study attempting to link delusions to putative underlying neurobiological mechanisms, Kimhy et al called for new symptom definitions that would focus on underlying mechanisms rather than phenomenological descriptions[23]. Each delusion was described then grouped with others based on correlation, enabling the grouping of symptoms that share significant interactions while maintaining their individuality. This approach enables a less biased study of psychotic symptoms relying less on

For peer review only - http://bmjopen.bmj.com/site/about/guidelines.xhtml

traditional groupings and may allow more accurate descriptions of symptom interactions. Additionally, thought interference and somatic passivity have previously been found to be closely related and predictive of schizophrenia[13&36-38]. Our results support reports that these symptoms share a positive correlation[20] but question their specificity to schizophrenia and usefulness in differentiating this from other psychotic disorders, particularly as both were absent in 77% of patients. Somatic passivity has been reported to be the most common symptom experienced in schizophrenia[6] and first-episode psychosis[24]; however, in our study thought interference was almost twice as commonly recorded. Overall, it would be interesting to repeat these analyses in the future with a broader range of diagnoses including affective psychoses in which they also occur to determine whether these symptoms could be more specific to other conditions or used to assess patient prognosis.

CONCLUSION

This study developed NLP applications to detect the recorded presence of somatic passivity, thought interference and its subcomponents in patients within three months of their first presentation and analysed negative outcome occurrence over the two following years to investigate the relationship between these specific FRS and patient prognosis. We observed a significant effect of somatic passivity, thought interference and its subcomponents thought insertion and withdrawal on the likelihood of experiencing any negative outcome. We also offer evidence that psychotic symptoms traditionally associated with schizophrenia may not be specific to this disorder and share complex interactions, putting in question current definitions and warranting further investigation to determine whether it is correct to consider thought insertion, withdrawal and broadcasting as parts of the same symptom. Overall, the presence of somatic passivity and thought interference was found to significantly increase the risk of experiencing negative outcomes in psychosis; somatic passivity had the greatest effect. Future research should focus on acquiring a better understanding of these effects, including by investigating other psychotic symptoms to determine the more detailed pathways through which these interact with prognosis in schizophrenia and other types of psychosis. Future studies should investigate these correlations and reliable markers of outcomes to develop standardised tools enabling a better evaluation of patient prognosis. In addition, it would be interesting to study which antipsychotics specifically were prescribed and whether patients may have had a better response to specific medication, reflective of a general lack of response to individual antipsychotics in schizophrenia rather than the presence of refractory disease.

For peer review only - http://bmjopen.bmj.com/site/about/guidelines.xhtml

Funding

RS, RP and AK are part-funded by the National Institute for Health Research (NIHR) Biomedical Research Centre at the South London and Maudsley NHS Foundation Trust and King's College London. RS is additionally part-funded by: i) an NIHR Senior Investigator Award; ii) the National Institute for Health Research (NIHR) Applied Research Collaboration South London (NIHR ARC South London) at King's College Hospital NHS Foundation Trust; iv) the DATAMIND HDR UK Mental Health Data Hub (MRC grant MR/W014386). The views expressed are those of the authors and not necessarily those of the NIHR or the Department of Health and Social Care. RP has received funding from an NIHR Advanced Fellowship (NIHR301690), a Medical Research Council (MRC) Health Data Research UK Fellowship (MR/S003118/1) and a Starter Grant for Clinical Lecturers (SGL015/1020) supported by the Academy of Medical Sciences, The Wellcome Trust, MRC, British Heart Foundation, Arthritis Research UK, the Royal College of Physicians and Diabetes UK.

Conflict of Interest

RS declares research support received in the last 36 months from Janssen, GSK and Takeda. RP has received grant funds from Janssen and consultancy fees from Induction Healthcare and Holmusk.

Author Statement

TM, AK, JS, RP and RS planned the study together. JS developed the applications, which were doubly annotated by TM and AK. JS and AK retrieved the data, and TM analysed it with advice from AK, JS, RP and RS. TM wrote the largest part of the paper, produced the tables and figures and submitted it, working under supervision from AK and RS. AK, RP and RS commented on and directly contributed to the manuscript.

For peer review only - http://bmjopen.bmj.com/site/about/guidelines.xhtml

Ethics Statement

The source de-identified data are made available for secondary analyses as approved by Oxford Research Ethics Committee C, reference 18/SC/0372.

Data availability statement

No additional data are available.

Figure Legends

Figure 1: Flowchart illustrating the development of algorithms via the TextHunter platform. TextHunter allows a training dataset of text strings containing wording of interest to be presented in an easy visual form for researcher annotation. The development process is identical for all algorithms relating to symptoms recorded in clinical text.

For peer review only - http://bmjopen.bmj.com/site/about/guidelines.xhtml

REFERENCES

1. Schneider, K. *Klinische Psychopathologie* (5th edn) (Trans. Hamilton). New York: Grune & Stratton; 1959.
2. Chapman LJ, Chapman JP. Scales for Rating Psychotic and Psychotic-like Experiences as Continua. *Schizophrenia Bull*. 1980;6(3):476-489 doi: 10.1093/schbul/6.3.476 [published in print First: January 1980]
3. Chapman LJ, Edell WS, Chapman JP. Physical Anhedonia, Perceptual Aberration, and Psychosis Proneness. *Schizophrenia Bull*. 1980;6(4):639-653 doi: 10.1093/schbul/6.3.476 [published in print First: January 1980]
4. Kwapil TR, Chapman LJ, Chapman J. Validity and Usefulness of the Wisconsin Manual for Assessing Psychotic-like Experiences. *Schizophrenia Bull*. 1999;25(2):363-375 doi: 10.1093/oxfordjournals.schbul.a033384 [published in print First: 1999]
5. Landmark J, Merskey H, Cernovsky Z, et al. The positive triad of schizophrenic symptoms. Its statistical properties and its relationship to 13 traditional diagnostic systems. *Br J Psychiatry J Ment Sci*. 1990;156(3):388-394. doi: 10.1192/bjp.156.3.388 [published Online First: July 2011]
6. Bleuler, E. *Dementia Praecox oder Gruppe der Schizophrenien*. Leipzig: Germany Deuticke; 1911.
7. Cloninger CR, Martin RL, Guze SB, et al. Diagnosis and Prognosis in Schizophrenia. *Arch Gen Psychiat*. 1985;42(1):15 doi: 10.1001/archpsyc.1985.01790240017002 [published in print First: January 1985]
8. Loftus J, Delisi LE, Crow TJ. Factor structure and familiality of first-rank symptoms in sibling pairs with schizophrenia and schizoaffective disorder. *Brit J Psychiat*. 2000;177(1):15-19 doi: 10.1192/bjp.177.1.15 [published Online First: July 2000]

For peer review only - http://bmjopen.bmj.com/site/about/guidelines.xhtml

9. Fierro M, Hernández AM, Malcolm J. The Psychopathology of Delusion of Control According to Subjective Experience. *Revista Colombiana De Psiquiatría*. 2018;47(4):221-228 doi:10.1016/j.rcpeng.2018.10.005 [published Online First: December 2018]

10. Rosen C, Jones N, Chase KA, et al. The intrasubjectivity of self, voices and delusions: A phenomenological analysis. *Psychos*. 2016;8(4):357-368 doi: 10.1080/17522439.2016.1162839 [published Online First: April 2016]

11. Malik SB, Ahmed M, Bashir A, et al. Schneider's First-Rank Symptoms of Schizophrenia: Prevalence and Diagnostic Use. *Brit J Psychiat*. 1990;156(1):109-111 doi: 10.1192/bjp.156.1.109 [published in print First: January 1990]

12. American Psychiatric Association. *Diagnostic and Statistical Manual of Mental Disorders, Fifth Edition (DSM-5)*. Washington, DC: American Psychiatric Association; 2013.

13. Kendler KS. Phenomenology of Schizophrenia and the Representativeness of Modern Diagnostic Criteria. *Jama Psychiat*. 2016;73(10):1082 doi:10.1001/jamapsychiatry.2016.1976 [published Online First: October 2016]

14. Nemeroff CB, Weinberger D, Rutter M, et al. DSM-5: a collection of psychiatrist views on the changes, controversies, and future directions. *Bmc Med*. 2013;11(1):202 doi: 10.1186/1741-7015-11-202 [published Online First: September 2013]

15. Ullrich S, Keers R, Coid JW. Delusions, anger, and serious violence: new findings from the MacArthur Violence Risk Assessment Study. *Schizophrenia Bull*. 2013;40(5):1174-1181 doi: doi.org/10.1093/schbul/sbt126 [published Online First: September 2013]

16. Stephens JH, Richard P, McHugh PR. Long-Term Follow-up of Patients Hospitalized for Schizophrenia, 1913 to 1940. *J Nerv Amp Ment Dis*. 1997;185(12):715-721.

17. Koehler K. Prognostic Prediction in RDC Schizo-Affective Disorder on the Basis of First-Rank Symptoms Weighted in Terms of Outcome. *Psychopathology*. 1983;16(2-4):186-197 doi: 10.1159/000283967 [published in print First: 1983]

For peer review only - http://bmjopen.bmj.com/site/about/guidelines.xhtml

18. Nuevo R, Chatterji S, Verdes E, et al. The continuum of psychotic symptoms in the general population: a cross-national study. *Schizophrenia Bull*. 2010;38(3):475-485 doi: 10.1093/schbul/sbq099 [published Online First: September 2010]

19. Howard R, Castle D, Wessely S, et al. A Comparative Study of 470 Cases of Early-Onset and Late-Onset Schizophrenia. *Brit J Psychiat*. 1993;163(3):352-357 doi: 10.1192/bjp.163.3.352 [published in print First: September 1983]

20. Alici-Evcimen Y, Ertan T, Eker E. Case Series With Late-Onset Psychosis Hospitalized in a Geriatric Psychiatry Unit in Turkey: Experience in 9 Years. *Int Psychogeriatr*. 2003;15(1):69-72 doi: 10.1017/S1041610203008767 [published Online First: March 2003]

21. Naqvi HA, Hussain S, Islam M, et al. Early psychosis symptoms. *J Coll Physicians Surg Jcpsp*. 2014;24(3):198-202.

22. Stanghellini G, Monti MR. Influencing and being influenced: the other side of "bizarre delusions". 2. Clinical investigation. *Psychopathology*. 1993;26(3-4):165-169 doi: 10.1159/000284817 [published in print First: February 1993]

23. Kimhy D, Goetz R, Yale S, et al. Delusions in Individuals with Schizophrenia: Factor Structure, Clinical Correlates, and Putative Neurobiology. *Psychopathology*. 2005;38(6):338-344 doi: 10.1159/000089455 [published Online First: December 2005]

24. Fernandes AC, Cloete D, Broadbent MTM, et al. Development and evaluation of a de-identification procedure for a case register sourced from mental health electronic records. *Bmc Med Inform Decis*. 2013;13(1):71 doi: 10.1186/1472-6947-13-71 [published Online First: July 2013]

25. Stuart GW, Malone V, Currie J, et al. Positive and negative symptoms in neuroleptic-free psychotic inpatients. *Schizophr Res*. 1995;16(3):175-188 doi: 10.1016/0920-9964(94)00083-K [published in print First: August 1995]

For peer review only - http://bmjopen.bmj.com/site/about/guidelines.xhtml

26. Patel R, Irving J, Brinn A, *et al.* Impact of the COVID-19 pandemic on remote mental healthcare and prescribing in psychiatry: an electronic health record study. *Bmj Open* 2021;**11**:e046365. doi:10.1136/bmjopen-2020-046365.
27. South London and Maudsley NHS Foundation Trust. Our Services, Types of service. https://slam.nhs.uk/our-services/types-of-service/ (accessed 21 Feb. 2022).
28. Stewart R, Soremekun M, Perera G, et al. The South London and Maudsley NHS Foundation Trust Biomedical Research Centre (SLAM BRC) case register: development and descriptive data. *Bmc Psychiatry*. 2009;9(1):51 doi: 10.1186/1471-244X-9-51 [published Online First: August 2009]
29. Perera G, Broadbent M, Callard F, et al. Cohort profile of the South London and Maudsley NHS Foundation Trust Biomedical Research Centre (SLaM BRC) Case Register: current status and recent enhancement of an Electronic Mental Health Record-derived data resource. *Bmj Open*. 2016;6(3):e008721 doi: 10.1136/bmjopen-2015-008721 [published Online First: March 2016]
30. CRIS NLP Service. CRIS Natural Language Processing Applications Library (2020), v1.2. Maudsley Biomedical Research Centre. https://www.maudsleybrc.nihr.ac.uk/facilities/clinical-record-interactive-search-cris/cris-natural-language-processing/. Updated 12/06/20. Accessed 03/08/20.
31. Jackson RG, Patel R, Jayatilleke N, et al. Natural language processing to extract symptoms of severe mental illness from clinical text: the Clinical Record Interactive Search Comprehensive Data Extraction (CRIS-CODE) project. *Bmj Open*. 2017;7(1):e012012 doi: 10.1136/bmjopen-2016-012012 [published Online First: January 2017]
32. Jackson RG, Ball M, Patel R, et al. TextHunter--A User Friendly Tool for Extracting Generic Concepts from Free Text in Clinical Research. *Amia Annu Symposium Proc Amia*

For peer review only - http://bmjopen.bmj.com/site/about/guidelines.xhtml

Symposium Amia Symposium. 2014;2014:729-738 doi: 10.13140/2.1.3722.9121 [published Online First: November 2014]

33. World Health Organisation. *International Statistical Classification of Diseases and Related Health Problems, 10th Revision (ICD-10)*. Geneva: WHO; 1992.
34. Galdos P, Os J van. Gender, psychopathology, and development: from puberty to early adulthood. *Schizophr Res*. 1995;14(2):105-112 doi: 10.1016/0920-9964(94)00020-9 [published in print First: January 1995]
35. Malinowski FR, Tasso B de C, Ortiz BB, et al. Schneider's first-rank symptoms as predictors of remission in antipsychotic-naive first-episode psychosis. *Revista Brasileira De Psiquiatria Sao Paulo Braz 1999*. 2019;42(AHEAD):22-26 doi: 10.1590/1516-4446-2018-0237 [published Online First: February 2020]
36. Klosterkötter J, Hellmich M, Steinmeyer EM, et al. Diagnosing Schizophrenia in the Initial Prodromal Phase. *Arch Gen Psychiat*. 2001;58(2):158 doi: 10.1001/archpsyc.58.2.158 [published Online First: February 2001]
37. Moller P, Husby R. The Initial Prodrome in Schizophrenia: Searching for Naturalistic Core Dimensions of Experience and Behavior. *Schizophrenia Bull*. 2000;26(1):217-232 doi: 10.1093/oxfordjournals.schbul.a033442 [published Online First: January 2000]
38. Galdos P, Os J van. Gender, psychopathology, and development: from puberty to early adulthood. *Schizophr Res*. 1995;14(2):105-112 doi: 10.1016/0920-9964(94)00020-9 [published in print First: January 1995]

For peer review only - http://bmjopen.bmj.com/site/about/guidelines.xhtml

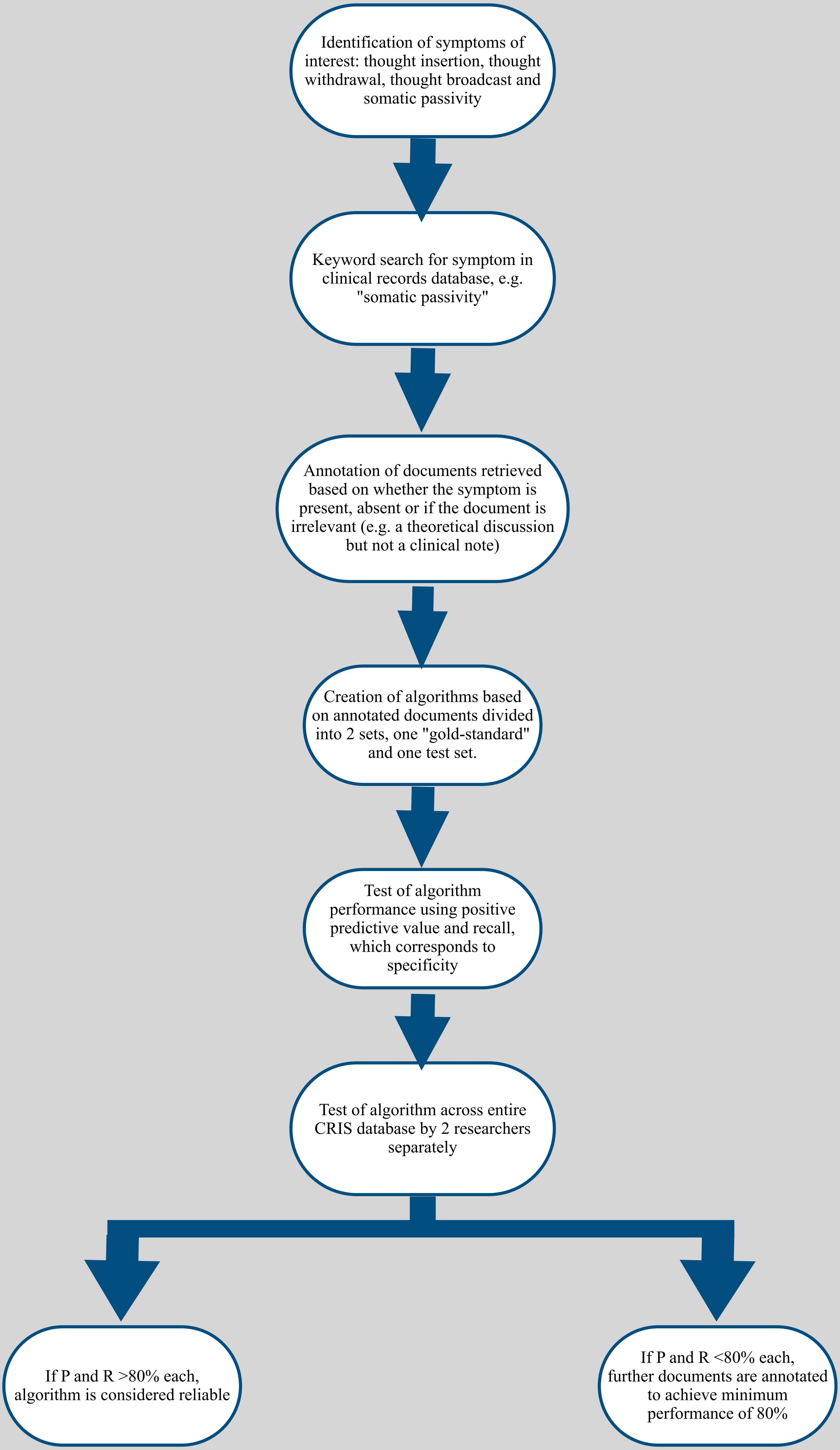
Identification of symptoms of interest: thought insertion, thought withdrawal, thought broadcast and somatic passivity
Keyword search for symptom in clinical records database, e.g. "somatic passivity"
Annotation of documents retrieved based on whether the symptom is present, absent or if the document is irrelevant (e.g. a theoretical discussion but not a clinical note)
Creation of algorithms based on annotated documents divided into 2 sets, one "gold-standard" and one test set.
Test of algorithm performance using positive predictive value and recall, which corresponds to specificity
Test of algorithm across entire CRIS database by 2 researchers separately
If P and R >80% each, algorithm is considered reliable
If P and R <80% each, further documents are annotated to achieve minimum performance of 80%

Supplementary Material

Supplementary Table 1: Natural language processing algorithm development and performance. A minimum P and R of 80% is required for an algorithm to be considered reliable. In the context of passivity, the lower IRR led to the inclusion of extra annotations that enabled us to reach this threshold.

Symptom	Keyword	Number of annotations	IRR	P and R	Extra steps required	Manual testing	Keyword search P and R
Thought insertion	"thought insert*"	592 (96 double-annoted)	97 %	P=81%; R=94%	N/A	P=84%	P=81%; R=96%
Thought broadcasting	"thought broadcast*"	398 (95 double-annoted)	94 %	P=81%; R=94%	N/A	P=84%	P=81%; R=96%
Thought withdrawal	"thought withdraw*"	500 (76 double-annotated)	95 %	P=74%; R=91%	159 extra annotations	P=84%	P= 90%; R=88%
Somatic passivity	"passivity"	500 (116 double-annotated)	71 %	P=72%; R=92%	597 extra annotations, form excluded	P=82%	P=89%; R=100%

Supplementary Table 2: Unadjusted odds ratios for the co-occurrence of psychotic symptoms across the patient sample

Symptom	Broadcast	Insertion	Withdrawal	Passivity	Paranoia	Auditory Hallucinations	Persecutory Delusions
Broadcast	1						
Insertion	11.67	1					
Withdrawal	6.05	12.10	1				
Passivity	5.13	6.66	3.86	1			
Paranoia	5.29	3.97	3.32	4.67	1		
Auditory Hallucinations	5.33	5.06	3.45	4.99	3.50	1	
Persecutory Delusions	3.13	3.30	2.41	3.40	5.30	2.80	1

Supplementary Table 3: Prevalence of negative outcomes in patients with somatic passivity or thought interference at different ages

Age (years)	Occurrence on negative outcomes in patients with somatic passivity (%) (95% CI)	Occurrence on negative outcomes in patients with thought interference (%) (95% CI)
20	59.6 (58.0-61.1)	59.1 (57.6-60.7)
30	53.7 (52.5-54.9)	53.4 (52.2-54.6)
40	47.7 (46.6-48.7)	47.7 (46.6-48.7)
50	41.7 (40.5-43.0)	41.9 (40.7-43.2)
60	36.1 (34.5-37.6)	36.4 (34.8-38.0)
70	30.7 (28.8-32.6)	31.2 (29.3-33.2)
80	25.9 (23.7-28.0)	26.5 (24.3-28.7)
90	21.5 (19.2-23.8)	22.2 (19.8-24.6)

Supplementary Table 4: Linear regression describing the influence of somatic passivity, thought interference and its subcomponents on the number of antipsychotics prescribed

Symptom	Standardized Beta Coefficients (95% CI)	t	Significance
Somatic Passivity	0.35 (0.24-0.45)	6.43	0.00
Thought Interference	0.40 (-0.14-0.22)	0.43	0.67
Thought Insertion	0.14 (0-0.01-0.30)	1.81	0.07
Thought Withdrawal	-0.00 (-0.17-0.16)	-0.05	0.96
Thought Broadcasting	-0.02 (-0.18-0.13)	-0.29	0.77

Adjusted for age at diagnosis, gender, ethnicity, diagnosis, IMD quartile and presence of paranoia, auditory hallucinations, persecutory delusion.

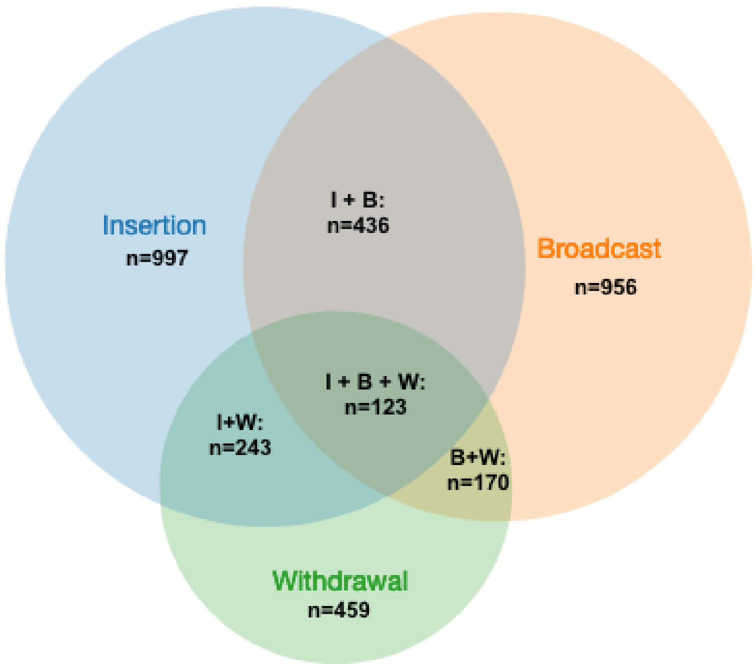

Supplementary Figure 1: Venn diagram describing the overlap between thought broadcasting, thought insertion and thought withdrawal. The overlap observed before further analyses suggested that there may exist interactions linking these factors and directed our analyses towards the development of a model to understand these better.

For peer review only - http://bmjopen.bmj.com/site/about/guidelines.xhtml

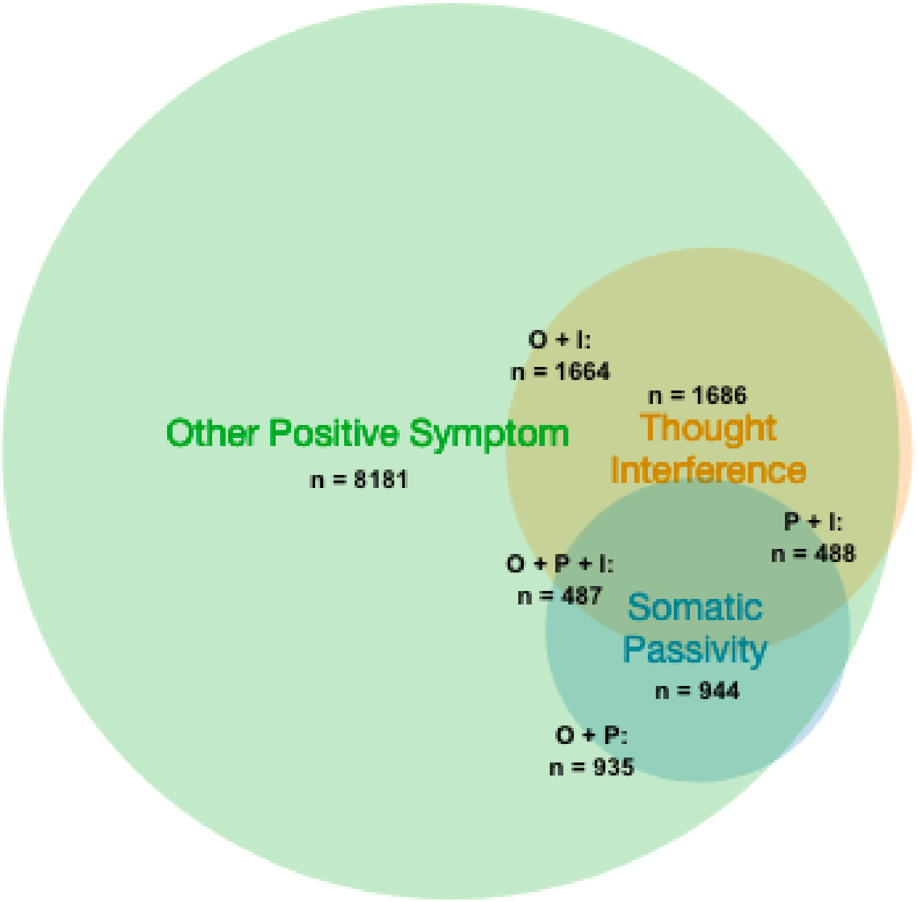

Supplementary figure 2: Venn diagram describing the overlap between somatic passivity, thought interference and any other positive symptoms (auditory hallucinations, persecutory delusions, paranoia) . I: Thought Interference; P: Passivity; O: Other Positive Symptom. When carrying out analyses, other positive symptoms were not combined but each included as individual covariates. The overlap observed here suggested that there may exist interactions linking these factors and directed our analyses towards the development of a model to understand these better. this diagram also reflects the prevalence of other positive symptoms and the need for investigations of the possible correlation between these and outcomes in the future.

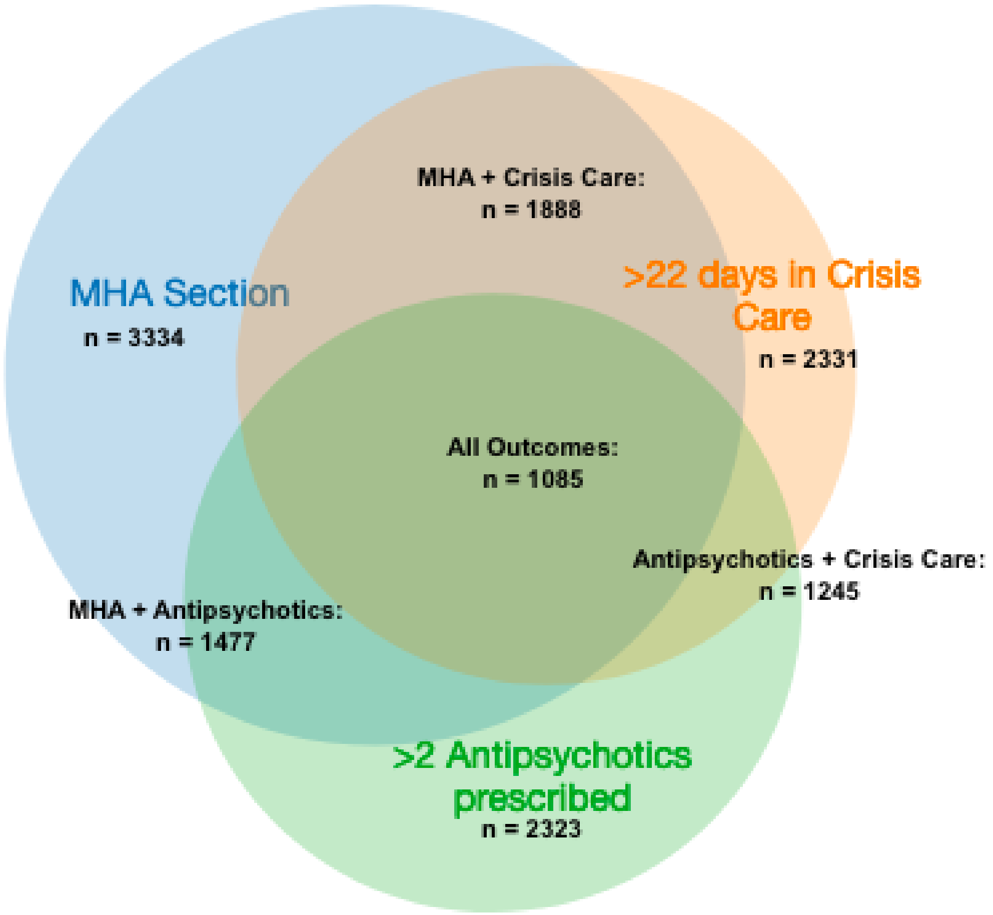

Supplementary Figure 3: Venn diagram describing the overlap between Outcomes investigated. MHA: presence of a Mental Health act section. This diagram highlights the degree of overlap between negative outcomes, suggesting a possible correlation in their occurrence.

Supplementary Material

Supplementary Table 1: Natural language processing algorithm development and performance. A minimum P and R of 80% is required for an algorithm to be considered reliable. In the context of passivity, the lower IRR led to the inclusion of extra annotations that enabled us to reach this threshold.

Symptom	**Keyword**	**Number of annotations**	**IRR**	**P and R**	**Extra steps required**	**Manual testing**	**Keyword search P and R**
Thought insertion	"thought insert*"	592 (96 double-annoted)	97 %	P=81%; R=94%	N/A	P=84%	P=81%; R=96%
Thought broadcasting	"thought broadcast*"	398 (95 double-annoted)	94 %	P=81%; R=94%	N/A	P=84%	P=81%; R=96%
Thought withdrawal	"thought withdraw*"	500 (76 double-annotated)	95 %	P=74%; R=91%	159 extra annotations	P=84%	P= 90%; R=88%
Somatic passivity	"passivity"	500 (116 double-annotated)	71 %	P=72%; R=92%	597 extra annotations, form excluded	P=82%	P=89%; R=100%

For peer review only - http://bmjopen.bmj.com/site/about/guidelines.xhtml

Supplementary Table 2: Unadjusted odds ratios for the co-occurrence of psychotic symptoms across the patient sample

Symptom	Broadcast	Insertion	Withdrawal	Passivity	Paranoia	Auditory Hallucinations	Persecutory Delusions
Broadcast	1						
Insertion	11.67	1					
Withdrawal	6.05	12.10	1				
Passivity	5.13	6.66	3.86	1			
Paranoia	5.29	3.97	3.32	4.67	1		
Auditory Hallucinations	5.33	5.06	3.45	4.99	3.50	1	
Persecutory Delusions	3.13	3.30	2.41	3.40	5.30	2.80	1

Supplementary Table 3: Prevalence of negative outcomes in patients with somatic passivity or thought interference at different ages

Age (years)	Occurrence on negative outcomes in patients with somatic passivity (%) (95% CI)	Occurrence on negative outcomes in patients with thought interference (%) (95% CI)
20	59.6 (58.0-61.1)	59.1 (57.6-60.7)
30	53.7 (52.5-54.9)	53.4 (52.2-54.6)
40	47.7 (46.6-48.7)	47.7 (46.6-48.7)
50	41.7 (40.5-43.0)	41.9 (40.7-43.2)
60	36.1 (34.5-37.6)	36.4 (34.8-38.0)
70	30.7 (28.8-32.6)	31.2 (29.3-33.2)
80	25.9 (23.7-28.0)	26.5 (24.3-28.7)
90	21.5 (19.2-23.8)	22.2 (19.8-24.6)

For peer review only - http://bmjopen.bmj.com/site/about/guidelines.xhtml

Supplementary Table 4: Linear regression describing the influence of somatic passivity, thought interference and its subcomponents on the number of antipsychotics prescribed

Symptom	Standardized Beta Coefficients (95% CI)	t	Significance
Somatic Passivity	0.35 (0.24-0.45)	6.43	0.00
Thought Interference	0.40 (-0.14-0.22)	0.43	0.67
Thought Insertion	0.14 (0-0.01-0.30)	1.81	0.07
Thought Withdrawal	-0.00 (-0.17-0.16)	-0.05	0.96
Thought Broadcasting	-0.02 (-0.18-0.13)	-0.29	0.77

Adjusted for age at diagnosis, gender, ethnicity, diagnosis, IMD quartile and presence of paranoia, auditory hallucinations, persecutory delusion.

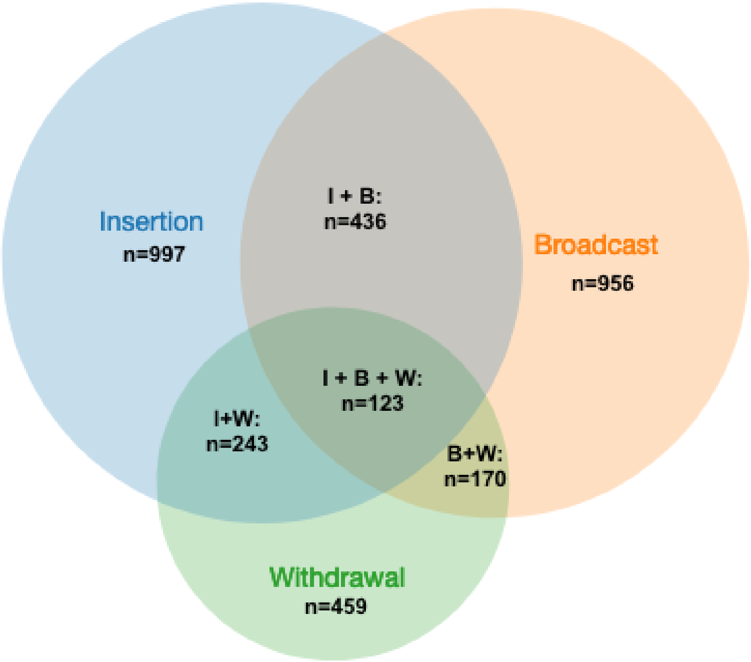

Supplementary Figure 1: Venn diagram describing the overlap between thought broadcasting, thought insertion and thought withdrawal. The overlap observed before further analyses suggested that there may exist interactions linking these factors and directed our analyses towards the development of a model to understand these better.

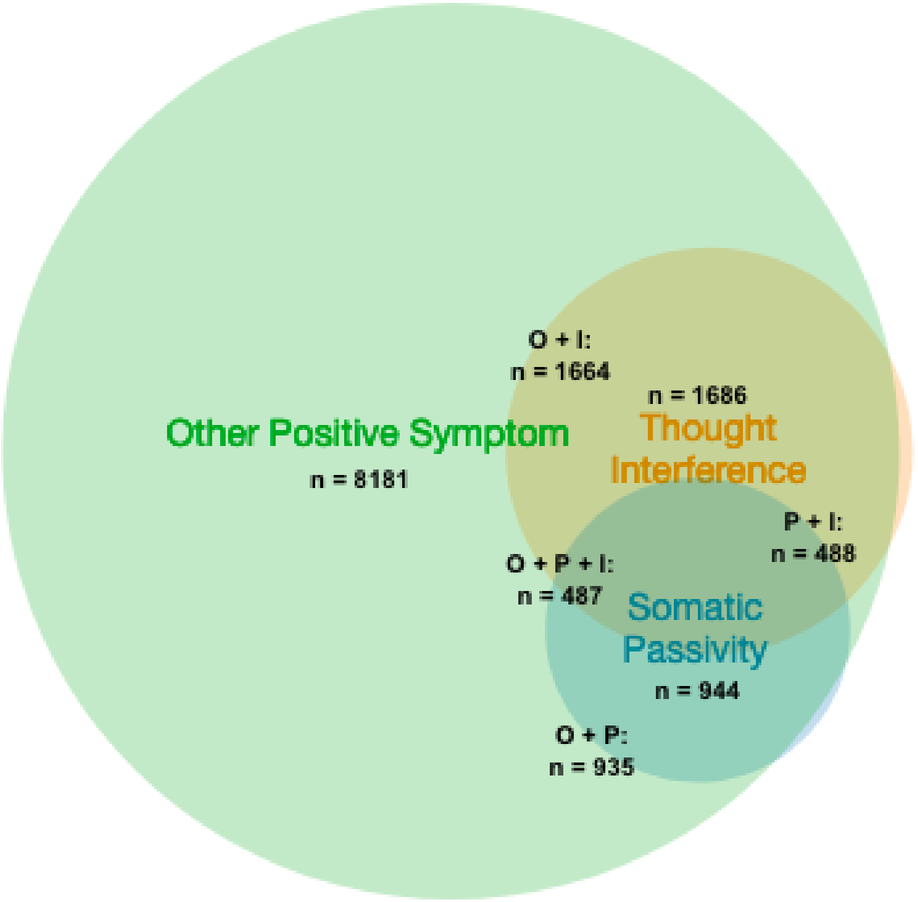

Supplementary figure 2: Venn diagram describing the overlap between somatic passivity, thought interference and any other positive symptoms (auditory hallucinations, persecutory delusions, paranoia) . I: Thought Interference; P: Passivity; O: Other Positive Symptom. When carrying out analyses, other positive symptoms were not combined but each included as individual covariates. The overlap observed here suggested that there may exist interactions linking these factors and directed our analyses towards the development of a model to understand these better. this diagram also reflects the prevalence of other positive symptoms and the need for investigations of the possible correlation between these and outcomes in the future.

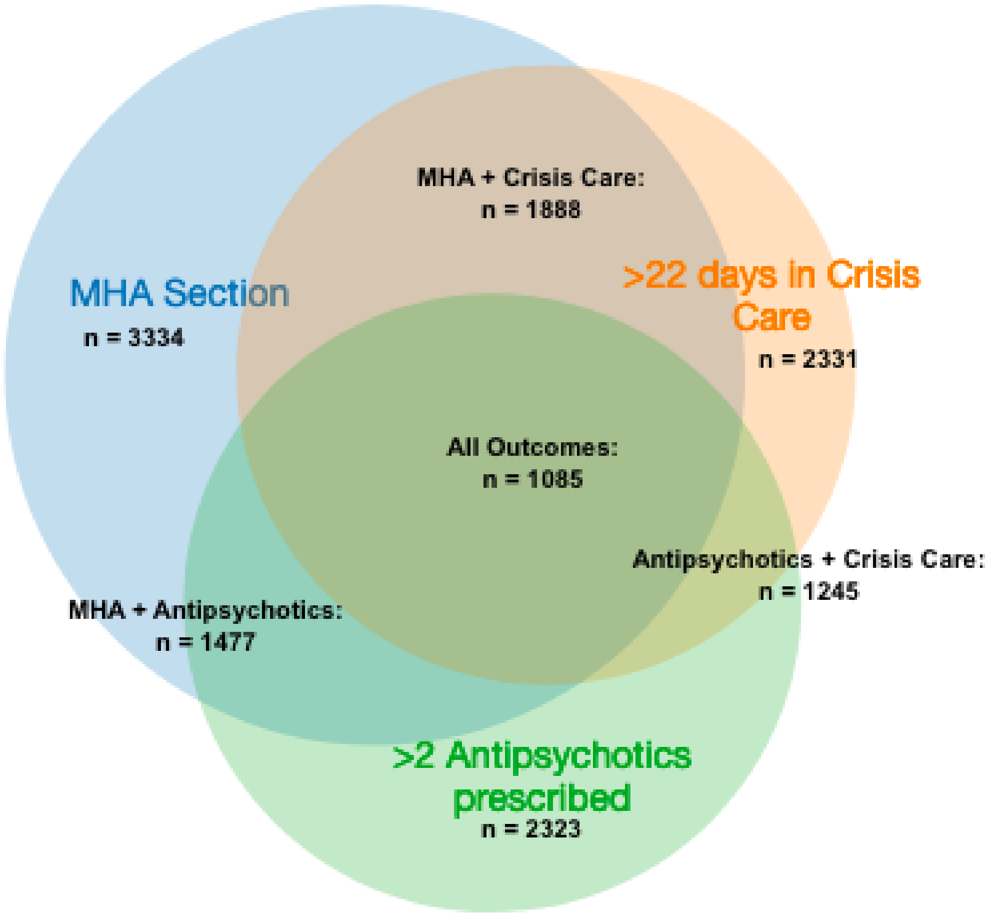

Supplementary Figure 3: Venn diagram describing the overlap between Outcomes investigated. MHA: presence of a Mental Health act section. This diagram highlights the degree of overlap between negative outcomes, suggesting a possible correlation in their occurrence.

For peer review only - http://bmjopen.bmj.com/site/about/guidelines.xhtml

The RECORD statement – checklist of items, extended from the STROBE statement, that should be reported in observational studies using routinely collected health data.

	Item No.	STROBE items	Location in manuscript where items are reported	RECORD items	Location in manuscript where items are reported
**Title and abstract**					
	1	(a) Indicate the study's design with a commonly used term in the title or the abstract (b) Provide in the abstract an informative and balanced summary of what was done and what was found	Title page and abstract (p1&2)	RECORD 1.1: The type of data used should be specified in the title or abstract. When possible, the name of the databases used should be included.<br><br>RECORD 1.2: If applicable, the geographic region and timeframe within which the study took place should be reported in the title or abstract.<br><br>RECORD 1.3: If linkage between databases was conducted for the study, this should be clearly stated in the title or abstract.	Title page (p1) and abstract (p2)<br><br>Abstract (p2)<br><br>N/A
**Introduction**					
Background rationale	2	Explain the scientific background and rationale for the investigation being reported	Introduction (p3-5)		
Objectives	3	State specific objectives, including any prespecified hypotheses	Abstract (p2) and Introduction (p4&5, final paragraph)		
**Methods**					
Study Design	4	Present key elements of study design early in the paper	Methods (p6-9)		
Setting	5	Describe the setting, locations, and relevant dates, including periods of recruitment, exposure, follow-up, and data collection	Methods, (p6-9)		

For peer review only

Participants	6	*(a) Cohort study* - Give the eligibility criteria, and the sources and methods of selection of participants. Describe methods of follow-up<br>*Case-control study* - Give the eligibility criteria, and the sources and methods of case ascertainment and control selection. Give the rationale for the choice of cases and controls<br>*Cross-sectional study* - Give the eligibility criteria, and the sources and methods of selection of participants<br><br>*(b) Cohort study* - For matched studies, give matching criteria and number of exposed and unexposed<br>*Case-control study* - For matched studies, give matching criteria and the number of controls per case		RECORD 6.1: The methods of study population selection (such as codes or algorithms used to identify subjects) should be listed in detail. If this is not possible, an explanation should be provided.<br><br>RECORD 6.2: Any validation studies of the codes or algorithms used to select the population should be referenced. If validation was conducted for this study and not published elsewhere, detailed methods and results should be provided.<br><br>RECORD 6.3: If the study involved linkage of databases, consider use of a flow diagram or other graphical display to demonstrate the data linkage process, including the number of individuals with linked data at each stage.	Methods, subsections "context" and "patient sample" (p6)<br><br>Methods, subsection "exposure variables and software development" (p7&8)<br><br>N/A
Variables	7	Clearly define all outcomes, exposures, predictors, potential confounders, and effect modifiers. Give diagnostic criteria, if applicable.	Methods (p6-9)	RECORD 7.1: A complete list of codes and algorithms used to classify exposures, outcomes, confounders, and effect modifiers should be provided. If these cannot be reported, an explanation should be provided.	Methods (p6-9)
Data sources/ measurement	8	For each variable of interest, give sources of data and details of methods of assessment (measurement).<br>Describe comparability of assessment methods if there is more than one group	Methods (p6-9)		

For peer review only

Bias	9	Describe any efforts to address potential sources of bias	Methods, subsections "covariates" and "statistical analysis" (p8&9)		
Study size	10	Explain how the study size was arrived at	Methods, subsection "patient sample" (p6)		
Quantitative variables	11	Explain how quantitative variables were handled in the analyses. If applicable, describe which groupings were chosen, and why	Methods (p6-9)		
Statistical methods	12	(a) Describe all statistical methods, including those used to control for confounding<br>(b) Describe any methods used to examine subgroups and interactions<br>(c) Explain how missing data were addressed<br>(d) *Cohort study* - If applicable, explain how loss to follow-up was addressed<br>*Case-control study* - If applicable, explain how matching of cases and controls was addressed<br>*Cross-sectional study* - If applicable, describe analytical methods taking account of sampling strategy<br>(e) Describe any sensitivity analyses	Methods (p6-9)		
Data access and cleaning methods		..		RECORD 12.1: Authors should describe the extent to which the investigators had access to the database	Methods (p6-9)

For peer review only

				population used to create the study population.<br><br>RECORD 12.2: Authors should provide information on the data cleaning methods used in the study.	Methods (p6-9)
Linkage		..		RECORD 12.3: State whether the study included person-level, institutional-level, or other data linkage across two or more databases. The methods of linkage and methods of linkage quality evaluation should be provided.	N/A
**Results**					
Participants	13	(a) Report the numbers of individuals at each stage of the study (*e.g.*, numbers potentially eligible, examined for eligibility, confirmed eligible, included in the study, completing follow-up, and analysed)<br>(b) Give reasons for non-participation at each stage.<br>(c) Consider use of a flow diagram	Methods, subsection "patient sample" (p6)	RECORD 13.1: Describe in detail the selection of the persons included in the study (*i.e.,* study population selection) including filtering based on data quality, data availability and linkage. The selection of included persons can be described in the text and/or by means of the study flow diagram.	Methods, subsection "patient sample" (p6)
Descriptive data	14	(a) Give characteristics of study participants (*e.g.*, demographic, clinical, social) and information on exposures and potential confounders<br>(b) Indicate the number of participants with missing data for each variable of interest<br>(c) *Cohort study* - summarise follow-up time (*e.g.*, average and total amount)	Results (p10&11, table 1)		

For peer review only

Outcome data	15	*Cohort study* - Report numbers of outcome events or summary measures over time<br>*Case-control study* - Report numbers in each exposure category, or summary measures of exposure<br>*Cross-sectional study* - Report numbers of outcome events or summary measures	Results (p12&13, table 2)		
Main results	16	(a) Give unadjusted estimates and, if applicable, confounder-adjusted estimates and their precision (e.g., 95% confidence interval). Make clear which confounders were adjusted for and why they were included<br>(b) Report category boundaries when continuous variables were categorized<br>(c) If relevant, consider translating estimates of relative risk into absolute risk for a meaningful time period	Results (p14-19, tables 3-5)		
Other analyses	17	Report other analyses done—e.g., analyses of subgroups and interactions, and sensitivity analyses	Methods and Results (p6-19 - particularly subsection statistical analysis, p9; other tests also mentioned in Results where relevant)		
**Discussion**					
Key results	18	Summarise key results with reference to study objectives	Discussion (p20&21)		
Limitations	19	Discuss limitations of the study, taking into account sources of potential bias or imprecision.	Strengths and Limitations (p23-24)	RECORD 19.1: Discuss the implications of using data that were not created or collected to answer the specific research question(s). Include	Strengths and Limitations (p23-24)

For peer review only

		Discuss both direction and magnitude of any potential bias		discussion of misclassification bias, unmeasured confounding, missing data, and changing eligibility over time, as they pertain to the study being reported.	
Interpretation	20	Give a cautious overall interpretation of results considering objectives, limitations, multiplicity of analyses, results from similar studies, and other relevant evidence	Discussion (p20-23)		
Generalisability	21	Discuss the generalisability (external validity) of the study results	Strengths and Limitations (p23-24)		
**Other Information**					
Funding	22	Give the source of funding and the role of the funders for the present study and, if applicable, for the original study on which the present article is based	Funding and Conflict of Interest (p26)		
Accessibility of protocol, raw data, and programming code		..		RECORD 22.1: Authors should provide information on how to access any supplemental information such as the study protocol, raw data, or programming code.	Methods subsection "exposure variables and software Development" (p7): CRIS open-access online catalogue reference

*Reference: Benchimol EI, Smeeth L, Guttmann A, Harron K, Moher D, Petersen I, Sørensen HT, von Elm E, Langan SM, the RECORD Working Committee. The REporting of studies Conducted using Observational Routinely-collected health Data (RECORD) Statement. *PLoS Medicine* 2015; in press.

*Checklist is protected under Creative Commons Attribution (CC BY) license.

BMJ Open

Investigating the relationship between thought interference, somatic passivity and outcomes in patients with psychosis: a natural-language processing approach using a clinical records search platform in south London

Journal:	*BMJ Open*
Manuscript ID	bmjopen-2021-057433.R3
Article Type:	Original research
Date Submitted by the Author:	18-Jul-2022

Complete List of Authors:	Magrangeas, Thibault; King's College London Institute of Psychiatry Psychology and Neuroscience<br>Kolliakou, Anna; King's College London Institute of Psychiatry Psychology and Neuroscience, Institute of Psychiatry<br>Sanyal, Jyoti; South London and Maudsley NHS Foundation Trust<br>Patel, Rashmi; King's College London Institute of Psychiatry Psychology and Neuroscience; South London and Maudsley NHS Foundation Trust<br>Stewart, Robert; King's College London Institute of Psychiatry Psychology and Neuroscience; South London and Maudsley NHS Foundation Trust

**Primary Subject Heading**:	Mental health
Secondary Subject Heading:	Health informatics
Keywords:	Schizophrenia & psychotic disorders < PSYCHIATRY, Adult psychiatry < PSYCHIATRY, Health informatics < BIOTECHNOLOGY & BIOINFORMATICS

SCHOLARONE™
Manuscripts

I, the Submitting Author has the right to grant and does grant on behalf of all authors of the Work (as defined in the below author licence), an exclusive licence and/or a non-exclusive licence for contributions from authors who are: i) UK Crown employees; ii) where BMJ has agreed a CC-BY licence shall apply, and/or iii) in accordance with the terms applicable for US Federal Government officers or employees acting as part of their official duties; on a worldwide, perpetual, irrevocable, royalty-free basis to BMJ Publishing Group Ltd ("BMJ") its licensees and where the relevant Journal is co-owned by BMJ to the co-owners of the Journal, to publish the Work in this journal and any other BMJ products and to exploit all rights, as set out in our licence.

The Submitting Author accepts and understands that any supply made under these terms is made by BMJ to the Submitting Author unless you are acting as an employee on behalf of your employer or a postgraduate student of an affiliated institution which is paying any applicable article publishing charge ("APC") for Open Access articles. Where the Submitting Author wishes to make the Work available on an Open Access basis (and intends to pay the relevant APC), the terms of reuse of such Open Access shall be governed by a Creative Commons licence – details of these licences and which Creative Commons licence will apply to this Work are set out in our licence referred to above.

Other than as permitted in any relevant BMJ Author's Self Archiving Policies, I confirm this Work has not been accepted for publication elsewhere, is not being considered for publication elsewhere and does not duplicate material already published. I confirm all authors consent to publication of this Work and authorise the granting of this licence.

For peer review only

Investigating the relationship between thought interference, somatic passivity and outcomes in patients with psychosis: a natural-language processing approach using a clinical records search platform in south London

Thibault Magrangeas[1], Anna Kolliakou[1], Jyoti Sanyal[2], Rashmi Patel[1&2], Robert Stewart[1&2]

Affiliations:

1: King's College London (Institute of Psychiatry, Psychology and Neuroscience), London, UK

2: South London and Maudsley NHS Foundation Trust, London, UK

Correspondence to:

Thibault Magrangeas

Department of Psychological Medicine

Main Building E3.08

Institute of Psychiatry, Psychology and Neuroscience

King's College London

De Crespigny Park

London SE5 8AF

United Kingdom

E-mail: thibault.magrangeas@kcl.ac.uk

Abstract

Objectives: We aimed to apply natural language-processing (NLP) algorithms in routine healthcare records to identify reported somatic passivity (external control of sensations, actions and impulses) and thought interference symptoms (thought broadcasting, insertion, withdrawal), first-Rank symptoms traditionally central to diagnosing schizophrenia, and determine associations with prognosis by analysing routine outcomes.

Design: Four algorithms were developed on de-identified mental healthcare data and applied to ascertain recorded symptoms over the three months following first presentation to a mental healthcare provider in a cohort of patients with a primary schizophreniform disorder (ICD-10 F20-F29) diagnosis.

Setting and participants: From the electronic health records of a large secondary mental healthcare provider in south London, 9,323 patients were ascertained from 2007 to the data extraction date (25/02/2020).

Outcomes: The primary binary dependent variable for logistic regression analyses was any negative outcome (Mental Health Act section, >2 antipsychotics prescribed, >22 days spent in crisis care) over the subsequent 2 years.

Results: Final adjusted models indicated significant associations of this composite outcome with baseline somatic passivity (prevalence 4.9%; adjusted odds ratio 1.61, 95% CI 1.37-1.88), thought insertion (10.7%; 1.24, 1.15-1.55) and thought withdrawal (4.9%; 1.36, 1.10-1.69), but not independently with thought broadcast (10.3%; 1.05, 0.91-1.22).

Conclusions: Symptoms traditionally central to the diagnosis of schizophrenia, but under-represented in current diagnostic frameworks, were thus identified as important predictors of short- to medium-term prognosis in schizophreniform disorders.

For peer review only - http://bmjopen.bmj.com/site/about/guidelines.xhtml

Keywords: Schizophrenia; Delusions; Prognosis; Psychosis; Patient Outcomes; Natural-Language Processing (NLP)

Strengths and limitations of this study

- This study was carried out on a very large patient sample and identified symptoms from routine care instead of specifically recruited and therefore less representative cohorts, and our applications achieved high-performance metrics.
- The patient sample was drawn from an inner urban area in South London, so results may not be entirely generalisable to the rest of the population.
- The recording of exposure variables relied on symptoms being elicited and noted by clinicians in routine practice and therefore cannot be assumed to be equivalent to those ascertained in a more research-focused interview, just as covariates such as diagnosis also were derived from routine clinical records and should not be assumed to equate research diagnostic criteria; however, we feel that inaccuracies in exposure ascertainment were more likely to obscure findings of interest through non-differential measurement error rather than give rise to spurious associations.
- Although multiple confounding factors were accounted for, our model-fit measures demonstrate model reliability but highlight residual unexplained variance possibly due to the influence of other psychotic symptoms as well as additional external factors that may affect outcomes and should be further investigated; our model is useful in correlating patient outcomes with symptoms at first presentation but it is not possible to infer stability of the clinical construct over time as the patient is only assessed once.
- Outcomes analysed were restricted to those most readily available from the source data and most likely to be clinically informative; however, they cannot be viewed as exhaustive and there may be elements of prognosis that were not captured in this study, including interactions between outcomes and previous adverse events that may influence symptom occurrence and recording.

For peer review only - http://bmjopen.bmj.com/site/about/guidelines.xhtml

INTRODUCTION

Schizophrenia was originally defined by Schneider around a core group of first-rank symptoms (FRS) which include auditory hallucinations (commonly characterised as but not limited to hearing one or multiple voices talking to or about the patient), delusions of perception (such as the sensation of being externally controlled) or thought interference (the sensation of having thoughts inserted, withdrawn or externally broadcasted against the patient's wishes) [1]. These symptoms were identified as central to diagnosing schizophrenia as they were observed in the majority of cases. Still today, recent studies have consistently found FRS in the form of thought interference and somatic passivity to be core symptoms of psychotic disorders in general and schizophrenia specifically[2-4]. A comparative study of thirteen different diagnostic systems identified a triad of positive symptoms strongly correlated with a schizophrenia diagnosis across all scales, the presence of two of which was sufficient to diagnose 78% of patients; these were auditory hallucinations, disturbances of affect and "passivity feelings" (defined as "any delusion of being influenced or interfered with by imaginary forces from outside, whether somatically or mentally" and therefore assumed to include thought interference)[5]; these symptoms were all already described in Bleuler's 1911 definition of schizophrenia[6], demonstrating historical consistency. Somatic passivity and thought interference have also been reported to be positively correlated and predict the occurrence of schizophrenia or schizoaffective disorder[7-8].

Despite this, ongoing research into the complex mechanisms underpinning psychosis manifestations has highlighted the wider range of symptoms that can occur and encouraged a new approach focused beyond these core FRS. For example, different symptoms have been observed to occur in clusters, such as somatic passivity and persecutory delusions[9] or auditory verbal hallucinations[10]. Environmental factors also seem to influence symptom occurrence,

leading to debate around the cross-cultural relevance of FRS[11]. In this context, recent changes to the DSM-5 including the removal of FRS from diagnostic criteria[12-13] have been welcomed as a valuable push towards a better understanding of individual symptoms; however, limitations remain as this new focus has been criticised for not fully addressing the relationship between symptoms; conflicting with most existing guidelines for early symptom detection in schizophrenia that still heavily rely on FRS; and diverting attention away from core psychopathological processes at the disorder's root[14].

Importantly, schizophrenia symptoms can directly influence both behaviour and consequently patient outcomes; for example, indirectly leading to violence through the occurrence of delusions[15]. FRS absence has been found to correlate with better outcomes, leading to a proposed redefinition of schizophrenia as a continuum, exhibiting a positive linear relationship between psychotic symptoms and outcomes[16-18]. Furthermore, the prevalences of individual symptoms appear to vary between early- and late-onset cases, reflecting complex interactions between symptoms and disease development[19-21]. This evidence suggests that patient outcomes are better in schizophrenia in the absence of FRS[17] but offers no insight into prognosis once present or into relationships between symptoms. While some authors have identified differing effects on outcomes, with passivity predicting a worsened prognosis in contrast to interference[17], others have highlighted a possible link, as both phenomenologically reflect a belief in external control, suggesting they may have a synergistic effect on outcomes[8,22-23] or even constitute components of the same delusion[10&24] or syndrome[25]. Overall, these studies highlight the significance of positive symptoms individually and as a group, suggesting possible correlations and reflecting the overlap in their occurrence, although the degree of overlap is rarely quantified.

For peer review only - http://bmjopen.bmj.com/site/about/guidelines.xhtml

The evidence highlighted here reflects the importance of clarifying the role of FRS in prognosis to understand which aspects of schizophrenia should be at the core of its diagnosis. Modern technologies have allowed the development of new tools allowing the analysis of large datasets using automated software. This is the goal of the South London and Maudsley (SLaM) NHS Foundation trust Clinical Record Interactive Search (CRIS) platform was developed in 2008 to enable research on a de-identified copy of the Trust's electronic health records (used across all services since 2006) within a robust governance and data security framework[24]. All the clinical notes of patients are recorded in a de-identified format on this database. Natural Language Processing (NLP) algorithms are then used to study these documents; these are algorithms that can be trained to recognise entities of interest and their context in free text, for example the mention of "thought interference" in a way that indicates that a patient is experiencing this symptom.

This study aimed to develop and evaluate four NLP algorithms to detect the recording of thought interference subcomponents (broadcasting, insertion, withdrawal) and somatic passivity across this database. Other algorithms had already been developed to capture the recorded occurrence of other important positive symptoms of psychosis (paranoia, auditory hallucinations, persecutory delusions) and were used to define potential covariates. We focused on individuals diagnosed with a schizophreniform disorder and analysed patient outcomes in this population. Our focus on thought interference and somatic passivity as particular symptoms aimed to clarify the interactions described previously which suggested a particularly strong relationship between these symptoms; in addition, they were chosen as potentially tractable to extraction at scale from routine mental healthcare data via NLP. Being described in relatively consistent language in clinical records, they made a pragmatic choice for further investigation in contrast to other symptoms such as running commentary hallucinations which

For peer review only - http://bmjopen.bmj.com/site/about/guidelines.xhtml

are typically described with greater linguistic heterogeneity (i.e., based on the individual experience of patients rather than a set vocabulary) and therefore harder to study with this approach.

For peer review only - http://bmjopen.bmj.com/site/about/guidelines.xhtml

METHODS

Context

We used data extracted from the electronic mental health clinical notes records of patients at the South London and Maudsley NHS Foundation Trust (SLaM), one of the largest mental health service providers in Europe, serving a geographic catchment of four South London boroughs (Southwark, Lambeth, Lewisham, Croydon) with around 1.3 million residents. The Trust provides care to 37,500 active patients, with 12,000 to 14,000 clinical events per week covering a diverse range of interventions in the community and in the inpatient setting[26&27]. SLaM's Clinical Record Interactive Search (CRIS) platform was developed in 2008 to enable research on a de-identified copy of the Trust's electronic health records (used across all services since 2006) within a robust governance and data security framework[24]. CRIS has been described in detail previously[28&29] and has supported over 250 research publications to date. Ethical approval as a data resource for secondary analysis in in place (Oxford Research Ethics Committee C, reference 18/SC/0372).

Patient sample

All patients aged 16-95 with a primary F20-F29 disorder diagnosis according to ICD-10 criteria recorded from 2007 to extraction date (25/02/2020) were included. This age-range was chosen to cover patients transitioning to adult mental health care, commonly occurring from age 16; patients with no recorded date of birth appear on the system as aged 100+ and represented all the individuals above age 95; they were therefore excluded to avoid skew when correcting for age. According to these criteria, 9,536 patients were identified. Thought interference and somatic passivity occurrence in the three months following first presentation at SLaM (defined as first face-to-face contact) was sought. If multiple diagnoses were then recorded for the same patient, first diagnosis was used. This cut-off was chosen to allow time for clinicians to assess

For peer review only - http://bmjopen.bmj.com/site/about/guidelines.xhtml

all patients and record their symptoms accurately and focus on patients presenting with these symptoms as first presentation rather than later development. Participants who died within this period were excluded. Of the initial 9,536 patients, 9,323 (97.8%) were included, 57.9% of whom were male. Mean age at inclusion was 39.9 years (SD=17.3).

Exposure variables and software development

CRIS data have been enhanced over the last 10 years by a range of NLP algorithms allowing a hitherto unavailable level of detail to be generated at scale from routine healthcare data, taking advantage of the level of detail recorded in extensive text fields. The algorithms, over 90 to date, are described individually in an open-access online catalogue[30], including detailed information on definitions and performance. A particular objective has been to provide, at scale, broader phenotypic information on people presenting with mental disorders than that supplied by clinician-assigned diagnostic codes, through the extraction of recorded symptoms with 60 algorithms to date developed to ascertain individual symptoms across psychotic and affective disorders[31]. Contributing to this wider initiative, and with the specific rationale in mind for this study, we developed four new algorithms to identify instances of recorded somatic passivity and thought insertion, withdrawal and broadcasting in CRIS using the TextHunter platform[32], a tool used to present short segments of text to an annotator in order to provide rapid large samples for the training of machine learning algorithms, as well as a platform for independent evaluation of algorithm output (figure 1).

The data analysed consisted of de-identified text from all inpatient and outpatient clinical notes and correspondence. The algorithms used were applied to text surrounding relevant keywords corresponding to each variable of interest; these keywords were defined by the authors to ensure detecting as wide a range of formulations as possible. For example, to detect free-text

For peer review only - http://bmjopen.bmj.com/site/about/guidelines.xhtml

mentions of passivity delusions, the keyword "passiv*" was used, with the asterisk allowing for different formulations such as "passive" or "passivity". The documents identified were then manually annotated via the TextHunter platform to train the algorithm to automatically determine if this was relevant; for example, text stating that "the patient described passive external control of actions" would be marked as positive, text stating "the patient described a passive lifestyle" would be marked as irrelevant, and text stating “no passivity” would be marked as a negation statement. Once the algorithm had been optimized, independent evaluation took place on novel text to ascertain precision (proportion of algorithm-identified positive instances found to be correct on evaluation) and recall (proportion of positive entities identified as such by the algorithm) statistics. Testing the performance of the algorithm over the entire CRIS database was performed by checking 100 random annotated documents produced by the algorithm and 100 random unannotated documents using keyword searches. this constituted a gold-standard set from which a second researcher double-annotated a proportion of the retrieved documents to determine inter-rater reliability (IRR) using Cohen's kappa. The process was iterative and sought to achieve P and R values over 80%. High performance metrics were attained (supplementary table 1). For primary analyses, positive indications of the three symptoms representing thought interference (thought insertion, thought withdrawal, thought broadcast) were combined under that a single entity with secondary analyses considering them separately; all symptoms were defined as binary variables according to their recording or not in the first three months after first presentation.

Outcomes

Occurrence of negative outcomes was recorded over the two years following first presentation at SLaM. Three outcomes were evaluated: i) highest-quartile time spent as an inpatient or receiving support from a home treatment team in the community since first presentation

For peer review only - http://bmjopen.bmj.com/site/about/guidelines.xhtml

(defined as 22 days or more); ii) receiving more than two antipsychotics; iii) involuntary psychiatric hospitalisation defined as detention for assessment or treatment under Sections 2 or 3 of the UK Mental Health Act (MHA).

Covariates

Included covariates were: gender, ethnic group, age at first SLaM presentation, diagnosis, Index of Multiple Deprivation (IMD) score and presence of recorded paranoia, persecutory delusions or auditory hallucinations. Age was categorised by decades for descriptive analyses but applied as a continuous variable otherwise. Diagnoses were recorded as a binary variable, distinguishing schizophrenia (ICD-10 F20x) from other schizophreniform disorders (F21-F29)[33]. The IMD score is a measure of neighbourhood deprivation, applied at Lower Super Output Area level (a standard national address code containing an average 1500 residents), and derived from composite national Census measures. This was divided in quartiles ranging from low (1) to high (4) deprivation. The presence of paranoia, auditory hallucinations, and persecutory delusions, three common psychotic symptoms in schizophrenia either previously suggested to strongly interact with passivity[20,34] or in the case of paranoia to constitute a syndrome distinct from that characterised by the presence of hallucinations or other delusions[24], was also ascertained for adjustment, using previously developed NLP algorithms[31].

Statistical analysis

All analyses were carried out using Stata 15.1 software. Initial descriptive analyses assessed overlap between symptoms, demographic/clinical factors and other psychotic symptoms. After describing the prevalence of outcomes by covariate, logistic regression models were assembled to investigate associations and their independence in relation to each binary outcome. Adjusted

For peer review only - http://bmjopen.bmj.com/site/about/guidelines.xhtml

R-squared was used to assess model fit. Secondary analyses studied the impact of adjusting for covariates individually to clarify potential confounding effects. Finally, Poisson regression models investigated the relationship between number of negative outcomes and number of symptoms experienced as well as between number of negative outcomes and each individual symptom. Additional linear regression was carried out to study the relationship between symptoms and number of antipsychotics prescribed, assessing the reliability of the latter as a marker of negative outcome.

Patient and public involvement

The study proposal was reviewed and approved by the patient-led CRIS oversight committee prior to project commencement. No other consultations were made with patients or the public.

RESULTS

Table 1 describes symptom prevalence levels by demographic and clinical groups. Thought interference and somatic passivity were more common in younger patients and marginally higher in men compared to women but showed no consistent associations with ethnicity or neighbourhood deprivation. Both symptoms occurred more often in the presence of other psychotic symptoms but did not differ substantially between comparison diagnostic groups. Descriptive results indicated that the symptoms of interest overlapped. This was further analysed by describing the co-occurrence of symptoms and later analysing the correlation between this and negative outcomes. Overlaps between subcomponents of thought interference are summarised in Supplementary table 2, and Supplementary table 3 describes overlap between passivity, thought interference and the other positive symptoms included as covariates.

For peer review only - http://bmjopen.bmj.com/site/about/guidelines.xhtml

Table 1: symptom prevalence by demographic characteristics and diagnoses, and overlap with other psychotic symptoms

		None (%)	Interference without passivity (%)	*Broadcast (%)*	*Insertion (%)*	*Withdrawal (%)*	Passivity without interference (%)	Interference and passivity (%)	Total
	Total participants	77.0	12.8	10.3	10.7	4.9	4.9	5.2	9,323
Age at diagnosis	16-25	65.8	19.3	*16.1*	*17.4*	*7.8*	6.3	8.5	2,131
	26-35	72.4	14.8	*12.7*	*12.5*	*6.0*	5.9	6.9	2,457
	36-45	80.2	11.3	*8.6*	*8.7*	*4.4*	4.3	4.2	1,799
	46-55	82.8	10.2	*6.9*	*8.1*	*3.6*	3.8	3.2	1,241
	56-65	87.6	6.9	*4.1*	*4.8*	*2.0*	3.8	1.7	651
	66-75	91.0	4.8	*3.0*	*4.0*	*0.5*	2.6	1.6	568
	76-85	93.5	3.4	*2.6*	*1.6*	*1.0*	2.9	0.3	383
	86-95	92.5	6.5	*5.4*	*2.2*	*0.0*	1.1	0.0	93
Gender	Female	80.3	11.2	*8.5*	*10.2*	*3.8*	4.4	4.2	3,920
	Male	74.7	14.1	*11.5*	*11.1*	*5.7*	5.3	6.0	5,399
Ethnicity	Black	74.9	14.1	*11.2*	*11.7*	*5.5*	5.4	5.7	3,065
	South Asian	76.1	16.3	*12.0*	*11.6*	*7.3*	2.7	5.0	301
	Chinese	72.4	9.2	*10.5*	*7.9*	*2.6*	10.5	7.9	76
	Other Asian	72.7	13.3	*13.7*	*12.3*	*6.8*	3.8	10.2	293
	White	78.3	12.4	*10.1*	*10.3*	*4.4*	4.6	4.8	3,631
	Other/unknown	79.0	11.4	*8.3*	*9.6*	*4.3*	5.0	4.6	1,957
Index of Multiple Deprivation (IMD)	IMD quartile 1	78.1	12.7	*10.5*	*9.9*	*4.7*	4.4	4.8	2,331
	IMD quartile 2	77.5	12.1	*9.7*	*10.3*	*4.5*	5.1	5.3	2,331
	IMD quartile 3	75.7	13.1	*10.5*	*11.7*	*5.2*	5.3	5.8	2,338
	IMD quartile 4	76.8	13.4	*10.3*	*10.8*	*5.4*	4.8	5.0	2,323
Diagnosis	Schizophrenia diagnosis (F20)	78.2	12.0	*9.8*	*9.5*	*4.7*	4.9	4.9	3,475
	schizotypal/non-mood psychotic disorder (F21-29)	75.3	13.4	*10.5*	*11.4*	*5.0*	4.9	6.5	5,848
Other psychotic symptom	Paranoia present	73.2	15.0	*12.2*	*12.5*	*5.7*	5.7	6.2	7,478
	Paranoia absent	92.7	4.5	*2.5*	*3.5*	*1.8*	1.7	1.1	1,845
	Auditory Hallucinations present	65.8	18.4	*16.2*	*16.7*	*7.3*	6.9	9.0	4,971
	Auditory Hallucinations absent	89.9	6.5	*3.5*	*3.8*	*2.2*	2.6	1.0	4,352
	Persecutory Delusions present	66.2	18.1	*15.6*	*16.4*	*7.0*	7.3	8.4	4,375
	Persecutory Delusions absent	86.0	8.2	*5.6*	*5.6*	*3.1*	2.8	2.4	4984

Using logistic regression, odds ratios (OR) were calculated to further study overlap between symptoms (Supplementary table 4), reiterating increased prevalence of thought interference and somatic passivity when other symptoms are present. Thought insertion and broadcasting were strongly correlated (OR=11.67) as were insertion and withdrawal (OR=12.10), whereas there was a weaker association between broadcast and withdrawal (OR=6.05). Somatic passivity was relatively strongly associated with broadcast (OR=5.13) and insertion (OR=6.66) but was more strongly associated with other psychotic symptoms such as paranoia (OR=4.67) and auditory hallucinations (OR=4.99) than with thought withdrawal (OR=3.86).

Table 2 describes outcome distributions. Younger patients, patients from Black ethnic groups and patients from higher deprivation neighbourhoods were at greater risk of negative outcomes. Patients with schizophrenia were less likely to have an MHA section or higher time in crisis care than those with other diagnoses but more likely to be prescribed multiple antipsychotics. Negative outcomes were higher in patients with reported paranoia, auditory hallucinations or persecutory delusions, and in those with thought interference or somatic passivity. As mentioned previously, based on past descriptions of symptoms as co-occurring we described their overlap in supplementary tables 2 and 3. Similarly, we investigated whether our outcomes of interest occurred independently or together. Supplementary table 5 describes overlap between outcomes.

For peer review only - http://bmjopen.bmj.com/site/about/guidelines.xhtml

Table 2: Two-year incidence of negative outcomes by symptom and covariate groups

	Covariate	Any negative Outcome (%)	MHA section (%)	>2 antipsychotics prescribed (%)	>22 days spent in crisis care (%)	Total participants
Interference/ Passivity	None	42.7	31.8	21.9	10.6	7,181
	Interference without passivity	61.4	46.5	31.6	32.6	1,198
	Broadcast	*63.3*	*47.4*	*34.2*	*34.9*	*956*
	Insertion	*67.4*	*49.9*	*36.6*	*38.1*	*997*
	Withdrawal	*66.9*	*52.1*	*36.2*	*40.1*	*459*
	Passivity without interference	68.2	49.8	36.4	35.7	456
	Interference and passivity	72.3	54.5	42.2	43.2	488
	Total participants	47.9	35.8	24.9	25.0	9,323
Age at diagnosis	16-25	61.0	48.6	32.7	36.8	2,131
	26-35	52.8	40.7	27.0	26.1	2,457
	36-45	55.3	32.9	22.5	21.2	1,799
	46-55	54.1	31.7	23.8	20.9	1,241
	56-65	33.5	21.8	17.8	16.9	651
	66-75	29.9	17.8	15.7	16.2	568
	76-85	24.3	15.4	11.7	13.3	383
	86-95	24.7	14.0	14.0	12.9	93
Gender	Female	46.9	35.2	25.1	24.2	3,920
	Male	48.6	36.2	24.8	25.6	5,399
Ethnicity	Black	57.5	47.6	30.0	31.3	3,065
	South Asian	43.5	30.2	24.9	19.3	301
	Chinese	50.0	44.7	15.8	21.1	76
	Other Asian	48.1	36.2	27.3	23.9	293
	White	43.9	31.1	23.3	22.1	3,631
	Other/unknown	40.7	26.4	20.0	21.7	1,957
Index of Multiple Deprivation (IMD)	IMD quartile 1	45.6	32.9	24.5	23.1	2,331
	IMD quartile 2	46.3	33.6	25.0	24.2	2,331
	IMD quartile 3	50.3	38.1	25.8	26.6	2,338
	IMD quartile 4	49.2	38.4	24.3	26.1	2,323
Diagnosis	Schizophrenia diagnosis (F20)	44.4	30.6	29.9	22.2	3,475
	schizotypal/non-mood psychotic disorder (F21-29)	49.9	38.8	22.0	26.7	5,848
Other symptoms	Paranoia present	53.9	41.0	27.7	28.9	7,478
	Paranoia absent	23.6	14.5	13.5	9.4	1,845
	Auditory Hallucinations present	58.2	43.8	31.3	31.7	4,971
	Auditory Hallucinations absent	36.0	26.6	17.6	17.3	4,352
	Persecutory Delusions present	62.2	49.2	31.9	34.4	4,375
	Persecutory Delusions absent	35.7	23.7	18.6	16.6	4984

For peer review only - http://bmjopen.bmj.com/site/about/guidelines.xhtml

Table 3 summarises logistic regression analyses for any negative outcome. Unadjusted models showed significant associations with somatic passivity and all components of thought interference, and these remained significant after adjustments except thought broadcast. Although confidence intervals overlapped, associations with somatic passivity were consistently strongest. Post-hoc investigations of those with non-overlapping somatic passivity and thought interference showed a significant association with somatic passivity (OR=1.57, 95% CI 1.27-1.95) but not with thought interference (OR=1.11, 95% CI 0.97-1.27).

For peer review only - http://bmjopen.bmj.com/site/about/guidelines.xhtml

Table 3: Unadjusted and adjusted odds ratio for the occurrence of any negative outcome in the presence of thought interference and somatic passivity

Outcome: any negative outcome	**Unadjusted OR (95% CI)**	**Adjusted* OR (95% CI)**	**Adjusted model pseudo R-squared**
Somatic passivity	2.86 (2.47-3.31)	1.61 (1.37-1.88)	0.12
Interference	2.30 (2.07-2.57)	1.25 (1.11-1.41)	0.12
Broadcast	2.01 (1.75-2.31)	1.05 (0.91-1.22)	0.12
Insertion	2.47 (2.15-2.84)	1.24 (1.15-1.55)	0.12
Withdrawal	2.29 (1.88-2.79)	1.36 (1.10-1.69)	0.12
Interference or passivity	2.54 (2.29-2.80)	1.30 (1.19-1.42)	0.12
Both interference and passivity	1.73 (1.57-1.92)	1.24 (1.11-1.38)	0.12
Post-hoc: passivity without interference	2.44 (1.99-2.98)	1.57 (1.27-1.95)	0.12
Post-hoc: interference without passivity	1.88 (1.66-2.13)	1.11 (0.97-1.27)	0.12

*Adjusted for age at diagnosis, gender, ethnicity, diagnosis, IMD quartile and presence of paranoia, auditory hallucinations, persecutory delusion.
Abbreviations: OR = odds ratio; RR = risk ratio

Table 4 displays further analyses of individual outcomes. Associations with somatic passivity remained significant for all after adjustments. Thought interference and insertion specifically were independently associated with higher time spent in crisis care and greater numbers of antipsychotics used, but not with MHA sections. Thought broadcast specifically was not independently associated with any outcome, and thought withdrawal was only associated with higher time spent in crisis care and MHA sections. Aside from thought withdrawal, all other symptoms had the weakest association with MHA sections.

For peer review only - http://bmjopen.bmj.com/site/about/guidelines.xhtml

Table 4: Unadjusted and adjusted odds ratios for associations of each negative outcome with the presence of somatic passivity, thought interference, broadcasting, insertion and withdrawal

**Exposure: somatic passivity**	**Unadjusted OR (95% CI)**	**Adjusted* OR (95% CI)**	**Adjusted model pseudo R-squared**
Any negative outcome	2.86 (2.47-3.31)	1.61 (1.37-1.88)	0.12
>22 days spent in crisis care	2.15 (1.87-2.48)	1.33 (1.15-1.54)	0.08
MHA section	2.13 (1.86-2.44)	1.23 (1.06-1.42)	0.13
>2 antipsychotics prescribed	2.14 (1.86-2.46)	1.43 (1.23-1.66)	0.07
**Exposure: thought interference**			
Any negative outcome	2.30 (2.07-2.57)	1.25 (1.11-1.41)	0.12
>22 days spent in crisis care	1.89 (1.69-2.12)	1.13 (1.00-1.28)	0.08
MHA section	1.95 (1.75-2.17)	1.08 (0.96-1.21)	0.13
>2 antipsychotics prescribed	1.80 (1.60-2.01)	1.16 (1.02-1.31)	0.07
**Exposure: thought broadcasting**			
Any negative outcome	2.01 (1.75-2.31)	1.05 (0.91-1.22)	0.12
>22 days spent in crisis care	1.71 (1.49-1.97)	1.01 (0.87-1.18)	0.08
MHA section	1.71 (1.50-1.96)	0.92 (0.80-1.07)	0.13
>2 antipsychotics prescribed	1.66 (1.44-1.91)	1.05 (0.90-1.22)	0.07
**Exposure: thought insertion**			
Any negative outcome	2.47 (2.15-2.84)	1.34 (1.15-1.56)	0.12
>22 days spent in crisis care	2.01 (1.75-2.31)	1.21 (1.05-1.40)	0.08
MHA section	1.93 (1.69-2.20)	1.06 (0.92-1.23)	0.13
>2 antipsychotics prescribed	1.88 (1.64-2.16)	1.22 (1.05-1.41)	0.07
**Exposure: thought withdrawal**			
Any negative outcome	2.29 (1.88-2.79)	1.36 (1.10-1.69)	0.12
>22 days spent in crisis care	2.09 (1.73-2.54)	1.37 (1.12-1.68)	0.08
MHA section	2.02 (1.68-2.44)	1.23 (1.01-1.51)	0.13
>2 antipsychotics prescribed	1.76 (1.45-2.14)	1.21 (0.98-1.48)	0.07

*Adjusted for age at diagnosis, gender, ethnicity, diagnosis, IMD quartile and presence of paranoia, auditory hallucinations, persecutory delusion.
Abbreviations: OR = odds ratio; MHA = Mental Health act

For peer review only - http://bmjopen.bmj.com/site/about/guidelines.xhtml

Table 5 summarises analyses investigating adjustments by individual covariates. For both somatic passivity and thought interference, strongest reductions in OR occurred following adjustments for age and the other three psychotic symptoms; however, OR for somatic passivity remained stronger throughout. Estimated marginal means (EM) were calculated to determine the likelihood of negative outcomes at different ages (Supplementary Table 6); this showed a decrease in risk inversely proportional to age: for a patient with somatic passivity, at age 20 EM=59.6(58.0-61.1)%; at age 90, EM=21.5(19.2-23.8)%. For a patient with thought interference, at age 20 EM=59.1(57.6-60.7)%; at age 90, EM=22.2(19.8-24.6)%. Finally, exploratory testing investigating the cumulative relationship between symptoms and outcomes, calculating the odds of experiencing multiple negative outcomes in relation to the sum of individual symptoms and to each individual symptom and illustrated using EM, described a 14.9% increase in the risk of multiple negative outcomes when experiencing no versus all four symptoms, from 73.1% to 88.0%. Consistently with identified trends, somatic passivity was the individual symptom with the greatest impact (adjusted OR=1.16(1.09-1.23)) and thought broadcasting had no significant effect after adjustment (OR=0.99(0.92-1.05)). Regression analysis identified a significant interaction between somatic passivity and number of antipsychotics ($p<0.05$; adjusted R-squared=0.1031). Thought interference and its subcomponents, also included in the model, had no significant effect (supplementary table 7).

For peer review only - http://bmjopen.bmj.com/site/about/guidelines.xhtml

Table 5: Logistic regression analysis of associations with any negative outcome adjusting separately for individual covariates

Outcome: Any negative outcome	**Somatic Passivity**	**Thought Interference**
Unadjusted	2.86 (2.47-3.31)	2.30 (2.07-2.57)
Adjusted for age at diagnosis	2.47 (2.13-2.86)	1.94 (1.73-2.17)
Adjusted for gender	2.85 (2.46-3.30)	2.30 (2.06-2.57)
Adjusted for ethnicity	2.85 (2.46-3.30)	2.28 (2.04-2.55)
Adjusted diagnosis	2.86 (2.47-3.31)	2.30 (2.06-2.56)
Adjusted for IMD quartile	2.86 (2.47-3.31)	2.30 (2.06-2.57)
Adjusted paranoia	2.43 (2.09-2.82)	1.94 (1.74-2.17)
Adjusted for auditory hallucinations	2.23 (1.92-2.59)	1.80 (1.61-2.02)
Adjusted for persecutory delusions	2.23 (1.91-2.59)	1.82 (1.63-2.04)

For peer review only - http://bmjopen.bmj.com/site/about/guidelines.xhtml

DISCUSSION

Having developed NLP algorithms to extract recorded thought interference and somatic passivity in routine electronic mental health records, we investigated their associations with negative outcomes over a 2-year period following first-recorded diagnosis of schizophrenia or a schizophreniform disorder. Both symptoms were associated with a significantly increased risk of experiencing negative outcomes, somatic passivity having the strongest associations after full adjustment. This is broadly consistent with previous reports that experiencing these symptoms is predictive of negative outcomes: Stephen et al found the absence of somatic passivity to correlate with better outcomes in 66% of patients[8]; Koehler found no significant difference in outcomes between patients experiencing any thought interference subcomponent or somatic passivity but concluded that their absence was predictive of a better prognosis[9]. In 2019, Malinowski et al found that thought broadcasting seemed predictive of disease continuation without remission, but that this effect was no longer significant after adjusting for external factor influence (specifically, untreated psychosis duration and drug abuse)[35]; accordingly, although different covariates were included, our results suggest that thought broadcast does not have a significant effect on outcome. Similar prevalence and overlap between thought insertion, withdrawal and broadcasting suggests that this cannot be due to broadcasting occurring more frequently in isolation. It is interesting to note that aside from broadcasting, these symptoms remained significantly associated with negative outcomes after including common non-first-rank symptoms of schizophrenia in our covariates, namely paranoia, auditory hallucinations and persecutory delusions, suggesting the importance of their link with negative outcomes. Secondary testing identified a unique significant effect of somatic passivity on number of antipsychotics prescribed, possibly reflecting poorer response leading to worsened outcomes; importantly, this outcome may reflect a degree of treatment resistance

For peer review only - http://bmjopen.bmj.com/site/about/guidelines.xhtml

not investigated here but likely to influence patient prognosis. Our findings suggest that somatic passivity and thought interference subcomponents correlate with an increased risk of experiencing negative outcomes, some more strongly than others. In contrast, it is interesting to note that no particular outcome appeared to be disproportionately correlated with symptoms. Although the odds ratio of an MHA section being in place were less strong than other outcomes, this reflects that their use is reserved for the most unwell patients who refuse or lack capacity to accept voluntary community treatment. Overall, the odds of all three outcomes increased in a similar fashion in the presence of thought interference and somatic passivity, reflecting a generally worsened prognosis.

Nuevo et al described schizophrenia symptoms as a continuum whereby the experience of multiple symptoms predicts a worse prognosis[10]. Our finding that patients with either or both thought interference and somatic passivity were more likely to experience negative outcomes than those for whom these symptoms were absent supports this. Exploratory analyses suggested a significant relationship between number of symptoms experienced and negative outcome occurrence; however, our results also suggest that each symptom has a different impact on outcomes which was not always individually significant, and that the presence of multiple symptoms may even sometimes reduce the frequency of negative outcomes: for example, patients experiencing somatic passivity only may have poorer outcomes than those with both this and thought interference. One explanation may be that reporting thought interference to clinicians requires greater insight from the patient, contributing to better outcomes and facilitating voluntary treatment such as that offered by crisis care teams with which more patients experiencing thought insertion and withdrawal engaged. The recording of exposure variables relied on symptoms being elicited and noted by clinicians in routine practice and therefore cannot be assumed to be equivalent to those ascertained in a more research-

focused interview, just as covariates such as diagnosis also were derived from routine clinical records and should not be assumed to equate research diagnostic criteria; however, we feel that inaccuracies in exposure ascertainment were more likely to obscure findings of interest through non-differential measurement error rather than give rise to spurious associations. Unfortunately, although mandatory hospitalisation under the MHA reflects a need for crisis care, it is not possible with these algorithms to determine what interventions patients received and responded to in that context specifically. However, guideline consistency since 2007 suggests that patients represented in this analysis should have received similar standards of care across the time period sampled. Overall, although there does seem to be a trend of worsening outcomes as symptoms accumulate, it would be overly simplistic to claim that this effect is purely additive.

Interestingly, of the covariates investigated, only age had a substantial confounding effect, negatively correlated with symptoms and outcomes of interest. In the past, thought insertion and withdrawal have been reported as more common in early-onset cases and somatic passivity as more frequent in late-onset cases[11]. Because of challenges in establishing whether patients were previously diagnosed in another service before presenting at SLaM, it is not possible to equate first presentation here with first-episode psychosis, particularly in older individuals who may have longer standing diagnoses and recently in-migrated to the catchment. This first presentation sample is therefore better viewed as one which is enriched with first-episode cases rather than directly equivalent. Furthermore, as our database consists of real-life data patients do not always have consistent follow-up or repeat assessments, including due to external factors not related to the patient's care. Our model is therefore useful in correlating patient outcomes with symptoms at first presentation but it is not possible to infer stability of the clinical construct over time as the patient is only assessed once. Although age at diagnosis may

For peer review only - http://bmjopen.bmj.com/site/about/guidelines.xhtml

not accurately reflect age at disease onset and doesn't account for possible changes in symptom profile as patients age, our results suggest that both symptoms are more frequent in younger patients, leading to a worsened prognosis. In this context, it would be interesting, in future research, to investigate symptom stability over time and how prognosis might also change with this.

Other external factors such as level of education or spiritual beliefs may play a role in the personal interpretation of abnormal experiences and have consequently affected outcomes. We developed a model that suggested the existence of significant interactions between thought interference, somatic passivity and the occurrence of negative outcomes. As shown in supplementary tables 2 and 3, these symptoms overlapped in many patients as well as with other psychotic symptoms, and so did the negative outcomes under scrutiny. The overlap observed before further analyses suggested that there may exist interactions linking these factors and directed our analyses towards the development of a model to understand these better. The algorithms we developed to create this model achieved high performance metrics and our model-fit measures demonstrate model reliability but highlight that the variance remains in great part unexplained, likely due to the role of other factors not included here. This could be usefully explored in further studies to refine models assessing patient prognosis. Furthermore, our patient sample was drawn from an inner-urban South-London population and results may be different in other patient groups, although the large sample size should mitigate this effect.

Paranoia, persecutory delusions and auditory hallucinations were included as covariates due to past evidence suggesting a correlation with thought interference and somatic passivity. The primary purpose of this study was to characterise symptoms of thought interference and

For peer review only - http://bmjopen.bmj.com/site/about/guidelines.xhtml

passivity, and other symptoms, such as negative and disorganised symptoms, were not included. Broader symptom profiles should be investigated in future research. Interactions between outcomes should also be studied; for example, it is not unreasonable to propose that the prescription of fewer antipsychotics may correlate with reduced hospitalisation time. To this effect, a harmonisation of definitions would be useful to enable the development of algorithms studying symptoms described more heterogeneously. Outcomes analysed were restricted to those most readily available from the source data and most likely to be clinically informative; however, they cannot be viewed as exhaustive and there may be elements of prognosis that were not captured in this study, including interactions between outcomes and previous adverse events that may influence symptom occurrence and recording.

Although to be treated with caution, as not tested for significance, our descriptive results provide potential directions for future research and support the notion that psychotic symptoms share more complex interactions than previously described. Somatic passivity has been suggested to cluster with different symptoms such as persecutory delusions[4] or auditory-verbal hallucinations[5], and thought interference has sometimes been described as a component of somatic passivity[18&36]. We found that subcomponents of thought interference traditionally assumed to closely correlate may actually more commonly occur alongside other symptoms; e.g., the odds of experiencing thought insertion alongside somatic passivity were greater than those of withdrawal and broadcasting co-occurring. In a study attempting to link delusions to putative underlying neurobiological mechanisms, Kimhy et al called for new symptom definitions that would focus on underlying mechanisms rather than phenomenological descriptions[23]. Each delusion was described then grouped with others based on correlation, enabling the grouping of symptoms that share significant interactions while maintaining their individuality. This approach enables a less biased study of psychotic symptoms relying less on

For peer review only - http://bmjopen.bmj.com/site/about/guidelines.xhtml

traditional groupings and may allow more accurate descriptions of symptom interactions. Additionally, thought interference and somatic passivity have previously been found to be closely related and predictive of schizophrenia[13&36-38]. Our results support reports that these symptoms share a positive correlation[20] but question their specificity to schizophrenia and usefulness in differentiating this from other psychotic disorders, particularly as both were absent in 77% of patients. Somatic passivity has been reported to be the most common symptom experienced in schizophrenia[6] and first-episode psychosis[24]; however, in our study thought interference was almost twice as commonly recorded. Overall, it would be interesting to repeat these analyses in the future with a broader range of diagnoses including affective psychoses in which they also occur to determine whether these symptoms could be more specific to other conditions or used to assess patient prognosis.

CONCLUSION

This study developed NLP applications to detect the recorded presence of somatic passivity, thought interference and its subcomponents in patients within three months of their first presentation and analysed negative outcome occurrence over the two following years to investigate the relationship between these specific FRS and patient prognosis. We observed a significant effect of somatic passivity, thought interference and its subcomponents thought insertion and withdrawal on the likelihood of experiencing any negative outcome. We also offer evidence that psychotic symptoms traditionally associated with schizophrenia may not be specific to this disorder and share complex interactions, putting in question current definitions and warranting further investigation to determine whether it is correct to consider thought insertion, withdrawal and broadcasting as parts of the same symptom. Overall, the presence of somatic passivity and thought interference was found to significantly increase the risk of experiencing negative outcomes in psychosis; somatic passivity had the greatest effect. Future

For peer review only - http://bmjopen.bmj.com/site/about/guidelines.xhtml

research should focus on acquiring a better understanding of these effects, including by investigating other psychotic symptoms to determine the more detailed pathways through which these interact with prognosis in schizophrenia and other types of psychosis. Future studies should investigate these correlations and reliable markers of outcomes to develop standardised tools enabling a better evaluation of patient prognosis. In addition, it would be interesting to study which antipsychotics specifically were prescribed and whether patients may have had a better response to specific medication, reflective of a general lack of response to individual antipsychotics in schizophrenia rather than the presence of refractory disease.

For peer review only - http://bmjopen.bmj.com/site/about/guidelines.xhtml

Funding

RS, RP and AK are part-funded by the National Institute for Health Research (NIHR) Biomedical Research Centre at the South London and Maudsley NHS Foundation Trust and King's College London. RS is additionally part-funded by: i) an NIHR Senior Investigator Award; ii) the National Institute for Health Research (NIHR) Applied Research Collaboration South London (NIHR ARC South London) at King's College Hospital NHS Foundation Trust; iv) the DATAMIND HDR UK Mental Health Data Hub (MRC grant MR/W014386). The views expressed are those of the authors and not necessarily those of the NIHR or the Department of Health and Social Care. RP has received funding from an NIHR Advanced Fellowship (NIHR301690), a Medical Research Council (MRC) Health Data Research UK Fellowship (MR/S003118/1) and a Starter Grant for Clinical Lecturers (SGL015/1020) supported by the Academy of Medical Sciences, The Wellcome Trust, MRC, British Heart Foundation, Arthritis Research UK, the Royal College of Physicians and Diabetes UK.

Competing interests

RS declares research support received in the last 36 months from Janssen, GSK and Takeda. RP has received grant funds from Janssen and consultancy fees from Induction Healthcare and Holmusk.

Contributors

TM, AK, JS, RP and RS planned the study together. JS developed the applications, which were doubly annotated by TM and AK. JS and AK retrieved the data, and TM analysed it with advice from AK, JS, RP and RS. TM wrote the largest part of the paper, produced the tables and figure and submitted it, working under supervision from AK and RS. AK, RP and RS commented on and directly contributed to the manuscript.

For peer review only - http://bmjopen.bmj.com/site/about/guidelines.xhtml

Ethics statement

The source de-identified data are made available for secondary analyses as approved by Oxford Research Ethics Committee C, reference 18/SC/0372.

Data availability statement

No additional data are available.

REFERENCES

1. Schneider, K. *Klinische Psychopathologie* (5th edn) (Trans. Hamilton). New York: Grune & Stratton; 1959.
2. Chapman LJ, Chapman JP. Scales for Rating Psychotic and Psychotic-like Experiences as Continua. *Schizophrenia Bull*. 1980;6(3):476-489 doi: 10.1093/schbul/6.3.476 [published in print First: January 1980]
3. Chapman LJ, Edell WS, Chapman JP. Physical Anhedonia, Perceptual Aberration, and Psychosis Proneness. *Schizophrenia Bull*. 1980;6(4):639-653 doi: 10.1093/schbul/6.3.476 [published in print First: January 1980]
4. Kwapil TR, Chapman LJ, Chapman J. Validity and Usefulness of the Wisconsin Manual for Assessing Psychotic-like Experiences. *Schizophrenia Bull*. 1999;25(2):363-375 doi: 10.1093/oxfordjournals.schbul.a033384 [published in print First: 1999]
5. Landmark J, Merskey H, Cernovsky Z, et al. The positive triad of schizophrenic symptoms. Its statistical properties and its relationship to 13 traditional diagnostic systems. *Br J Psychiatry J Ment Sci*. 1990;156(3):388-394. doi: 10.1192/bjp.156.3.388 [published Online First: July 2011]
6. Bleuler, E. *Dementia Praecox oder Gruppe der Schizophrenien*. Leipzig: Germany Deuticke; 1911.
7. Cloninger CR, Martin RL, Guze SB, et al. Diagnosis and Prognosis in Schizophrenia. *Arch Gen Psychiat*. 1985;42(1):15 doi: 10.1001/archpsyc.1985.01790240017002 [published in print First: January 1985]
8. Loftus J, Delisi LE, Crow TJ. Factor structure and familiality of first-rank symptoms in sibling pairs with schizophrenia and schizoaffective disorder. *Brit J Psychiat*. 2000;177(1):15-19 doi: 10.1192/bjp.177.1.15 [published Online First: July 2000]

9. Fierro M, Hernández AM, Malcolm J. The Psychopathology of Delusion of Control According to Subjective Experience. *Revista Colombiana De Psiquiatría*. 2018;47(4):221-228 doi:10.1016/j.rcpeng.2018.10.005 [published Online First: December 2018]

10. Rosen C, Jones N, Chase KA, et al. The intrasubjectivity of self, voices and delusions: A phenomenological analysis. *Psychos*. 2016;8(4):357-368 doi: 10.1080/17522439.2016.1162839 [published Online First: April 2016]

11. Malik SB, Ahmed M, Bashir A, et al. Schneider's First-Rank Symptoms of Schizophrenia: Prevalence and Diagnostic Use. *Brit J Psychiat*. 1990;156(1):109-111 doi: 10.1192/bjp.156.1.109 [published in print First: January 1990]

12. American Psychiatric Association. *Diagnostic and Statistical Manual of Mental Disorders, Fifth Edition (DSM-5)*. Washington, DC: American Psychiatric Association; 2013.

13. Kendler KS. Phenomenology of Schizophrenia and the Representativeness of Modern Diagnostic Criteria. *Jama Psychiat*. 2016;73(10):1082 doi:10.1001/jamapsychiatry.2016.1976 [published Online First: October 2016]

14. Nemeroff CB, Weinberger D, Rutter M, et al. DSM-5: a collection of psychiatrist views on the changes, controversies, and future directions. *Bmc Med*. 2013;11(1):202 doi: 10.1186/1741-7015-11-202 [published Online First: September 2013]

15. Ullrich S, Keers R, Coid JW. Delusions, anger, and serious violence: new findings from the MacArthur Violence Risk Assessment Study. *Schizophrenia Bull*. 2013;40(5):1174-1181 doi: doi.org/10.1093/schbul/sbt126 [published Online First: September 2013]

16. Stephens JH, Richard P, McHugh PR. Long-Term Follow-up of Patients Hospitalized for Schizophrenia, 1913 to 1940. *J Nerv Amp Ment Dis*. 1997;185(12):715-721.

17. Koehler K. Prognostic Prediction in RDC Schizo-Affective Disorder on the Basis of First-Rank Symptoms Weighted in Terms of Outcome. *Psychopathology*. 1983;16(2-4):186-197 doi: 10.1159/000283967 [published in print First: 1983]

For peer review only - http://bmjopen.bmj.com/site/about/guidelines.xhtml

18. Nuevo R, Chatterji S, Verdes E, et al. The continuum of psychotic symptoms in the general population: a cross-national study. *Schizophrenia Bull*. 2010;38(3):475-485 doi: 10.1093/schbul/sbq099 [published Online First: September 2010]

19. Howard R, Castle D, Wessely S, et al. A Comparative Study of 470 Cases of Early-Onset and Late-Onset Schizophrenia. *Brit J Psychiat*. 1993;163(3):352-357 doi: 10.1192/bjp.163.3.352 [published in print First: September 1983]

20. Alici-Evcimen Y, Ertan T, Eker E. Case Series With Late-Onset Psychosis Hospitalized in a Geriatric Psychiatry Unit in Turkey: Experience in 9 Years. *Int Psychogeriatr*. 2003;15(1):69-72 doi: 10.1017/S1041610203008767 [published Online First: March 2003]

21. Naqvi HA, Hussain S, Islam M, et al. Early psychosis symptoms. *J Coll Physicians Surg Jcpsp*. 2014;24(3):198-202.

22. Stanghellini G, Monti MR. Influencing and being influenced: the other side of "bizarre delusions". 2. Clinical investigation. *Psychopathology*. 1993;26(3-4):165-169 doi: 10.1159/000284817 [published in print First: February 1993]

23. Kimhy D, Goetz R, Yale S, et al. Delusions in Individuals with Schizophrenia: Factor Structure, Clinical Correlates, and Putative Neurobiology. *Psychopathology*. 2005;38(6):338-344 doi: 10.1159/000089455 [published Online First: December 2005]

24. Fernandes AC, Cloete D, Broadbent MTM, et al. Development and evaluation of a de-identification procedure for a case register sourced from mental health electronic records. *Bmc Med Inform Decis*. 2013;13(1):71 doi: 10.1186/1472-6947-13-71 [published Online First: July 2013]

25. Stuart GW, Malone V, Currie J, et al. Positive and negative symptoms in neuroleptic-free psychotic inpatients. *Schizophr Res*. 1995;16(3):175-188 doi: 10.1016/0920-9964(94)00083-K [published in print First: August 1995]

For peer review only - http://bmjopen.bmj.com/site/about/guidelines.xhtml

26. Patel R, Irving J, Brinn A, *et al.* Impact of the COVID-19 pandemic on remote mental healthcare and prescribing in psychiatry: an electronic health record study. *Bmj Open* 2021;**11**:e046365. doi:10.1136/bmjopen-2020-046365.

27. South London and Maudsley NHS Foundation Trust. Our Services, Types of service. https://slam.nhs.uk/our-services/types-of-service/ (accessed 21 Feb. 2022).

28. Stewart R, Soremekun M, Perera G, et al. The South London and Maudsley NHS Foundation Trust Biomedical Research Centre (SLAM BRC) case register: development and descriptive data. *Bmc Psychiatry*. 2009;9(1):51 doi: 10.1186/1471-244X-9-51 [published Online First: August 2009]

29. Perera G, Broadbent M, Callard F, et al. Cohort profile of the South London and Maudsley NHS Foundation Trust Biomedical Research Centre (SLaM BRC) Case Register: current status and recent enhancement of an Electronic Mental Health Record-derived data resource. *Bmj Open*. 2016;6(3):e008721 doi: 10.1136/bmjopen-2015-008721 [published Online First: March 2016]

30. CRIS NLP Service. CRIS Natural Language Processing Applications Library (2020), v1.2. Maudsley Biomedical Research Centre. https://www.maudsleybrc.nihr.ac.uk/facilities/clinical-record-interactive-search-cris/cris-natural-language-processing/. Updated 12/06/20. Accessed 03/08/20.

31. Jackson RG, Patel R, Jayatilleke N, et al. Natural language processing to extract symptoms of severe mental illness from clinical text: the Clinical Record Interactive Search Comprehensive Data Extraction (CRIS-CODE) project. *Bmj Open*. 2017;7(1):e012012 doi: 10.1136/bmjopen-2016-012012 [published Online First: January 2017]

32. Jackson RG, Ball M, Patel R, et al. TextHunter--A User Friendly Tool for Extracting Generic Concepts from Free Text in Clinical Research. *Amia Annu Symposium Proc Amia*

For peer review only - http://bmjopen.bmj.com/site/about/guidelines.xhtml

Symposium Amia Symposium. 2014;2014:729-738 doi: 10.13140/2.1.3722.9121 [published Online First: November 2014]

33. World Health Organisation. *International Statistical Classification of Diseases and Related Health Problems, 10th Revision (ICD-10)*. Geneva: WHO; 1992.
34. Galdos P, Os J van. Gender, psychopathology, and development: from puberty to early adulthood. *Schizophr Res*. 1995;14(2):105-112 doi: 10.1016/0920-9964(94)00020-9 [published in print First: January 1995]
35. Malinowski FR, Tasso B de C, Ortiz BB, et al. Schneider's first-rank symptoms as predictors of remission in antipsychotic-naive first-episode psychosis. *Revista Brasileira De Psiquiatria Sao Paulo Braz 1999*. 2019;42(AHEAD):22-26 doi: 10.1590/1516-4446-2018-0237 [published Online First: February 2020]
36. Klosterkötter J, Hellmich M, Steinmeyer EM, et al. Diagnosing Schizophrenia in the Initial Prodromal Phase. *Arch Gen Psychiat*. 2001;58(2):158 doi: 10.1001/archpsyc.58.2.158 [published Online First: February 2001]
37. Moller P, Husby R. The Initial Prodrome in Schizophrenia: Searching for Naturalistic Core Dimensions of Experience and Behavior. *Schizophrenia Bull*. 2000;26(1):217-232 doi: 10.1093/oxfordjournals.schbul.a033442 [published Online First: January 2000]
38. Galdos P, Os J van. Gender, psychopathology, and development: from puberty to early adulthood. *Schizophr Res*. 1995;14(2):105-112 doi: 10.1016/0920-9964(94)00020-9 [published in print First: January 1995]

For peer review only - http://bmjopen.bmj.com/site/about/guidelines.xhtml

Figure legend

Figure 1: Flowchart illustrating the development of algorithms via the TextHunter platform

TextHunter allows a training dataset of text strings containing wording of interest to be presented in an easy visual form for researcher annotation. The development process is identical for all algorithms relating to symptoms recorded in clinical text.

For peer review only - http://bmjopen.bmj.com/site/about/guidelines.xhtml

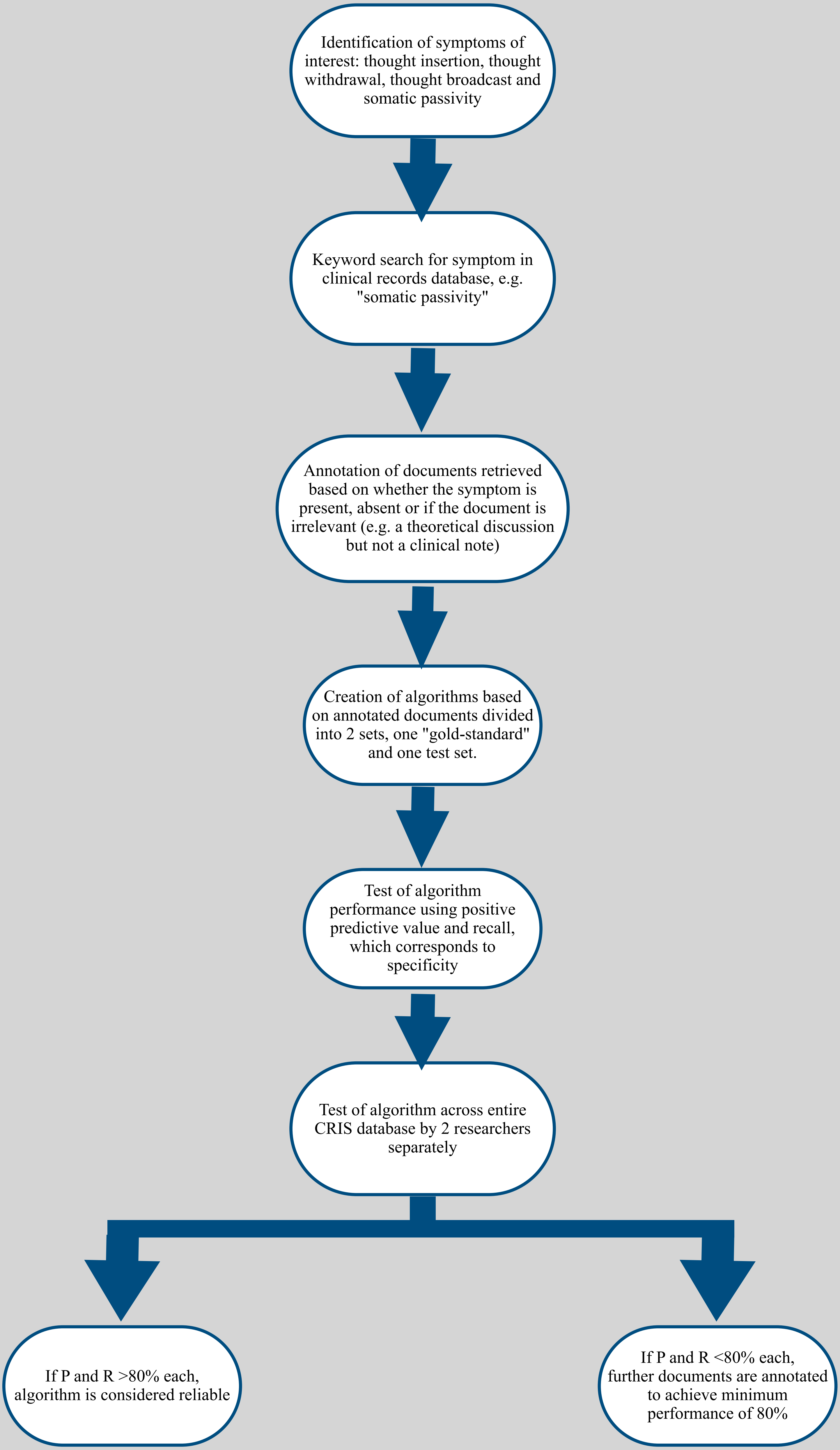
Identification of symptoms of interest: thought insertion, thought withdrawal, thought broadcast and somatic passivity
Keyword search for symptom in clinical records database, e.g. "somatic passivity"
Annotation of documents retrieved based on whether the symptom is present, absent or if the document is irrelevant (e.g. a theoretical discussion but not a clinical note)
Creation of algorithms based on annotated documents divided into 2 sets, one "gold-standard" and one test set.
Test of algorithm performance using positive predictive value and recall, which corresponds to specificity
Test of algorithm across entire CRIS database by 2 researchers separately
If P and R >80% each, algorithm is considered reliable
If P and R <80% each, further documents are annotated to achieve minimum performance of 80%

Supplementary Material

Supplementary Table 1: Natural language processing algorithm development and performance. A minimum P and R of 80% is required for an algorithm to be considered reliable. In the context of passivity, the lower IRR led to the inclusion of extra annotations that enabled us to reach this threshold[1].

Symptom	**Keyword**	**Number of annotations**	**IRR**	**P and R**	**Extra steps required**	**Manual testing**	**Keyword search P and R**
Thought insertion	"thought insert*"	592 (96 double-annoted)	97 %	P=81%; R=94%	N/A	P=84%	P=81%; R=96%
Thought broadcasting	"thought broadcast*"	398 (95 double-annoted)	94 %	P=81%; R=94%	N/A	P=84%	P=81%; R=96%
Thought withdrawal	"thought withdraw*"	500 (76 double-annotated)	95 %	P=74%; R=91%	159 extra annotations	P=84%	P= 90%; R=88%
Somatic passivity	"passivity"	500 (116 double-annotated)	71 %	P=72%; R=92%	597 extra annotations, form excluded	P=82%	P=89%; R=100%

[1] P: Precision (proportion of positive entities identified by the NLP algorithm that are genuine entities on manual annotation); R: Recall (proportion of true positive entities on manual annotation that are identified as such by the NLP algorithm).

Supplementary Table 2: Overlap between thought broadcasting, thought insertion and thought withdrawal. The overlap observed before further analyses suggested that there may exist interactions linking these factors and directed our analyses towards the development of a model to understand these better.

Symptom	**Number of patients experiencing the symptom**
Thought insertion	997
Thought broadcasting	956
Thought withdrawal	459
Thought insertion and broadcasting	436
Thought insertion and withdrawal	243
Thought broadcasting and withdrawal	170
Thought insertion, broadcasting and withdrawal	123

For peer review only - http://bmjopen.bmj.com/site/about/guidelines.xhtml

Supplementary table 3: overlap between somatic passivity, thought interference and any other positive symptoms (auditory hallucinations, persecutory delusions, paranoia). When carrying out analyses, other positive symptoms were not combined but each included as individual covariates. The overlap observed here suggested that there may exist interactions linking these factors and directed our analyses towards the development of a model to understand these better. This table also reflects the prevalence of other positive symptoms and the need for investigations of the possible correlation between these and outcomes in the future.

Symptom	**Number of patients experiencing the symptom**
Somatic passivity	944
Thought interference	1686
Other positive symptoms	8181
Somatic passivity and thought interference	488
Somatic passivity and other positive symptoms	935
Interference and other positive symptoms	1664
Passivity, intereference and other positive symptoms	487

For peer review only - http://bmjopen.bmj.com/site/about/guidelines.xhtml

Supplementary Table 4: Unadjusted odds ratios for the co-occurrence of psychotic symptoms across the patient sample

Symptom	Broadcast	Insertion	Withdrawal	Passivity	Paranoia	Auditory Hallucinations	Persecutory Delusions
Broadcast	1						
Insertion	11.67	1					
Withdrawal	6.05	12.10	1				
Passivity	5.13	6.66	3.86	1			
Paranoia	5.29	3.97	3.32	4.67	1		
Auditory Hallucinations	5.33	5.06	3.45	4.99	3.50	1	
Persecutory Delusions	3.13	3.30	2.41	3.40	5.30	2.80	1

Supplementary table 5: overlap between outcomes investigated. This highlights the degree of overlap between negative outcomes, suggesting a possible correlation in their occurrence.

Outcome	**Number of patients with this outcome**
Presence of a mental health act section	3334
More than 22 days spent in crisis care	2331
More than 2 antipsychotics prescribed	2323
Mental health act section and more than 22 days spent in crisis care	1888
Mental health act section and more than 2 antipsychotics prescribed	1477
More than 22 days spent in crisis care and more than 2 antipsychotics prescribed	1245
Presence of all 3 outcomes	1085

For peer review only - http://bmjopen.bmj.com/site/about/guidelines.xhtml

Supplementary Table 6: Prevalence of negative outcomes in patients with somatic passivity or thought interference at different ages

Age (years)	Occurrence on negative outcomes in patients with somatic passivity (%) (95% CI)	Occurrence on negative outcomes in patients with thought interference (%) (95% CI)
20	59.6 (58.0-61.1)	59.1 (57.6-60.7)
30	53.7 (52.5-54.9)	53.4 (52.2-54.6)
40	47.7 (46.6-48.7)	47.7 (46.6-48.7)
50	41.7 (40.5-43.0)	41.9 (40.7-43.2)
60	36.1 (34.5-37.6)	36.4 (34.8-38.0)
70	30.7 (28.8-32.6)	31.2 (29.3-33.2)
80	25.9 (23.7-28.0)	26.5 (24.3-28.7)
90	21.5 (19.2-23.8)	22.2 (19.8-24.6)

For peer review only - http://bmjopen.bmj.com/site/about/guidelines.xhtml

Supplementary Table 7: Linear regression describing the influence of somatic passivity, thought interference and its subcomponents on the number of antipsychotics prescribed

Symptom	Standardized Beta Coefficients (95% CI)	t	Significance
Somatic Passivity	0.35 (0.24-0.45)	6.43	0.00
Thought Interference	0.40 (-0.14-0.22)	0.43	0.67
Thought Insertion	0.14 (0-0.01-0.30)	1.81	0.07
Thought Withdrawal	-0.00 (-0.17-0.16)	-0.05	0.96
Thought Broadcasting	-0.02 (-0.18-0.13)	-0.29	0.77

Adjusted for age at diagnosis, gender, ethnicity, diagnosis, IMD quartile and presence of paranoia, auditory hallucinations, persecutory delusion.

The RECORD statement – checklist of items, extended from the STROBE statement, that should be reported in observational studies using routinely collected health data.

	Item No.	STROBE items	Location in manuscript where items are reported	RECORD items	Location in manuscript where items are reported
**Title and abstract**					
	1	(a) Indicate the study's design with a commonly used term in the title or the abstract (b) Provide in the abstract an informative and balanced summary of what was done and what was found	Title page and abstract (p1&2)	RECORD 1.1: The type of data used should be specified in the title or abstract. When possible, the name of the databases used should be included.<br><br>RECORD 1.2: If applicable, the geographic region and timeframe within which the study took place should be reported in the title or abstract.<br><br>RECORD 1.3: If linkage between databases was conducted for the study, this should be clearly stated in the title or abstract.	Title page (p1) and abstract (p2)<br><br>Abstract (p2)<br><br>N/A
**Introduction**					
Background rationale	2	Explain the scientific background and rationale for the investigation being reported	Introduction (p3-5)		
Objectives	3	State specific objectives, including any prespecified hypotheses	Abstract (p2) and Introduction (p4&5, final paragraph)		
**Methods**					
Study Design	4	Present key elements of study design early in the paper	Methods (p6-9)		
Setting	5	Describe the setting, locations, and relevant dates, including periods of recruitment, exposure, follow-up, and data collection	Methods, (p6-9)		

For peer review only

Participants	6	*(a) Cohort study* - Give the eligibility criteria, and the sources and methods of selection of participants. Describe methods of follow-up<br>*Case-control study* - Give the eligibility criteria, and the sources and methods of case ascertainment and control selection. Give the rationale for the choice of cases and controls<br>*Cross-sectional study* - Give the eligibility criteria, and the sources and methods of selection of participants<br><br>*(b) Cohort study* - For matched studies, give matching criteria and number of exposed and unexposed<br>*Case-control study* - For matched studies, give matching criteria and the number of controls per case		RECORD 6.1: The methods of study population selection (such as codes or algorithms used to identify subjects) should be listed in detail. If this is not possible, an explanation should be provided.<br><br>RECORD 6.2: Any validation studies of the codes or algorithms used to select the population should be referenced. If validation was conducted for this study and not published elsewhere, detailed methods and results should be provided.<br><br>RECORD 6.3: If the study involved linkage of databases, consider use of a flow diagram or other graphical display to demonstrate the data linkage process, including the number of individuals with linked data at each stage.	Methods, subsections "context" and "patient sample" (p6)<br><br>Methods, subsection "exposure variables and software development" (p7&8)<br><br>N/A
Variables	7	Clearly define all outcomes, exposures, predictors, potential confounders, and effect modifiers. Give diagnostic criteria, if applicable.	Methods (p6-9)	RECORD 7.1: A complete list of codes and algorithms used to classify exposures, outcomes, confounders, and effect modifiers should be provided. If these cannot be reported, an explanation should be provided.	Methods (p6-9)
Data sources/ measurement	8	For each variable of interest, give sources of data and details of methods of assessment (measurement).<br>Describe comparability of assessment methods if there is more than one group	Methods (p6-9)		

For peer review only

Bias	9	Describe any efforts to address potential sources of bias	Methods, subsections "covariates" and "statistical analysis" (p8&9)		
Study size	10	Explain how the study size was arrived at	Methods, subsection "patient sample" (p6)		
Quantitative variables	11	Explain how quantitative variables were handled in the analyses. If applicable, describe which groupings were chosen, and why	Methods (p6-9)		
Statistical methods	12	(a) Describe all statistical methods, including those used to control for confounding<br>(b) Describe any methods used to examine subgroups and interactions<br>(c) Explain how missing data were addressed<br>(d) *Cohort study* - If applicable, explain how loss to follow-up was addressed<br>*Case-control study* - If applicable, explain how matching of cases and controls was addressed<br>*Cross-sectional study* - If applicable, describe analytical methods taking account of sampling strategy<br>(e) Describe any sensitivity analyses	Methods (p6-9)		
Data access and cleaning methods		..		RECORD 12.1: Authors should describe the extent to which the investigators had access to the database	Methods (p6-9)

For peer review only

				population used to create the study population.<br><br>RECORD 12.2: Authors should provide information on the data cleaning methods used in the study.	Methods (p6-9)
Linkage		..		RECORD 12.3: State whether the study included person-level, institutional-level, or other data linkage across two or more databases. The methods of linkage and methods of linkage quality evaluation should be provided.	N/A
**Results**					
Participants	13	(a) Report the numbers of individuals at each stage of the study (*e.g.*, numbers potentially eligible, examined for eligibility, confirmed eligible, included in the study, completing follow-up, and analysed)<br>(b) Give reasons for non-participation at each stage.<br>(c) Consider use of a flow diagram	Methods, subsection "patient sample" (p6)	RECORD 13.1: Describe in detail the selection of the persons included in the study (*i.e.,* study population selection) including filtering based on data quality, data availability and linkage. The selection of included persons can be described in the text and/or by means of the study flow diagram.	Methods, subsection "patient sample" (p6)
Descriptive data	14	(a) Give characteristics of study participants (*e.g.*, demographic, clinical, social) and information on exposures and potential confounders<br>(b) Indicate the number of participants with missing data for each variable of interest<br>(c) *Cohort study* - summarise follow-up time (*e.g.*, average and total amount)	Results (p10&11, table 1)		

For peer review only

Outcome data	15	*Cohort study* - Report numbers of outcome events or summary measures over time<br>*Case-control study* - Report numbers in each exposure category, or summary measures of exposure<br>*Cross-sectional study* - Report numbers of outcome events or summary measures	Results (p12&13, table 2)		
Main results	16	(a) Give unadjusted estimates and, if applicable, confounder-adjusted estimates and their precision (e.g., 95% confidence interval). Make clear which confounders were adjusted for and why they were included<br>(b) Report category boundaries when continuous variables were categorized<br>(c) If relevant, consider translating estimates of relative risk into absolute risk for a meaningful time period	Results (p14-19, tables 3-5)		
Other analyses	17	Report other analyses done—e.g., analyses of subgroups and interactions, and sensitivity analyses	Methods and Results (p6-19 - particularly subsection statistical analysis, p9; other tests also mentioned in Results where relevant)		
**Discussion**					
Key results	18	Summarise key results with reference to study objectives	Discussion (p20&21)		
Limitations	19	Discuss limitations of the study, taking into account sources of potential bias or imprecision.	Strengths and Limitations (p23-24)	RECORD 19.1: Discuss the implications of using data that were not created or collected to answer the specific research question(s). Include	Strengths and Limitations (p23-24)

For peer review only - http://bmjopen.bmj.com/site/about/guidelines.xhtml

		Discuss both direction and magnitude of any potential bias		discussion of misclassification bias, unmeasured confounding, missing data, and changing eligibility over time, as they pertain to the study being reported.	
Interpretation	20	Give a cautious overall interpretation of results considering objectives, limitations, multiplicity of analyses, results from similar studies, and other relevant evidence	Discussion (p20-23)		
Generalisability	21	Discuss the generalisability (external validity) of the study results	Strengths and Limitations (p23-24)		
**Other Information**					
Funding	22	Give the source of funding and the role of the funders for the present study and, if applicable, for the original study on which the present article is based	Funding and Conflict of Interest (p26)		
Accessibility of protocol, raw data, and programming code		..		RECORD 22.1: Authors should provide information on how to access any supplemental information such as the study protocol, raw data, or programming code.	Methods subsection "exposure variables and software Development" (p7): CRIS open-access online catalogue reference

*Reference: Benchimol EI, Smeeth L, Guttmann A, Harron K, Moher D, Petersen I, Sørensen HT, von Elm E, Langan SM, the RECORD Working Committee. The REporting of studies Conducted using Observational Routinely-collected health Data (RECORD) Statement. *PLoS Medicine* 2015; in press.

*Checklist is protected under Creative Commons Attribution (CC BY) license.
